# Which is better for mothers and babies: fresh or frozen-thawed blastocyst transfer?

**DOI:** 10.1186/s12884-020-03248-5

**Published:** 2020-09-23

**Authors:** Meiling Yang, Li Lin, Chunli Sha, Taoqiong Li, Wujiang Gao, Lu Chen, Ying Wu, Yanping Ma, Xiaolan Zhu

**Affiliations:** 1 Reproductive Medicine Center, The Fourth Affiliated Hospital of Jiangsu University (Zhenjiang Maternal and Child Health Hospital), 20 Zhengdong Road, Zhenjiang, Jiangsu 212001 People’s Republic of China; 2 Obstetrics and Gynecology, Nantong City No 1 People’s Hospital and Second Affiliated Hospital of Nantong University, Nantong, 226000 China; 3 Reproductive Sciences Institute, Jiangsu University, Zhenjiang, 212001 Jiangsu China

**Keywords:** Fresh blastocyst transfer, Frozen-thawed blastocyst transfer, Pregnancy outcome, Maternal complications, Neonatal outcomes

## Abstract

**Background:**

In recent years, there have been many reports on the pregnancy outcomes of fresh blastocyst transfer (BT) and frozen-thawed BT, but the conclusions are controversial and incomplete. To compare the pregnancy outcomes, maternal complications and neonatal outcomes of fresh and frozen-thawed BT in the context of in vitro fertilization or intracytoplasmic sperm injection (IVF/ICSI) cycles, we conducted a meta-analysis.

**Methods:**

A meta-analysis was conducted by searching the PubMed, Embase, and Cochrane Library databases through May 2020. Data were extracted independently by two authors.

**Results:**

Fifty-four studies, including 12 randomized controlled trials (RCTs), met the inclusion criteria. Fresh BT was associated with a lower implantation rate, pregnancy rate, ongoing pregnancy rate, and clinical pregnancy rate and higher ectopic pregnancy rate than frozen-thawed BT according to the results of the RCTs. The risks of moderate or severe ovarian hyperstimulation syndrome, placental abruption, placenta previa and preterm delivery were higher for fresh BT than for frozen-thawed BT. The risk of pregnancy-induced hypertension and pre-eclampsia was lower for fresh BT; however, no significant differences in risks for gestational diabetes mellitus and preterm rupture of membrane were found between the two groups. Compared with frozen-thawed BT, fresh BT appears to be associated with small for gestational age and low birth weight. No differences in the incidences of neonatal mortality or neonatal malformation were observed between fresh and frozen-thawed BT.

**Conclusions:**

At present there is an overall slight preponderance of risks in fresh cycles against frozen, however individualization is required and current knowledge does not permit to address a defintive response.

## Background

As cryopreservation technology has developed during the past few decades, the proportion of individual undergoing frozen blastocyst transfer (BT) has increased [1]. There have been concerns about the impact of cryopreservation on pregnancy outcomes, maternal complications and the health of the infant [2]. A few studies have compared pregnancy outcomes following fresh BT and cryopreserved-thawed BT in patients undergoing in vitro fertilization or intracytoplasmic sperm injection (IVF/ICSI) cycles [3–5]. However, the findings are controversial. A recent meta-analysis supported the hypothesis that single cryopreserved BT might not be a better choice than single fresh BT for patients undergoing IVF/ICSI [3]. However, another systematic review and meta-analysis suggested that pregnancy outcomes may be improved when frozen-thawed BT is performed [4].

With regard to maternal complications, Maheshwari et al. showed that frozen-thawed BT was associated with lower risks of postpartum haemorrhage (PH), placental abruption (PA), placenta previa (PP) and preterm delivery (PTD) than fresh BT and that the pregnancies arising from frozen-thawed BT seemed to have lower risks of maternal complications [2]. Shavit et al. reached the opposite conclusion that frozen-thawed BT may contribute to an increased risk of maternal complications such as preeclampsia and gestational diabetes mellitus (GDM) [6]. The latest randomized controlled trial reported that the incidence of preeclampsia was higher after frozen-thawed BT than after fresh BT, and the risk of moderate or severe ovarian hyperstimulation syndrome (OHSS) was similar in both groups [7].

Regarding the neonatal outcomes, an early review demonstrated that there were no significant differences in incidences of perinatal death or low birth weight of infants between fresh BT and frozen-thawed BT [8]. However, another review suggested that the incidences of small for gestational age (SGA), low birth weight (LBW), and perinatal mortality were lower in women who received frozen thawed BT [2].

There is growing concern regarding whether children born after frozen thawed BT have higher risks of congenital malformations than those born after fresh BT via IVF/ICSI. A register-based cohort study suggested that the risk for congenital malformation of the children born after frozen thawed BT was not higher than that of children born after fresh BT; in addition, no increased risks regarding the affected organ system were found between the two groups [9].

Due to limited sample sizes, past meta-analysis conclusions are controversial. With the emergence of new reports, there is an urgent need to perform a meta-analysis to compare the outcomes following fresh BT and frozen-thawed BT to provide guidance for clinical practice. The purpose of this meta-analysis was to examine pregnancy outcomes, maternal complications and neonatal malformations after frozen thawed BT versus those after fresh BT in an IVF/ICSI cycle and assess whether frozen thawed BT is a better choice than fresh BT.

## Methods

We followed the Preferred Reporting Items for Systematic Reviews and Meta-Analyses (PRISMA) guidelines when performing the systematic review and meta-analysis (Additional file 1).

### Eligibility criteria

The articles included compared clinical outcomes between patients undergoing IVF/ICSI cycles with fresh or frozen BT. Studies were excluded if they were duplicate publications, literature reviews or meta-analyses or if they had insufficient data for extraction.

### Search strategy

We searched the articles published in the PubMed, EMBASE and Cochrane Library databases through May 2020, using the following terms as key words: ‘humans’, ‘embryo*’, ‘cryo*’, ‘frozen’, ‘vitrif*’, ‘freez*’, and ‘fresh’. A comprehensive MEDLINE search strategy is presented in Additional file 2.

### Study selection and data extraction

Two researchers evaluated the selected studies and extracted the data independently. Any disagreement was resolved by discussion. First, we excluded the obviously ineligible literature by reading the title and abstract and then further read the full texts to identify the studies to be included. The data extracted mainly consisted of the first author, year of publication, study design, sample size, participants, intervention protocol, results, and other characteristics.

### Assessment of risk of Bias

We assessed the risk of bias of the included studies using RevMan 5.3.0 software and the risk of bias assessment of the Cochrane Collaboration [10], which assesses the generation of sequence allocation, allocation concealment, blinding, and incomplete outcome data for each trial included in the review. Each study was evaluated as having a high, low, or unclear risk of bias for each item. Any disagreements between the two reviewers were resolved by discussion with the corresponding author. The results of the assessment of risk of bias are shown in Additional file 3. Publication bias was checked by funnel plot if at least ten studies reported that outcome. Subgroup analysis was done by the design, cryopreservation type, endometrial preparation type, high ovarian response and single BT. Sensitivity analysis was employed to see the effect of single study on the overall estimation. Beyond subgroup analysis, meta-regression for the included studies was conducted to identify factors for heterogeneity.

### Outcome measures

The pregnancy outcomes were as follows: Implantation rate reflected the number of gestational sacs seen per embryo transferred. Pregnancy was identified according to increased serum hCG levels within 10 days after blastocyst transfer. Ongoing pregnancy was defined as pregnancy continuing beyond the 10th gestational week. Clinical pregnancy was considered the presence of a gestational sac with foetal heart activity, as assessed by ultrasound at 7 weeks of gestation. Miscarriage included any pregnancy that did not become an ongoing pregnancy. A multiple pregnancy was defined as a gestation with more than one foetus. The live birth rate was calculated as the number of birthing events per embryo transfer. We recorded the following maternal complications: GDM, pregnancy-induced hypertension (PIH), pre-eclampsia (PE), PTD, PP, PA, PH, and preterm rupture of membrane (PROM). PTD was defined as a liveborn or stillborn infant with a gestational age at birth of at least 28 weeks but less than 37 weeks. Very preterm delivery (VPTD) was defined as a liveborn or stillborn infant with a gestational age at birth of at least 28 weeks but less than 32 weeks. The neonatal outcomes included gestational age at delivery, birth weight, stillbirth, perinatal mortality and neonatal mortality. Large for gestational age (LGA) was defined as a birth weight above the 90th percentile. SGA was defined as birth weight below the 10th percentile. Very small for gestational age (VSGA) was defined as weighing below the 3rd percentile. High birth weight (HBW) was defined as a weight of > 4000 g at birth. Very high birth weight (VHBW) was defined as a weight of > 4500 g at birth. Low birth weight (LBW) was defined as a weight of < 2500 g at birth. Very low birth weight baby (VLBW) was defined as a weight of < 1500 g at birth. We also analysed neonatal malformations (NM), including congenital anomalies, chromosomal aberrations, and different organ system malformations.

### Statistical analysis

All statistical analyses were conducted using RevMan software. For the included studies, the results of dichotomous data for each of the studies eligible for meta-analysis were expressed as a risk ratio (RR) with the 95% confidence interval (CI). Although we analysed the data using both fixed effect models and random effect models, the results in the text are only from random effect models due to underlying heterogeneity in the studies. These results were combined for meta-analysis with the use of the Mantel/Haenszel model along with the random effects model. We assessed whether there was sufficient similarity between the eligible studies in regard to their design and clinical characteristics. Statistical heterogeneity was assessed with a chi-squared test and quantified with the I^2^ statistic. I^2^ > 50% was considered to indicate marked heterogeneity [11]. *p* < 0.05 was considered statistically significant.

## Results

### Study selection and characteristics

**Fig. 1:**
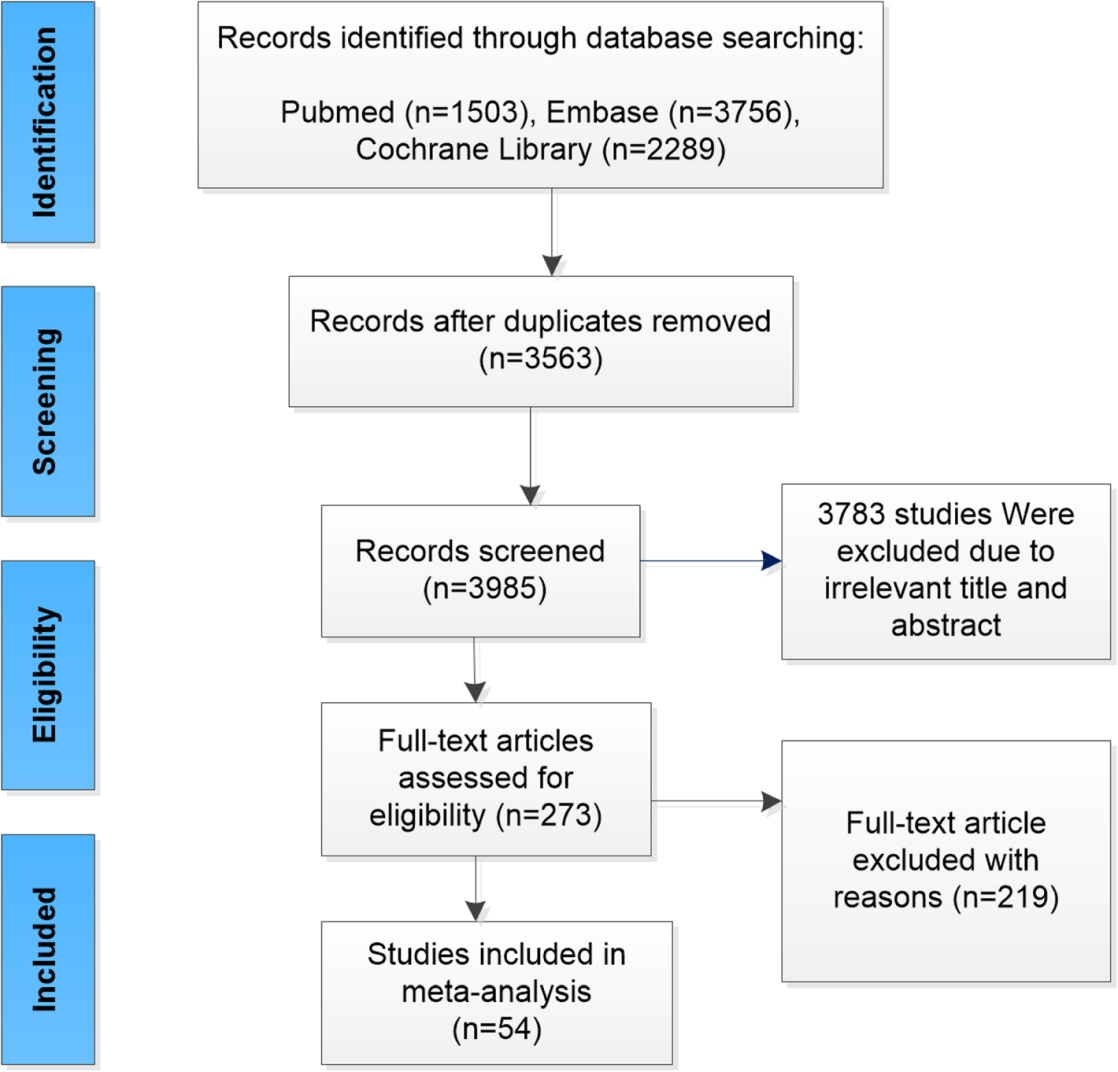
Flowchart showing the study selection process for the meta-analysis

A total of 3985 available publications were identified in our search. Of these, 3783 were excluded after reading the title and the abstract. Finally, 54 articles, including 12 randomized controlled trials (RCTs) and 42 nonrandomized controlled trials (NRCTs), were considered eligible by one or both reviewers (Fig. 1). Table 1 provides the details of all included studies.

**Table 1:** Characteristics of included studies

Author Year	Patients	Age (fresh/frozen)	Numbers of patient (fresh/frozen)	Design	Duration of trial	Outcomes
Aflatoonian 2010a [12]	Patients who were classified as high responders	28.1 ± 3.5/27.3 ± 4.4	187/187	Prospective RCT	Feb 2007 to Feb 2009	IR, CPR, MR, MPR, OPR
Aflatoonian 2010b [13]	Pregnancies after fresh ET vs. FET	29.9 ± 4.7/30.4 ± 4.5	500/200	Prospective RCT	Mar 2006 to Mar 2008	MR, LBR, PR, EPR, PTD, LBW,
Aghahosseini 2017 [14]	Infertile women with *P* ≥ 1.8 ng/dl	32.8 ± 5.8/30.5 ± 4.7	36/36	RCT	Jan to Apr 2016	CPR, MR, LBR, PR
Ainsworth 2019 [15]	Women who underwent fresh or FET at the Mayo Clinic	33.4 ± 4.4 /32.9 ± 3.6	87/ 49	Retrospective cohort study.	2010 to 2014	PPH, PE, SGA, GDM, PROM, PTD
Badalotti 2019 [16]	An assisted reproduction clinic in Brazil	No statement	1443/ 486	Retrospective case-control study	2010 to 2019	SGA, PTD, LBW, VLBW
Beltrananzola 2019 [17]	Infertile-couples undergoing IVF or IVF-ICSI	No statement	1857/ 352	Retrospective cohort study.	1995 to 2015.	GDM,hyper, Preterm, Very preterm,
Belva 2008 [18]	All pregnancies after transfer of FET	No statement	6402/1351	Unmatched cohort study	No statement	MR, MPR, LBR, OPR, PR, EPR, LBW, Stil
Bourdon 2018 [19]	Women with endometriosis infertile	34.3 ± 3.9/34.3 ± 4.1	135/135	Retrospective matched cohort study	Oct 2012 to Dec 2014	LBR, MPR, OPR
Cavoretto2020 [20]	Women undergoing fresh ET or FET	35.9 ± 3.9/34.9 ± 5.1	164 / 203	Prospective longitudinal study	Jan 2016 to Dec 2018	GDM, PTD, PE, SGA, LGA
Chen 2016 [21]	Infertile women with PCOS	28.2 ± 3.1/28.1 ± 3.0	762/746	Retrospective matched cohort study	Jun 2013 to May 2015	CPR, MR, LBR, OPR, PR, OHSS, PTD, PIH, Stil
Coates 2017 [22]	Patients undergoing IVF treatment using preimplantation genetic screening	36.6 (25–42)/ 36.7 (27–42)	88/91	RCT	Dec 2013 to Aug 2015	IR, MPR, LBR, OPR
Eum 2016 [23]	Women who underwent one or two fresh ET or FET	Using a cutoff of 35 years	69/206	Retrospective study	Jan 2013 to Dec 2014.	IR, CPR, MR, LBR, MPR
Fauque 2010 [24]	Women with adequate ovarian function	< 36 years	No statement	Prospective NRCT	2005 to 2007	CPR, MR, LBR, MPR, EPR.
Feng 2012 [25]	Women with various infertile causes	31.02 ± 3.69/ 31.60 ± 3.56	604/ 384	Retrospective study	Jan 2009 to Dec 2010	IR, CPR, MR, LBR, MPR, EPR, PTD, LBW, Stil, NM
Ferraretti 1999 [26]	Patients at risk of OHSS	31.46 ± 2.4/ 31.66 ± 2.8	67/58	Prospective RCT	Jan 1996 to Jul 1997	CPR, LBR, PR
Ginström 2019 [27]	Singletons born after in Sweden	34.9 ± 5.2 /34.1 ± 4.3	9726/24,365	Retrospective cohort study	2005 to 2015	GDM, PP, PA, PPH, PIH,
Healy 2010 [28]	Women with singleton births in Victoria Australia	No statement	4058/2045	Retrospective cohort study	1991 to 2004	PP, PA, PH
Henningsen 2011 [29]	Singleton born after fresh ET or FET	No statement	716/716	Retrospective cohort study	1994 to 2008	LBW
Henman 2005 [30]	Patients with three or more usable blastocysts	< 38 years	121/156	Prospective study	Apr 2000 to Dec 2001	IR, CPR, LBR, MPR
Hwang 2019 [31]	Singleton infants born to Massachusetts women who conceived by fresh or FET	No statement	12,390/ 2101	Retrospective cohort study.	July 2004, to Dec 2013	Preterm, LGA, SGA, LBW, Birth defects
Ishihara 2014 [32]	Women undergoing SET	Any age	33,559/118866 (5981/27408 singletons)	Retrospective study	2008 to 2010	CPR, MR, MPR, EPR, PP, PA, PIH, LGA, SGA, LBW, HBW, Stil
Johnson 2019 [33]	Women who underwent an autologous IVF cycle	35.8 (32.8–38.8)/36.3 (33.0–39.8)	1861/ 271	Retrospective cohort study	Jan 2000 to Jun 2015	SGA, PA
Korosec 2007 [34]	Women undergoing SET	< 37 years	65/214	Prospective study	Apr 2004 to Jun 2006	CPR
Le 2018 [35]	Non-PCOS women undergoing IVF/ICSI	Any age	391/391	RCT	Jun to Apr 2015	MR, LBR, EPR, GDM, PIH
Liu 2019 [36]	Women with 3 and 10 oocytes retrieved underwent fresh or FET	< 40 years	495/ 935	Retrospective cohort study	Jan 2015 to Jan 2019	CPR, MR, EPR
Magdi 2017 [37]	Women with recurrent implantation failure	<38 years	90/81	Prospective cohort study	Apr 2014 to Oct 2016	IR, CPR, MR, MPR, OPR, PR,
Maheshwari 2016 [38]	Singleton births after IVF/ICISI in the UK	Any age	95,911/16521	Retrospective cohort study	1991 to2011	PTD, LBW, HBW, NM
Martikainen 2001 [39]	Women with at least four good quality embryos	No statement	74/74	RCT	No statement	CPR, MR, LBR, EPR
Martikainen 2004 [40]	Women in the first or second treatment cycle when a top-quality embryo is available	<36 years	308/311	Retrospective cohort study	2000 to 2002	CPR, MR, MPR, LBR, OPR, EPR
Mizrachi 2019 [41]	Women with singleton IVF pregnancies who delivered	33.8 ± 5.5 /32.2 ± 5.2	74/57	Prospective cohort study	Dec 2017 to May 2019.	GDM, PE, PROM, SGA, LBW
Pelkonen 2010 [42]	Women underwent ART with ET leading to birth	Any age	3298/1852	Unmatched cohort study	1995 to2006	PP, PA, PH, PTD, LGA, LBW, SGA, Sti
Pelkonen 2014 [9]	Women underwent ART with ET leading to singleton births	Any age	2942/1830 (Singleton births)	Register-based cohort study	1995 to 2006	Major congenital anomalies
Pelkonen 2015 [43]	Women underwent ART with ET leading to singleton births	Any age	2933/1825	Register-based cohort study	1995 to 2006.	PTD, LGA, SGA, LBW, NM
Pinborg 2010 [44]	All singletons who according to the Danish IVF Register	Any age	10,392/5979	Retrospective cohort study	1995 to 2007	LBW, PTD, Stil, NM
Roy 2014 [45]	Infertile patients who underwent fresh ET or FET	No statement	1157/645	Retrospective study.	Mar2010 to Nov 2011	CPB, MR, LBR, PR, PTD, LBW
Serkan 2020 [46]	Infertile patients with various infertility etiology for IVF with fresh ET and sFET	18–42 years	620 /580	Retrospective cross-sectional study	Jun 2015 to May 2018	PR, CPR, MR, EPR, LBR
Shapiro 2011a [47]	Patients with first-time IVF cycle day 3 FSH < 10 mIU/mL and 8–15 antral follicles	<41 years	53/50	RCT	No statement	IR, CPR, MR, MPR, OPR, PR
Shapiro 2011b [48]	Patient must be undergoing her first IVF cycle; cycle day 3 FSH < 10 IU/L; and > 15 antral follicles	No statement	62/60	RCT	No statement	IR, CPR, MR, MPR, OPR, PR.
Shapiro 2013 [49]	Women with SET.	33.8 ± 4.7/33.8 ± 4.7	93/93	Matched cohort study.	Dec 2003 to Dec 2011	CPR, MR, MPR, OPR, PR
Shavit 2017 [6]	Singletons born after f fresh or FET	Any age	1886/1220	Retrospective cohort study	Dec 2008 to Dec 2012	CPR, MR, PA, PTD, GDM, PIH, LGA, SGA, LBW, HBW, NM
Shih 2008 [50]	Neonatal perinatal statistics unit Australia	Any age	3110/2387	Matched cohort study	1978 to 2005	PTD, LBW, NM
Vuong 2016 [51]	Non-PCOS infertile couples undergoing IVF/ICSI	No statement	391/391	RCT	No statement	MPR, LBR, MPR, OPR, EPR
Vuong 2018 [52]	Non-PCOS infertile women who were undergoing a first or second IVF cycle 3	32 ± 4 /32 ± 4	391/391	RCT	No statement	IR, CPR, MR, MPR, LBR, OPR, EPR,
Walls 2014 [53]	Women with PCOS.	Any age	122/179	Retrospective case–control study	Mar 2007 to Dec 2012.	CPR, MR, LBR, PR
Wang 2005 [54]	Infants conceived through ART procedures and born in Australia	Any age	7676/3824	Retrospective cohort study	1996 to 2000	LBW.
Wei 2019 [7]	Women with regular menstrual cycles undergoing their first cycle of in-vitro fertilization	28.8 (3.0) / 28·8 (3.0)	825/825	RCT	Aug 2016, to Jun 2017	R, CPR, MR, MPR, LBR, OPR, PR, EPR, PP, PH, PTD, GDM, PIH, LGA, SGA, NM
Wennerholm 1997 [55]	Birth after IVF with cryopreserved–thawed embryos in Sweden	34.0 ± 3.1/ 33.6 ± 3.3	209/209	Matched cohort study	Jun1990 to Jul 1995	PH, PTD, GDM, PIH, LBW
Wennerholm 2013 [56]	Singletons conceived after FET in Denmark, Norway and Sweden	33.3 ± 4.0/33.7 ± 3.9	42,242/6647	Retrospective cohort study	Until Dec 2007	PTD, LGA, SGA, VSGA, LBW, HBW,
Wikland 2010 [57]	Children born after vitrified BTor fresh BT	34.7 (22.0–44.0)/35.4 (26.3–45.3)	203/103	Retrospective cohort study	Jan 2006 to May 2008	MR, LBR, PR, EPR, PP, PA, GDM, PIH, LGA, SGA, LBW,
Wu 2014 [58]	High responder patients diagnosed as primary infertility with more than 15 oocytes retrieved	29.02 ± 2.87/ 29.05 ± 2.48	50/69	Retrospective cohort study	Jan to Nov 2012	IR, CPR, MPR,
Wu 2019 [59]	Patients with advanced endometriosis undergoing first embryo transfer cycles	No statement	255/ 506	Retrospective cohort study	Mar 2006 to Mar 2017	IR, CPR, MR, EPR, LBR, PTD, VPTD, LBW, VLBW
Xiong 2019 [60]	Women who underwent fresh or frozen ET	33.39 ± 3.75 /34.07 ± 4.14	101/ 179	Retrospective cohort study.	Aug 2014 to Jul 2017.	PR, IR, CPR, MR, MPR, OPR, LBR, SGA, LGA,
Yang 2015 [61]	Patients with *P* > 6 nmol/L on the HCG day in IVF/ICSI cycle	Between 20 and 40 years	43/42	Retrospective cohort study	Mar 2011 to Mar 2012	IR, CPR, LBR, PR,
Zhang 2018 [62]	Women with PCOS	28.1 ± 3.1/28.4 ± 2.9	212/250 (singletons)	Retrospective cohort study	Jun 2013 to Jul 2015	GDM, PE, PTD, LGA, SGA

*ET* Embryo transfer; *FET* Frozen embryo transfer; *IVF* In vitro fertilization; *ICSI* Intracytoplasmic sperm injection; *PCOS* Polycystic ovary syndrome; *RCT* Randomized controlled trial; *OHSS* Ovarian hyper-stimulation syndrome; *SET* Single embryo transfer; *P* Progesterone; *ART* Assisted reproductive technology; *IR* Implantation rate; *PR* Pregnancy rate; *OPR* Ongoing pregnancy rate; *EPR* Ectopic pregnancy rate; *LBR* Live birth rate; *MR* Miscarriage; *MPR* Multiple pregnancy rate; *PH* Postpartum haemorrhage; *PA* Placental abruption; *PP* Placenta previa; *PTD* Preterm delivery; *VPTD* Very preterm delivery; *PROM* Preterm rupture of membrane; *NM* Neonatal mortality; *PM* Perinatal mortality *PE* Pre-eclampsia; *PIH* Pregnancy-induced hypertension; *LGA* Large for gestational age; *SGA* Small for gestational age; *VSGA* Very small for gestational age; *HBW* High birth weight; *VHBW* Very high birth weight; *LBW* Low birth weight; *VLBW* Very low birth weight; *Stil* Stillbirth; *BD* Birth defects

### Pregnancy outcomes

**Fig. 2:**
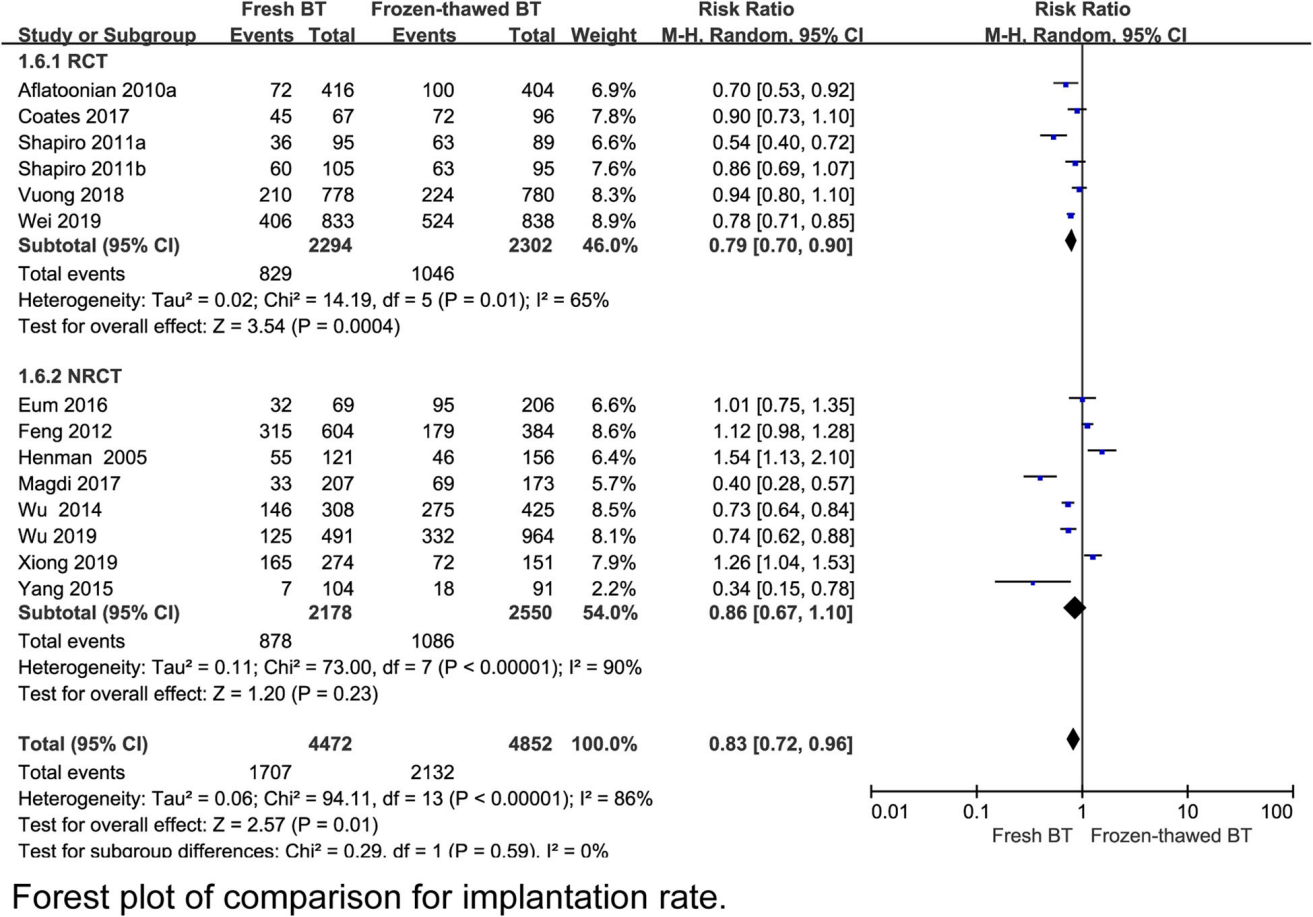
Forest plot of comparison for implantation rate

**Fig. 3:**
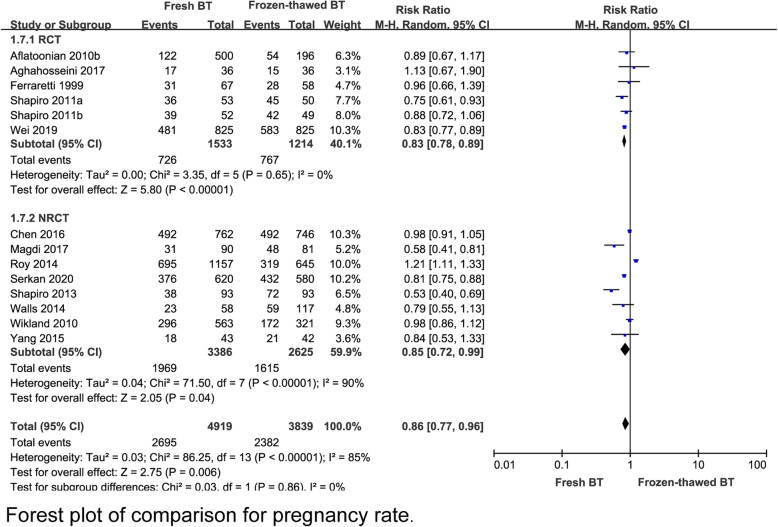
Forest plot of comparison for pregnancy rate

**Fig. 4:**
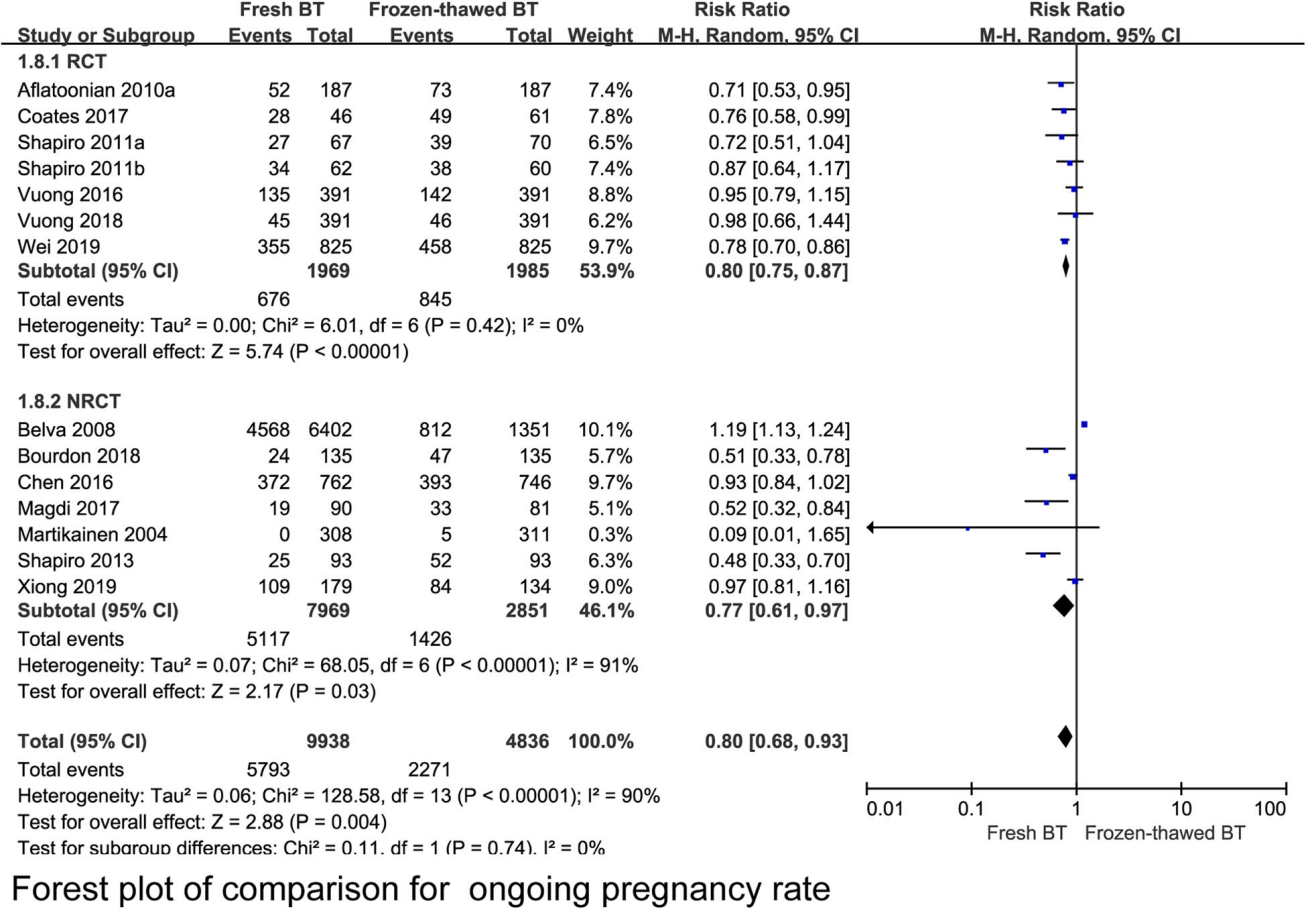
Forest plot of comparison for ongoing pregnancy rate

**Fig. 5:**
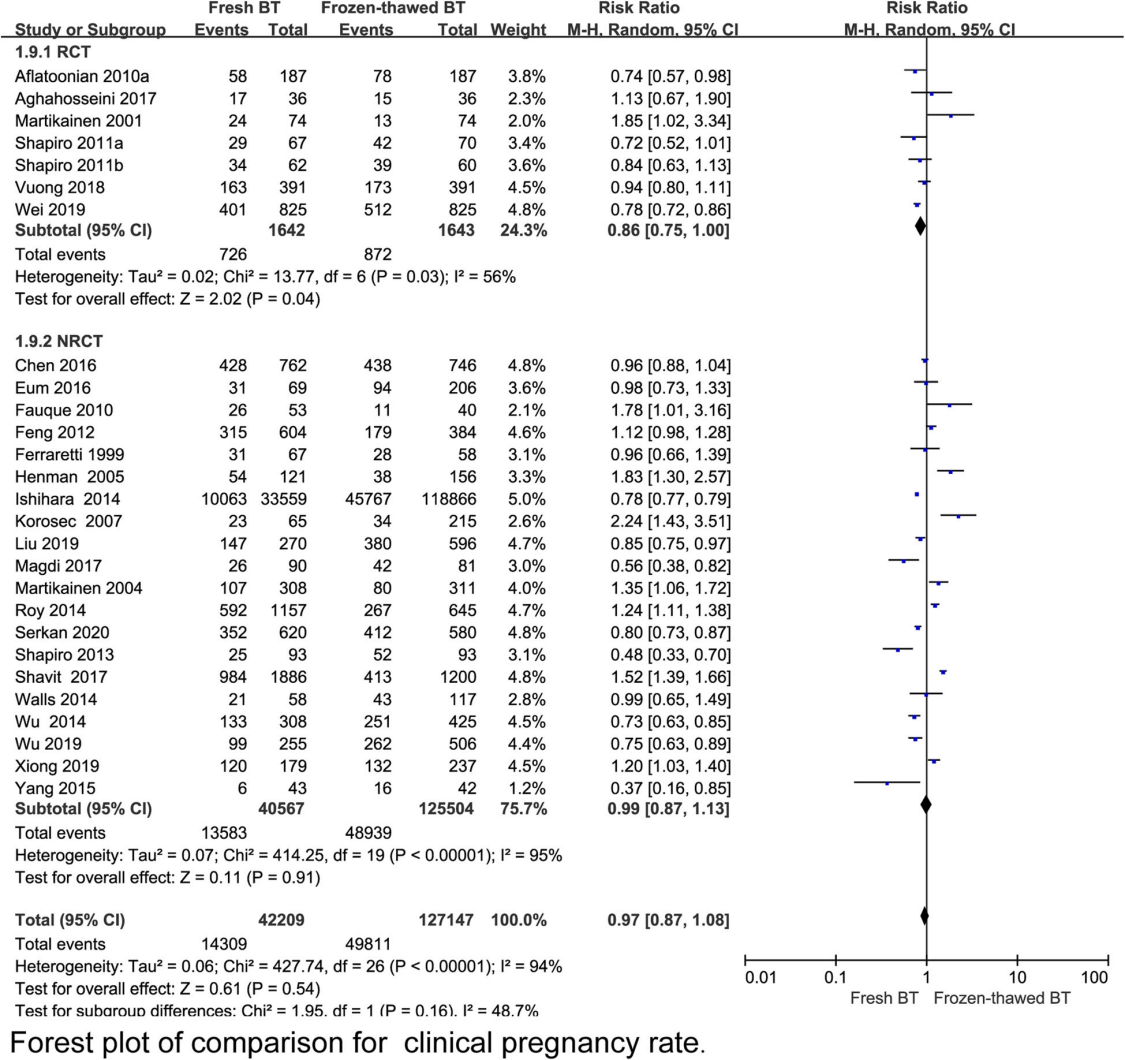
Forest plot of comparison for clinical pregnancy rate

**Fig. 6:**
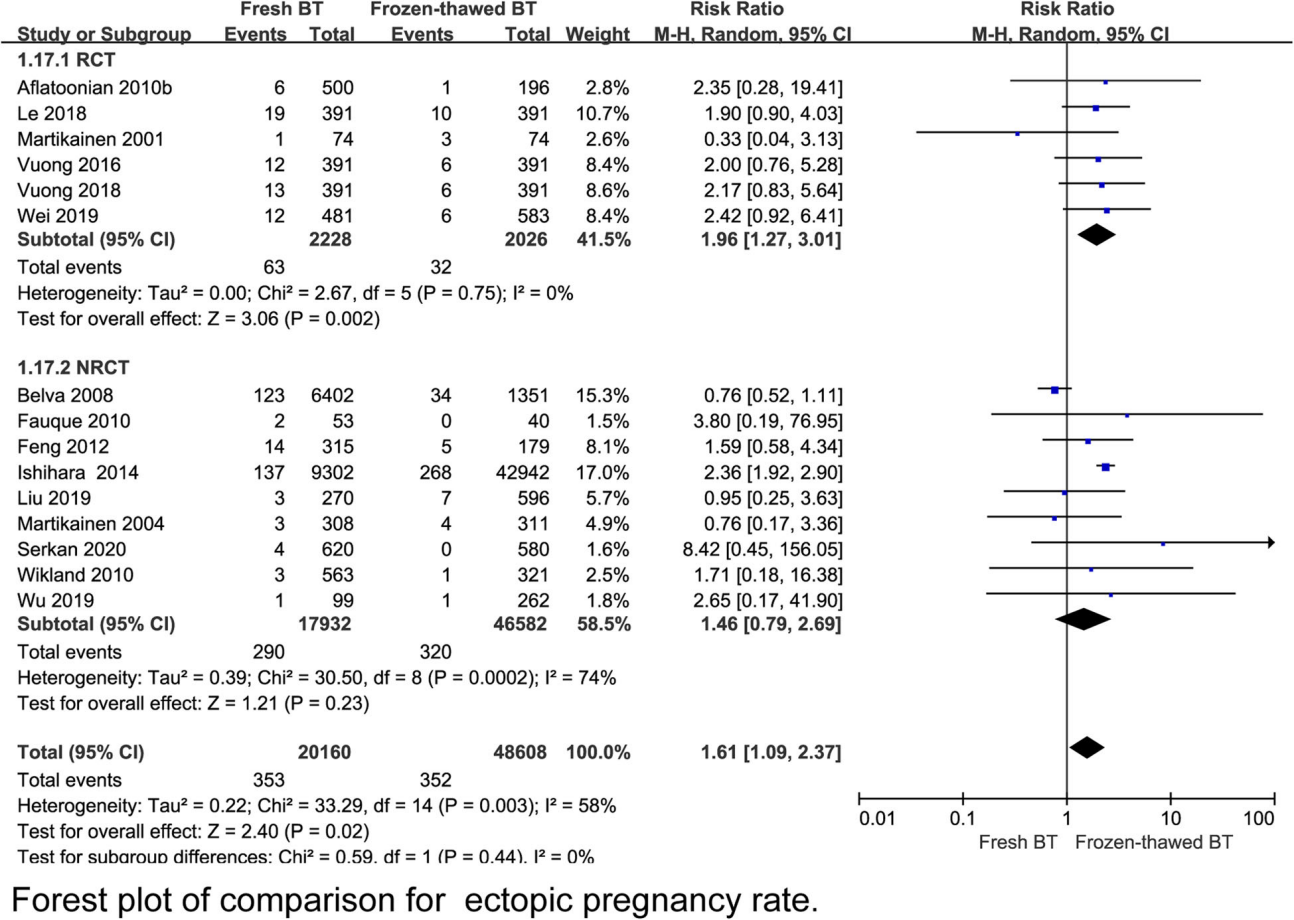
Forest plot of comparison for ectopic pregnancy rate

A total 6 RCT reported that the implantation rate (IR) was lower in the fresh BT group than in the frozen-thawed BT group (RR 0.79, 95% CI 0.70–0.90, *P* = 0.0004, heterogeneity: *I*^2^ = 65%) (Fig. 2). Compared with frozen-thawed BT, fresh BT was associated with a lower pregnancy rate (PR) according to 6 RCTs (RR 0.83, 95% CI 0.78–0.89, *P* < 0.00001, heterogeneity: *I*^2^ = 0%) (Fig. 3). Compared with women who underwent frozen-thawed BT, women who underwent fresh BT showed a decreased OPR according to 6 RCTs (RR 0.80, 95% CI 0.75–0.87, *P* < 0.00001, heterogeneity: I^2^ = 0%) (Fig. 4). The clinical pregnancy rate (CPR) was lower in the fresh BT group than in the frozen-thawed BT group according to 7 RCTs (RR 0.86, 95% CI 0.75–1.00, *P* = 0.04, heterogeneity: *I*^2^ = 56%) (Fig. 5). Six RCTs showed that fresh BT resulted in a significantly higher ectopic pregnancy rate (EPR) than frozen-thawed BT (RR 1.96, 95% CI 1.27–3.01, *P* = 0.002, heterogeneity: *I*^2^ = 0%) (Fig. 6). The above results indicate that frozen-thawed BT tends to result in higher IR, PR, OPR and CPR and lower EPR. According to the RCTs, the miscarriage rate (MR) (RR 0.86, 95% CI 0.65–1.13, *P* = 0.27, heterogeneity: *I*^2^ = 37%), MPR (RR 0.92, 95% CI 0.70–1.21, *P* = 0.56, heterogeneity: *I*^2^ = 30%) and LBR (RR 0.92, 95% CI 0.75–1.12, *P =* 0.41, heterogeneity: *I*^2^ = 87%) showed no statistically significant differences between fresh BT and frozen-thawed BT (Appendix 4) (See Additional file 4).

In conclusion, our meta-analysis showed that fresh BT was associated with lower IR, PR, OPR, and CPR and higher EPR than frozen-thawed BT. There were no differences observed in the MR, MPR or LBR between fresh and frozen-thawed BT.

### Maternal complications

**Fig. 7:**
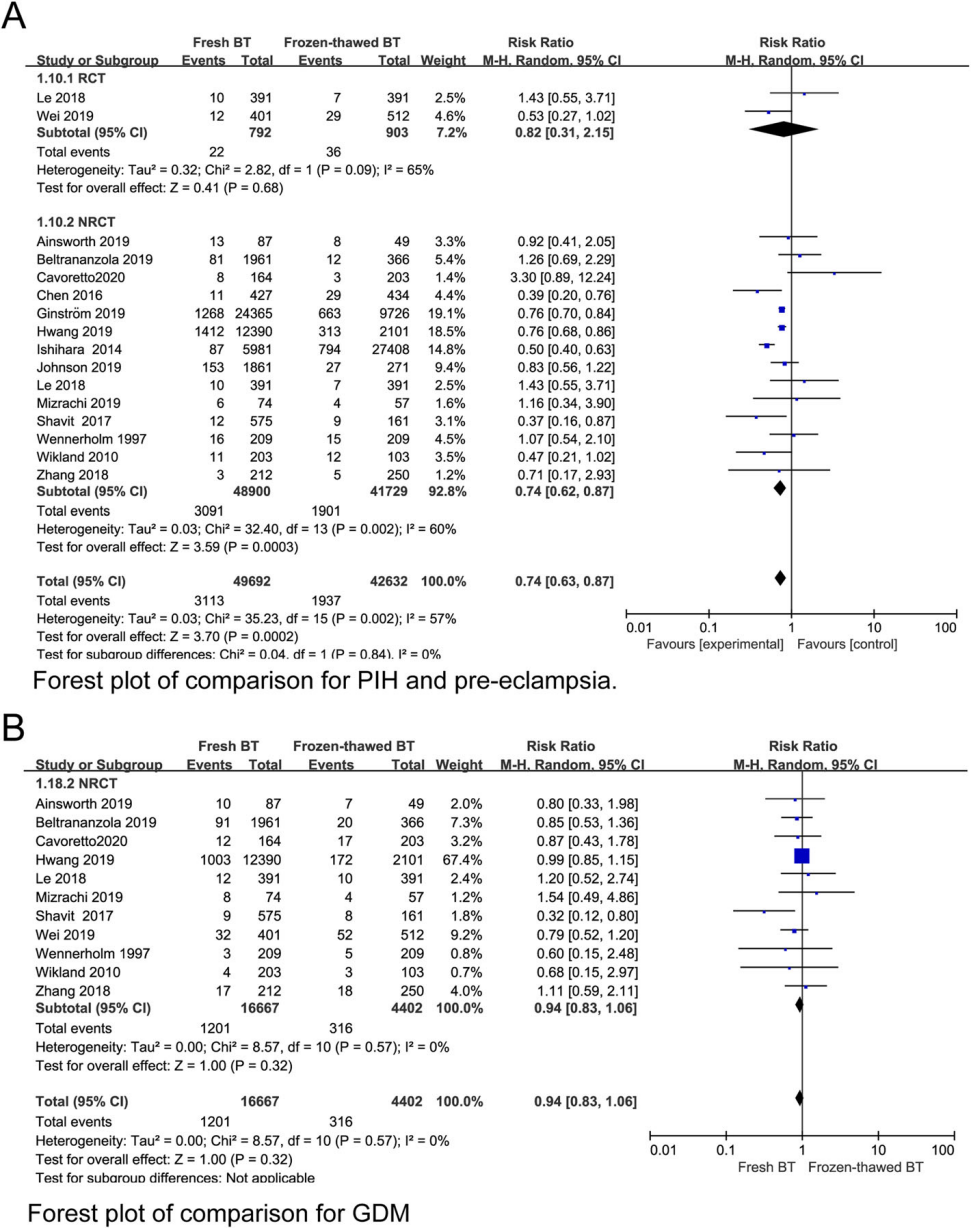
Forest plot of comparison for (**a**) PIH and pre-eclampsia and (**b**) GDM

**Fig. 8:**
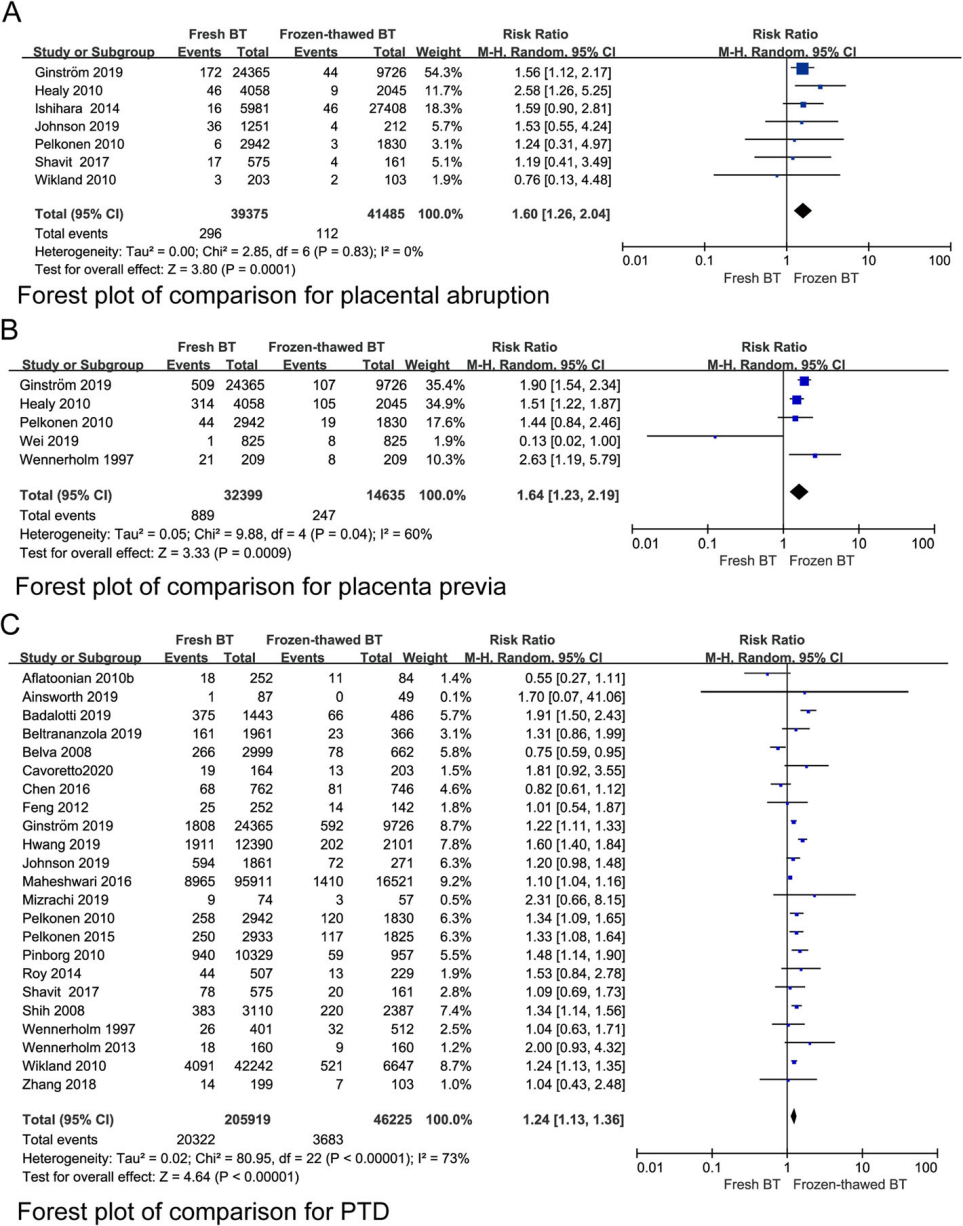
Forest plot of comparison for (**a**) placental abruption, (**b**) placenta previa and (**c**) PTD

To investigate whether fresh BT and frozen-thawed BT have different effects on maternal complications, we compared the incidence of PIH, pre-eclampsia, OHSS, PTD, GDM, PP, PA, PH, and PROM between the two groups. Lower incidences of PIH and pre-eclampsia were observed for fresh BT than for frozen-thawed BT (RR 0.74, 95% CI 0.63–0.87; *P* = 0.0002, heterogeneity: *I*^2^ = 57%) (Fig. 7a). The incidence of GDM showed no statistically significant differences between the two groups (RR 0.94, 95% CI 0.83–1.06; *P* = 0.32, heterogeneity: I^2^ = 0%) (Fig. 7b). Women who underwent fresh BT showed an increased risk of PA (RR 1.60, 95% CI 1.26–2.04; *P =* 0.0001, heterogeneity: I^2^ = 0%) (Fig. 8a). The risk of PP was higher for fresh BT than for frozen-thawed BT (RR 1.64, 95% CI 1.23–2.19; *P* = 0.04, heterogeneity: *I*^2^ = 60%) (Fig. 8b). Compared with the frozen thawed BT group, the fresh group had higher risks of PTD (RR 1.24, 95% CI 1.13–1.36, *P* < 0.00001, heterogeneity: *I*^2^ = 73%) (Fig. 8c) and VPTD (RR 1.27, 95% CI 1.14–1.54, *P* = 0.037, heterogeneity: *I*^2^ = 7%) (Appendix 5) (See Additional file 4). The incidence of PH (RR 1.00, 95% CI 0.57–1.74; *P* = 0.99, heterogeneity: *I*^2^ = 93%) and PROM (RR 1.05, 95% CI 0.77–1.45; *P* = 0.74, heterogeneity: *I*^2^ = 0%) did not differ significantly between fresh BT groups and frozen-thawed BT groups (Appendix 5) (See Additional file 4).

In summary, the risks of PIH and pre-eclampsia are lower for fresh BT than for frozen-thawed BT, while the risks of PA, PP and PTD for fresh BT are higher than those for frozen-thawed BT. However, the two groups had similar incidences of GDM, PROM and PH.

### Neonatal outcomes

**Fig. 9:**
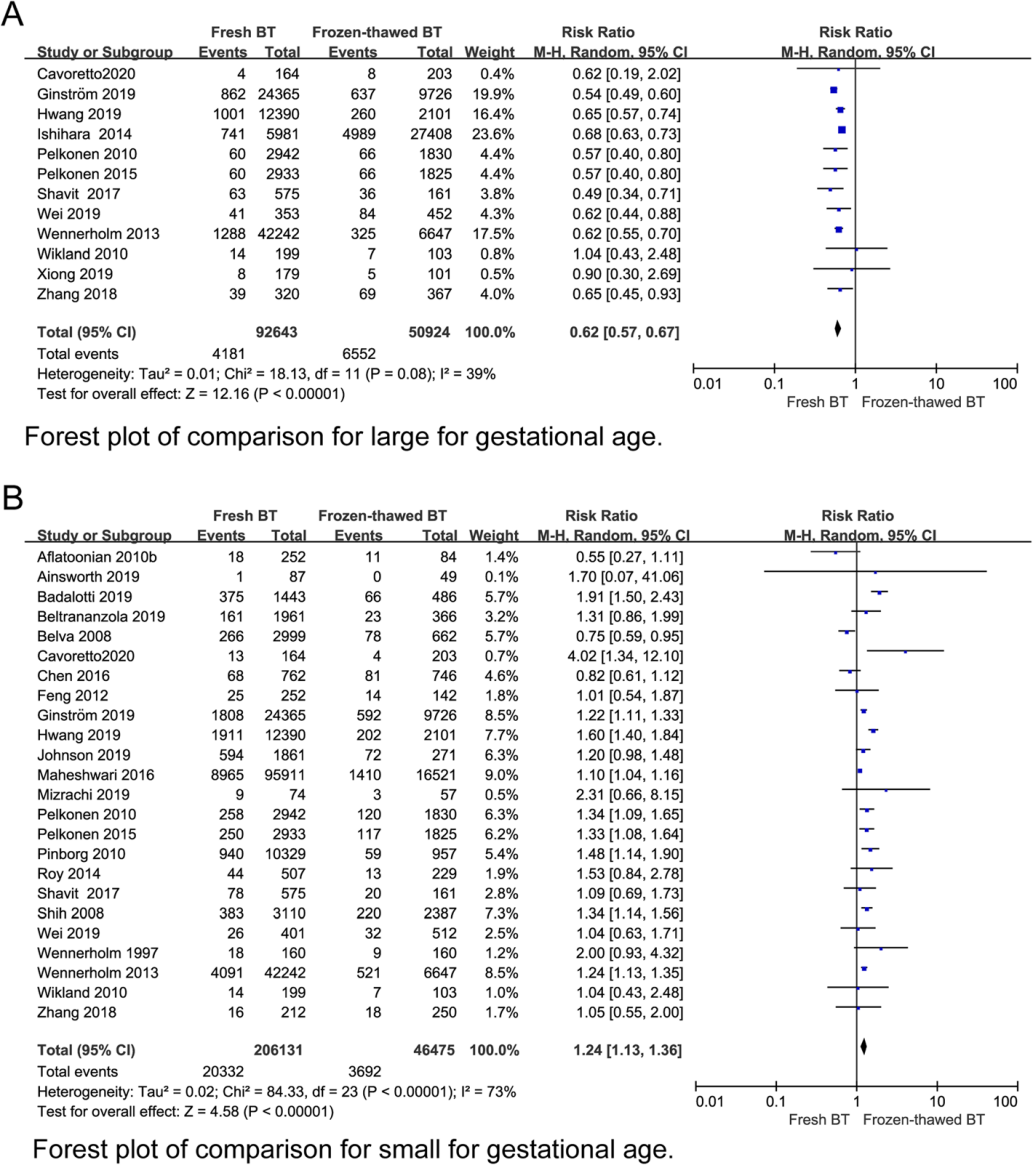
Forest plot of comparison for (**a**) large for gestational age, (**b**) small for gestational age

**Fig. 10:**
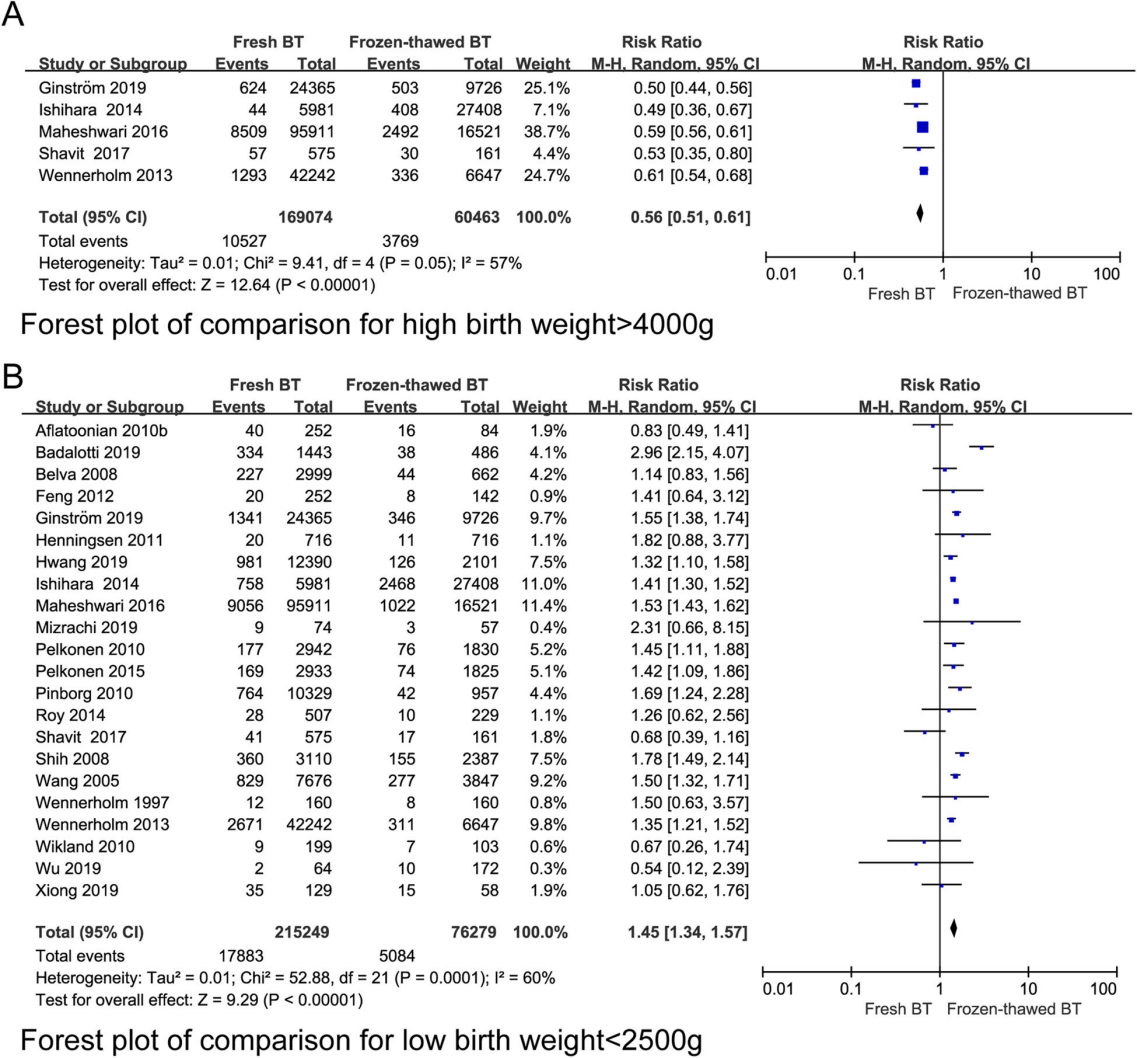
Forest plot of comparison for (**a**) high birth weight > 4000 g and (**b**) low birth weight < 2500 g

According to 12 studies, the LGA rate of the fresh BT group was lower (RR 0.62, 95% CI 0.57–0.67, *P* < 0.00001, heterogeneity: *I*^2^ = 39%) (Fig. 9a). However, the SGA rate of the fresh BT group was higher than that of the frozen-thawed BT group according to the data from the 23 included studies (RR 1.24, 95% CI 1.13–1.36, *P* < 0.00001, heterogeneity: *I*^2^ = 73%) (Fig. 9b). The RRs of HBW (RR 0.56 95% CI 0.51–0.61, *P* < 0.00001, heterogeneity: *I*^2^ = 57%) (Fig. 10a) and VHBW (RR 0.51, 95% CI 0.36–0.71, *P* < 0.0001, heterogeneity: *I*^2^ = 16%) (Appendix 6) (See Additional file 4) in the fresh BT group showed an absolute decrease compared with those in the frozen thawed BT group. In contrast, the risks of LBW (RR 1.45, 95% CI 1.34–1.57, *P* < 0.00001, heterogeneity: I^2^ = 60%) (Fig. 10b) associated with fresh BT showed an absolute increase when compared with those associated with frozen-thawed BT. No differences in VLBW (RR 0.97, 95% CI 0.55–1.70, *P* = 0.92 heterogeneity: *I*^2^ = 96%) were found (Appendix 6) (See Additional file 4). In addition, we investigated the differences in stillbirth, perinatal mortality and neonatal mortality between the two groups (Appendix 6) (See Additional file 4), and no significant differences were found.

In conclusion, fresh BT tends to lead to SGA and LBW, while frozen-thawed BT has the opposite effect. The stillbirth, perinatal mortality and neonatal mortality risks showed no statistically significant differences between the two groups.

### Neonatal malformations

**Fig. 11:**
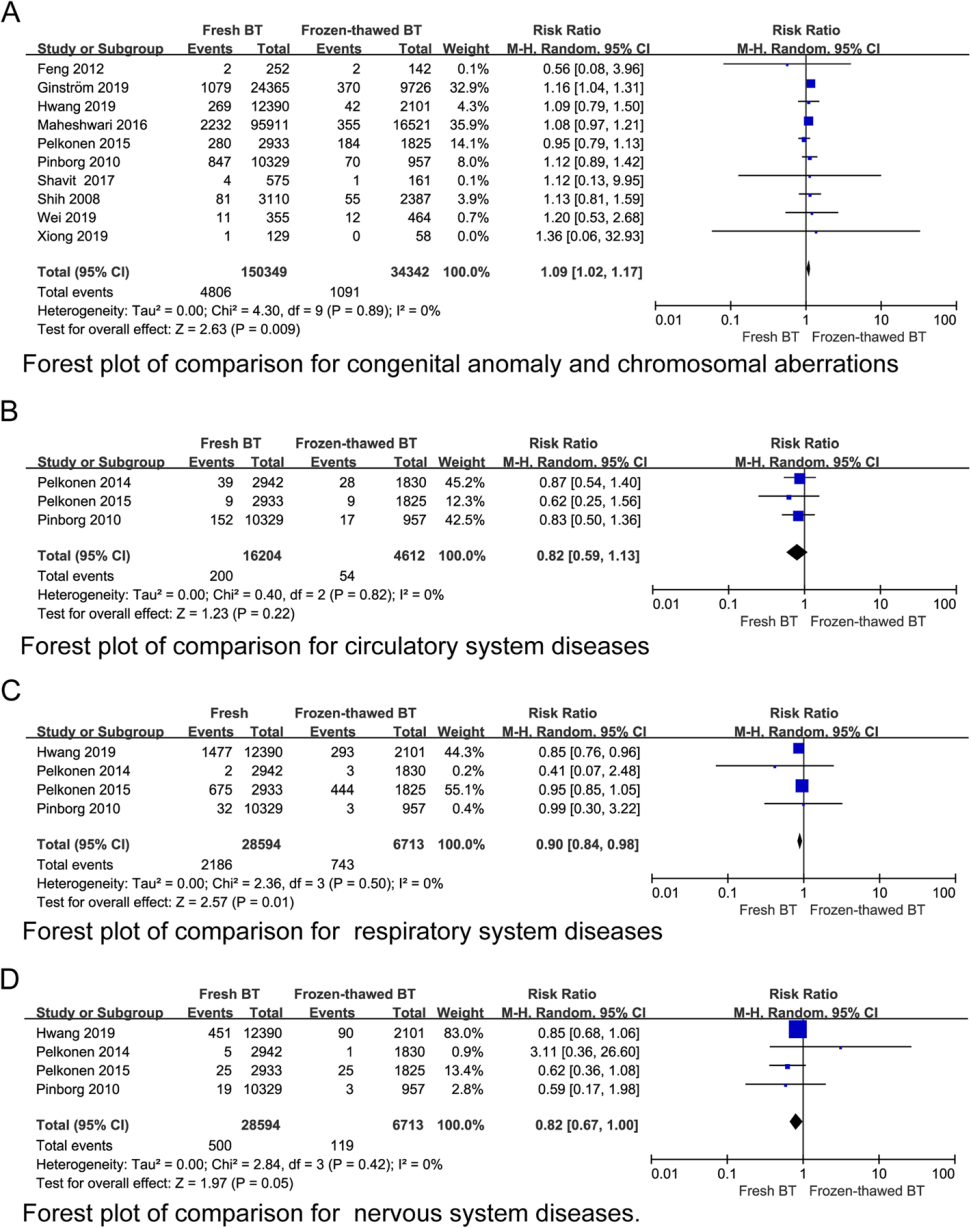
Forest plot of comparison for (**a**) congenital anomaly and chromosomal aberrations, (**b**) circulatory. System diseases, (**c**) respiratory system diseases and (**d**) nervous system diseases

From the data we have summarized, the risks of congenital anomalies and chromosomal aberrations of newborns associated with fresh BT showed an absolute increase when compared with frozen-thawed BT (RR 1.09 95% CI 1.02–1.17, *P* = 0.009, heterogeneity: *I*^2^ = 0%) (Fig. 11a). Further inspection of the risk of different organ system malformations in newborns, including malformations in the circulatory system (Fig. 11b), respiratory system (Fig. 11c), nervous system (Fig. 11d), gastrointestinal system, genitourinary system, eyes, ears, face, and musculoskeletal system (Appendix 7) (See Additional file 4), revealed no increased risk associated with frozen-thawed BT. The above data indicate that frozen-thawed BT is not a risk factor for neonatal malformations.

### Publication bias

The publication bias could be assessed by using either a funnel plot subjectively and Eggers regression test objectively in Additional file 5. For this review, funnel plots showed a symmetrical distribution. Egger’s regression test *p*-value on IR, PR, EPR, PIH &PE, GDM, PTD, LGA and SGA was more than 0.05 and which indicated the absence of publication bias. Egger’s tests yielding statistical evidence of publication bias at a *p*-value of OPR and CPR less than 0.05. In considering publication bias trim and fill meta-analysis was done. However, based on this analysis, no significant change was seen as compared with the main meta-analysis.

### Subgroup analysis

Subgroup analysis was performed according to the design of the origin trials, cryopreservation method, endometrial preparation method (See Additional file 6). When both RCTs and NRCTs were included, we also reached a similar conclusion of higher IR, PR, OPR for frozen thawed BT, higher EPR for fresh BT, and no differences in CPR, LBR, MR or MPR. When using vitrification for blastocyst cryopreservation in frozen-thawed BT, the trials revealed higher IR, OPR, LGA and lower EPR, SGA for frozen-thawed BT and no differences in PR, CPR, LBR, MR, LBW or stillbirth. Trials adopting the slow-freezing protocol showed higher IR, PR, PIH, pre-eclampsia and LGA and lower LBR, SGA for frozen thawed BT, and no differences in OPR, CPR, MR, EPR, LBW or stillbirth were found. When using the artificial cycle of endometrial preparation in fresh BT, the trials presented higher IR, PR, OPR, PIH and pre-eclampsia, LGA in. for frozen thawed BT and no differences in CPR, LBR, EPR, GDM, PA, PTD, SGA or LBW. The trials using the natural cycle showed higher IR, PR, CPR, and LGA, lower PA for frozen thawed BT and no differences in OPR, LBR, EPR, PIH, pre-eclampsia, GDM or PTD and lower SGA, LBW for frozen thawed BT. Moreover, there were a total of 4 studies analysing women with high ovarian response (See Additional file 7). Women with a high ovarian response who underwent frozen-thawed BT showed a higher IR, OPR and CPR and no difference in PR compared to women who underwent fresh BT. We also conducted a separate meta-analysis on single BT (See Additional file 8). Women who underwent single BT showed a higher OPR and lower EPR for frozen-thawed BT and no difference in IR, PR or CPR.

### Sensitivity analysis

Sensitivity analysis was done to identify outlier studies. According to the analysis, when performing meta-analysis on PR, Belva 2008 exceeded the upper CI limit so this trial was excluded (Appendix 38) (Additional file 9). When performing the remaining meta-analysis, no influential studies were detected so all of the studies were included in the final analysis.

### Meta regression

Beyond subgroup analysis, meta-regression for the included studies was conducted to identify factors for heterogeneity Meta-regression model in terms of natural cycle on IR, vitrification on PR, natural cycle on CPR, and slow-freezing on EPR was significant (*P* < 0.05) (Additional file 10). However, the meta-regression for the remaining results is not statistically significant.

## Discussion

Great advances have been made in cryopreservation culture techniques for embryos since the success of the first pregnancy resulting from frozen-thawed embryo transfer (FET) in 1983 [63]. This technique has been applied as a supplement to IVF and embryo transfer. FET has been accepted by every centre and has become an essential part of IVF/ICSI treatment. Therefore, the increased use of FET has increased the awareness of the safety of the technique [64]. This meta-analysis compared the outcomes of fresh BT and frozen-thawed BT in individuals undergoing IVF/ICSI cycles, with comprehensive assessment of the pregnancy outcomes, maternal complications, neonatal outcomes and malformations. Our study showed that frozen BT was associated with increased IR, PR, OPR, CPR, PIH, pre-eclampsia, LGA and HBW. Fresh BT was associated with increased EPR, OHSS, PP, PA, PTB, SGA, LBW, congenital anomalies and chromosomal aberrations in newborns. There were no differences in CPR, MR, MPR, LBR, PH or PROM between the two groups.

With respect to pregnancy outcomes, frozen-thawed BT was associated with higher IR, PR, OPR and CPR than fresh BT. There was no difference in MR, MPR, or LBR. Recently, Zeng et al. showed that there was no difference in IR, CPR, MR, or MPR, but an increased LBR was found for fresh BT compared with that of cryopreserved thawed BT [3]. Roque et al. showed that frozen-thawed BT significantly improved CPR and OPR in patients undergoing IVF/ICSI cycles [4]. In general, there is more evidence that frozen-thawed BT is associated with better pregnancy outcomes than fresh BT. There may be several reasons, which are described as follows. Embryo implantation is one of the important steps for reproductive success, and implantation failure remains an unsolved problem in the field of IVF/ICSI. The primary cause of failure is the impairment of endometrial receptivity (ER), rather than the embryo itself being responsible for the failure [65]. A study suggested that impaired ER is more likely to occur in fresh ET cycles after ovarian stimulation than in FET cycles with artificial endometrial preparation. Impaired ER apparently accounted for most implantation failures in the fresh group [47]. Another explanation for the association of better pregnancy outcomes with frozen BT is that the physical effects of freezing and thawing embryos may filter out weaker embryos and allow only good quality embryos to survive, resulting in better foetal growth [50]. The incidence of EPR between the two groups varied in different studies. The inconsistent conclusions may be related to differences in the data included. A previous study reported that EPR was higher for frozen thawed BT [66]. However, our study suggested that frozen thawed BT was related to lower EPR, which is consistent with these studies [67, 68]. The reason may be the high gonadotropin levels in ovarian stimulation cycles [68]. These findings also support the conclusions that a negative effect of ovarian stimulation on endometrial receptivity was reflected by increased EPR rates in the context of fresh BT.

From the perspective of maternal complications, our research demonstrated that the risks of PA, PP and PTD are lower for frozen-thawed BT than for fresh BT. In contrast, the risks of PIH and preeclampsia associated with frozen-thawed BT are higher than those associated with fresh BT. Blazquez et al. suggested that FET pregnancies carry a higher risk of PIH and pre-eclampsia than fresh ET pregnancies, possibly indicating that the protocols currently used for endometrial reception of embryos have a deleterious effect on placentation, perhaps due to the long exposure to hormone replacement [69]. OHSS is an iatrogenic condition resulting from an excessive ovarian response to superovulation medication. According to a previous meta-analysis, no difference was found in OHSS between fresh BT and frozen-thawed BT [70]. However, the previous data were insufficient. In our studies, 4 trials involving freezing cycles triggered with HCG showed that the risk of OHSS was higher for fresh BT than for frozen-thawed BT. A few recent reviews demonstrated that singleton pregnancies after the transfer of frozen-thawed embryos were associated with lower risks of PTD and VPTD than those after fresh embryo transfer, which is in agreement with our research [2, 71–73]. A recent meta-analysis showed that spontaneous preterm birth (SPTB) is increased in individuals undergoing IVF/ICSI [74]. The subgroup sensitivity analysis excluding FET revealed a higher risk of SPTB than that in the general group, supporting the hypothesis of lower prematurity risk in pregnancies from FET [74]. A recent meta-analysis of cohort studies showed that it is possible that a shift toward FET will determine a reduction in iatrogenic preterm birth. However, the authors advocated caution and further prospective studies to avoid the risk of increasing other negative obstetrics outcomes [75].

In regard to neonatal outcomes, our study suggested that there were lower risks of SGA and LBW in singleton pregnancies after frozen-thawed BT than in those after fresh BT, which was consistent with the previous meta-analysis [2]. However, the stillbirth and perinatal mortality and neonatal mortality rates were not significantly different between the two groups. Moreover, with respect to neonatal malformations, there were no differences between fresh BT and frozen-thawed BT. In conclusion, singleton pregnancies after frozen-thawed BT seem to have better neonatal outcomes than those after fresh BT, owing to lower risks of SGA and LBW. The reasons for the association of better neonatal outcomes with frozen BT than with fresh BT are not yet known. Evidence from a recent original study showed that uterine Doppler studies in pregnancies from frozen-thawed blastocysts present an approximately 15% lower uterine artery pulsatility index than those from fresh blastocysts, with a lower risk of SGA in the fresh group than in the frozen group, which is consistent with our conclusion [20] . In contrast to IVF with fresh embryo transfer, FET is usually performed in minimally stimulated or natural cycles. This lowers the risk of SGA and LBW after FET, which may be attributed to a luteal phase that mirrors the natural cycle, with favourable effects on the endometrium and early implantation [72]. Another probable explanation is related to the finding that controlled ovarian hyperstimulation (COH) was associated with poorer neonatal outcomes assessed by SGA and LBW in a recent study [76]. The results favouring frozen thawed BT instead of fresh BT may relate to the adverse effects of COH on ER [48, 77]. Therefore, elective cryopreservation of viable embryos could be an alternative to avoid the deleterious effects of COH in embryo endometrium synchrony [12, 47].

### Strengths and limitations

The major strength of this systematic review is the comprehensive literature search, identifying study subjects from a substantial number of relevant publications; another strength is the many pregnancy outcomes, maternal complications and neonatal outcomes the study evaluated between frozen and fresh BT to explore which method is better for mothers and infants. In addition, we conducted RCT and NRCT meta-analyses separately to improve the quality of evidence. However, the present meta-analysis also has some limitations, one of which was the significant heterogeneity in the pregnancy outcomes in the meta-analysis. We tried to find the source of heterogeneity by performing a subgroup analysis to examine the source of heterogeneity but were not able to identify the source. In addition, the baseline characteristics of patients differed among the included studies, including country, age, smoking status, duration of infertility, type of infertility, endometrial thickness, and cryopreservation type.

## Conclusions

In summary, considering the higher IR, PR, OPR, and CPR; lower EPR; and decreased risks of OHSS, PA and PTD, as well as the incidences of SGA and LBW associated with frozen-thawed BT, there is an overall slight preponderance of risks in fresh cycles against frozen. However, frozen-thawed BT also has potential risks, such as LGA, PIH and pre-eclampsia. During the freezing process, there is a trade-off between the risk of pre-eclampsia hypertension and all other higher risks of fresh tranfers. In conclusion, the present review assessed the risks and magnitudes associated with fresh or frozen-thawed transfers, however it could not provide a final answer on the research question in object in the title. Therefore, individualization is required in order to choose the best procedure for each specific case according to the clinical needs.

## Supplementary information

**Additional file 1: Appendix 1.** PRISMA checklist.

**Additional file 2: Appendix 2.** Sample search strategy used for PubMed.

**Additional file 3: Appendix 3.** Risk of bias graph.

**Additional file 4: Appendices 4–7.** Supplemental Figures.

**Additional file 5: Appendices 8–18.** Publication bias.

**Additional file 6: Appendix 19–34.** Subgroup analysis.

**Additional file 7: Appendix 35.** Women with high ovarian response.

**Additional file 8: Appendix 36.** Single blastocyst transfer.

**Additional file 9: Appendix 37–49.** Sensitivity analysis.

**Additional file 10: Appendix 50.** Meta regression.

## Data Availability

Not applicable.

## Abbreviations

BT: Blastocyst transfer
PH: Postpartum hemorrhage
PA: Placental abruption
PP: Placenta previa
GDM: Gestational diabetes mellitus
OHSS: Ovarian hyperstimulation syndrome
SGA: Small for gestational age
LBW: Low birth weight
PIH: Pregnancy-induced hypertension
LGA: Large for gestational age
VSGA: Very small for gestational age
HBW: High birth weight baby
VHBW: Very high birth weight baby (VHBW)
RR: Risk ratio
CI: Confidence intervals
RCT: Randomized controlled trials
NRCT: Non-randomized controlled trials
IR: Implantation rate
PR: Pregnancy rate
OPR: Ongoing pregnancy rate
EPR: Ectopic pregnancy rate
LBR: Live birth rate
MR: Miscarriage
MPR: Multiple pregnancy rate
