# Supplementary material for: Which is better for mothers and babies: fresh or frozen-thawed blastocyst transfer?
Source: BMC Pregnancy Childbirth. 2020 Sep 23;20:559. doi: 10.1186/s12884-020-03248-5 (PMC7513314; doi:10.1186/s12884-020-03248-5)
Supplement: Supplementary file 2 — Additional file 2: Appendix 2. Sample search strategy used for PubMed. [file 12884_2020_3248_MOESM2_ESM.docx]

| Database: Pubmed  Date of last search: May 2020 |
| --- |
| Search strategy:  (("Humans"[MeSH Terms] AND "embryo*"[Title/Abstract]) AND ((("freez*"[Title/Abstract] OR "vitrif*"[Title/Abstract]) OR "frozen*"[Title/Abstract]) OR "cryo*"[Title/Abstract])) AND "fresh"[Title/Abstract] |
| Number of results: 1503 |

Additional file 2: Electronic search strategy
