# Supplementary figures and images for: Which is better for mothers and babies: fresh or frozen-thawed blastocyst transfer?
Source: BMC Pregnancy Childbirth. 2020 Sep 23;20:559. doi: 10.1186/s12884-020-03248-5 (PMC7513314; doi:10.1186/s12884-020-03248-5)

**Appendix 3:** Risk of bias graph
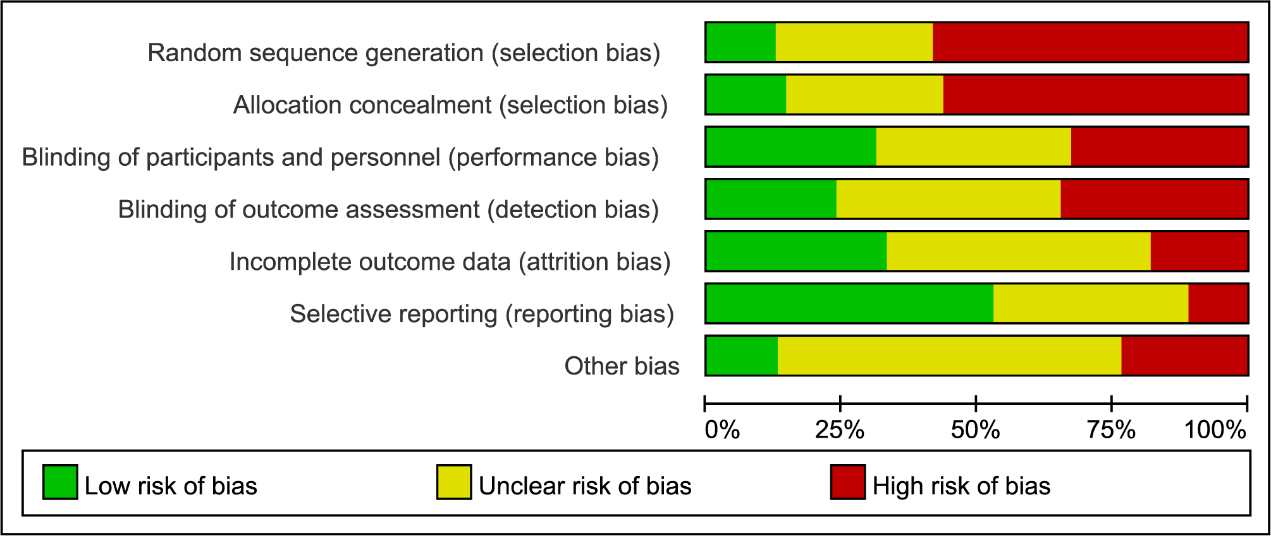

Supplement: Supplementary file 3 — Additional file 3: Appendix 3. Risk of bias graph. [file 12884_2020_3248_MOESM3_ESM.docx]
