# Supplementary material for: Which is better for mothers and babies: fresh or frozen-thawed blastocyst transfer?
Source: BMC Pregnancy Childbirth. 2020 Sep 23;20:559. doi: 10.1186/s12884-020-03248-5 (PMC7513314; doi:10.1186/s12884-020-03248-5)
Supplement: Supplementary file 4 — Additional file 4: Appendices 4–7. Supplemental Figures. [file 12884_2020_3248_MOESM4_ESM.docx]

Supplemental Figures

A


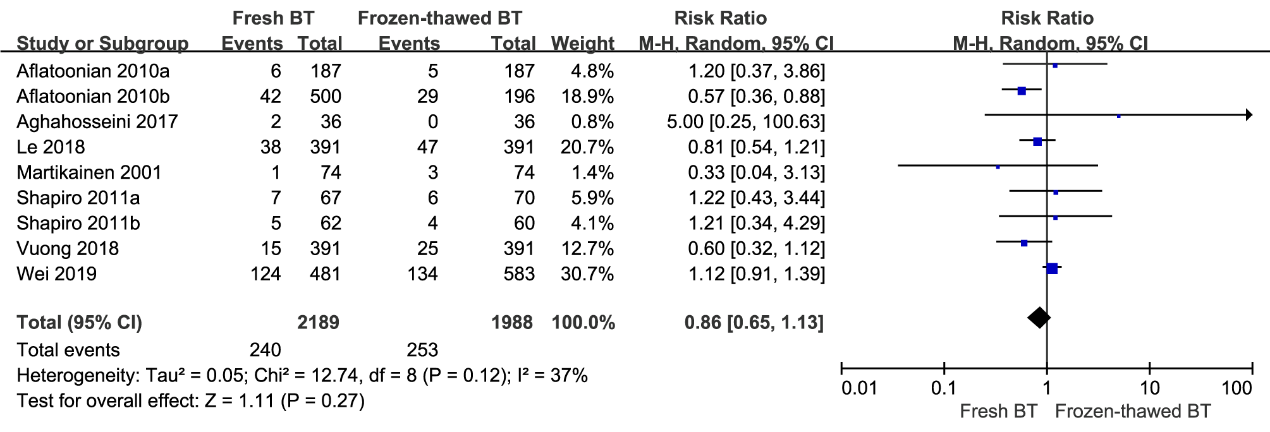


B


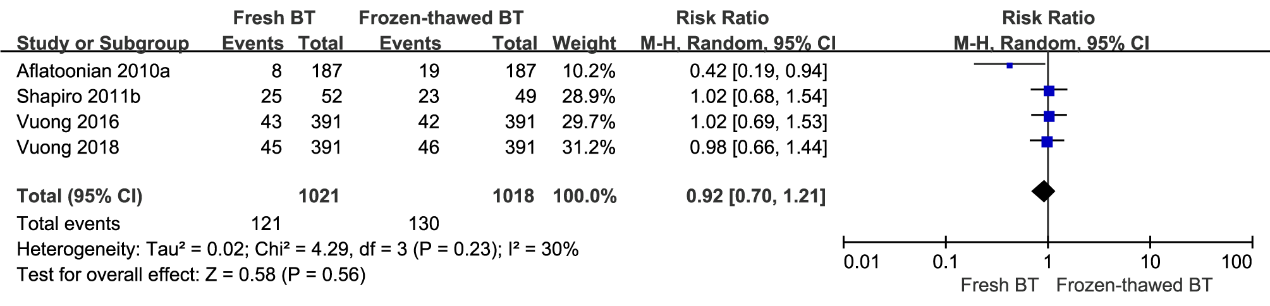


C


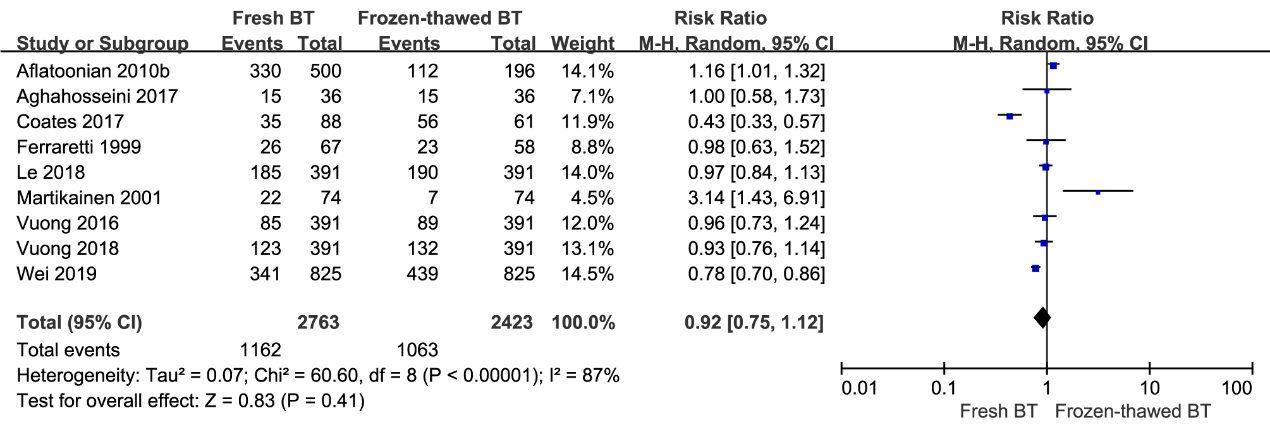


**Appendix 4**: Forest plot of comparison for (a) micarriage rate, (b) multiple pregnancy rate and (c) live birth rate

A


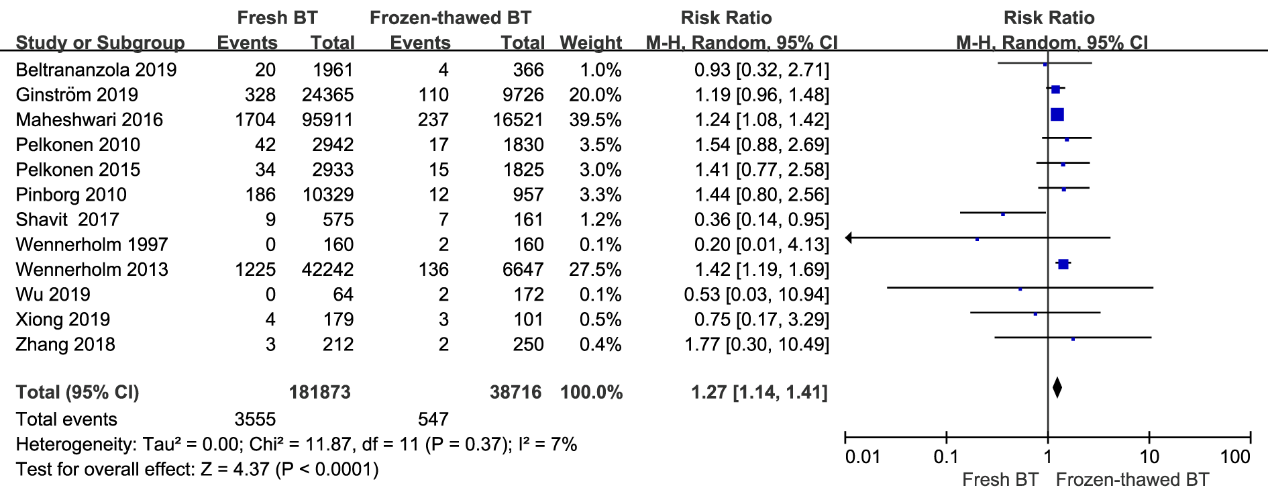


B


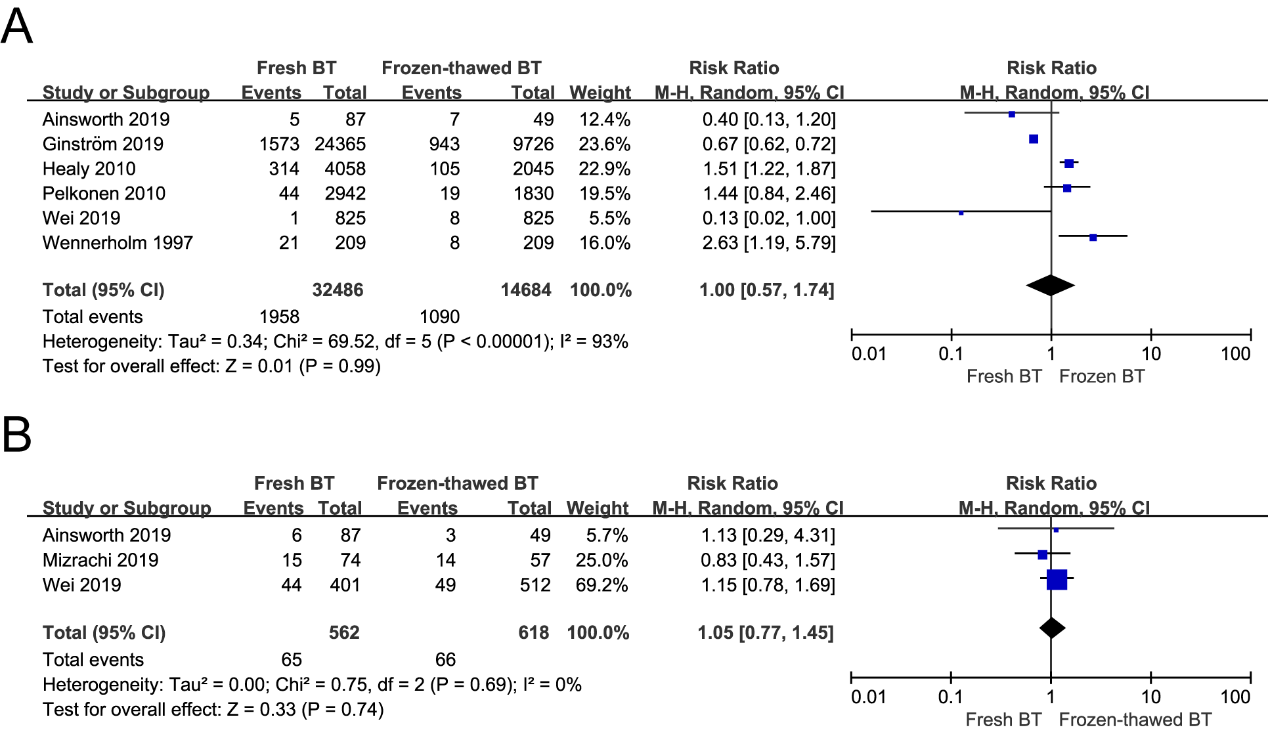


C


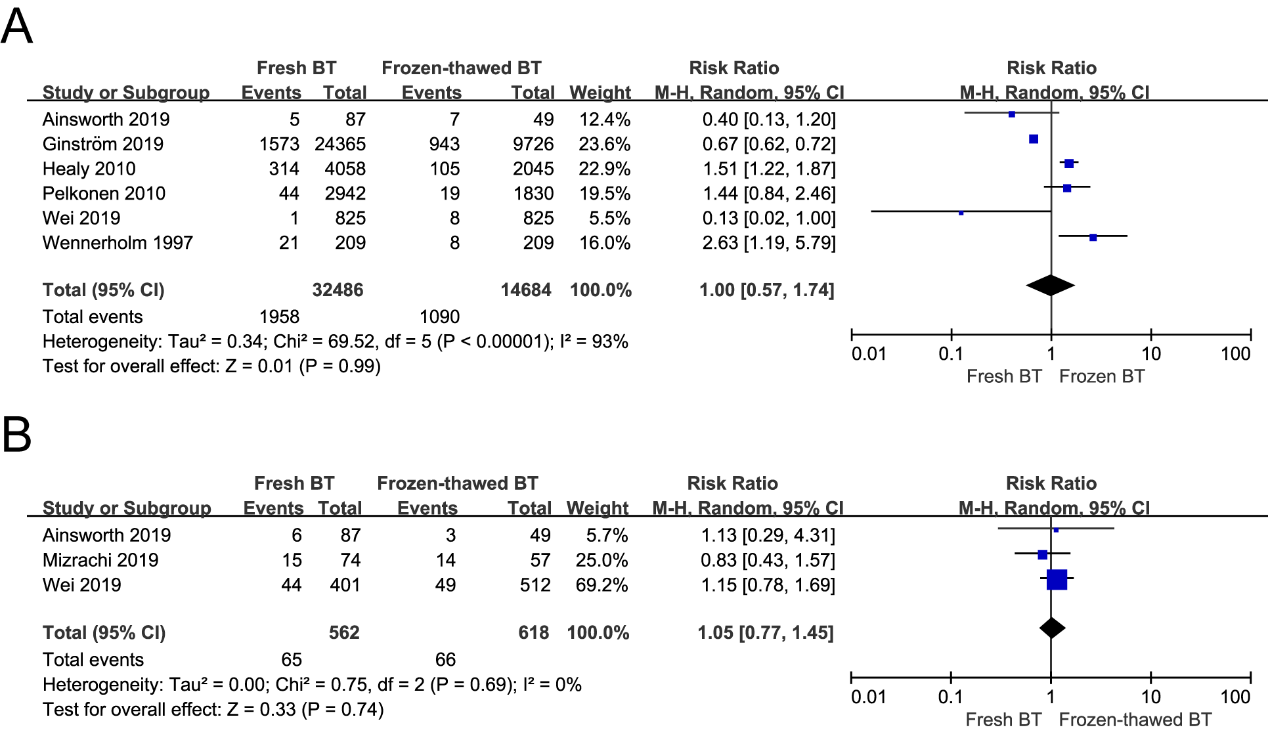


**Appendix 5**: Forest plot of comparison for (a) very preterm<32W, (b) post-partum haemorrhage and (c) preterm rupture of membrane

A


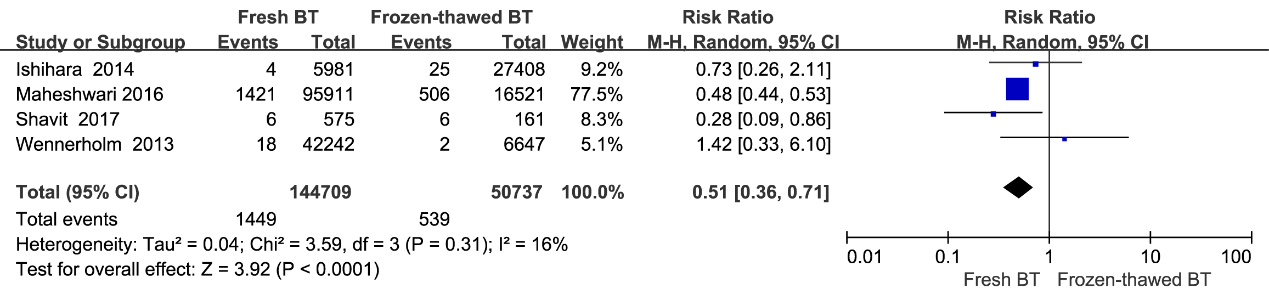


B
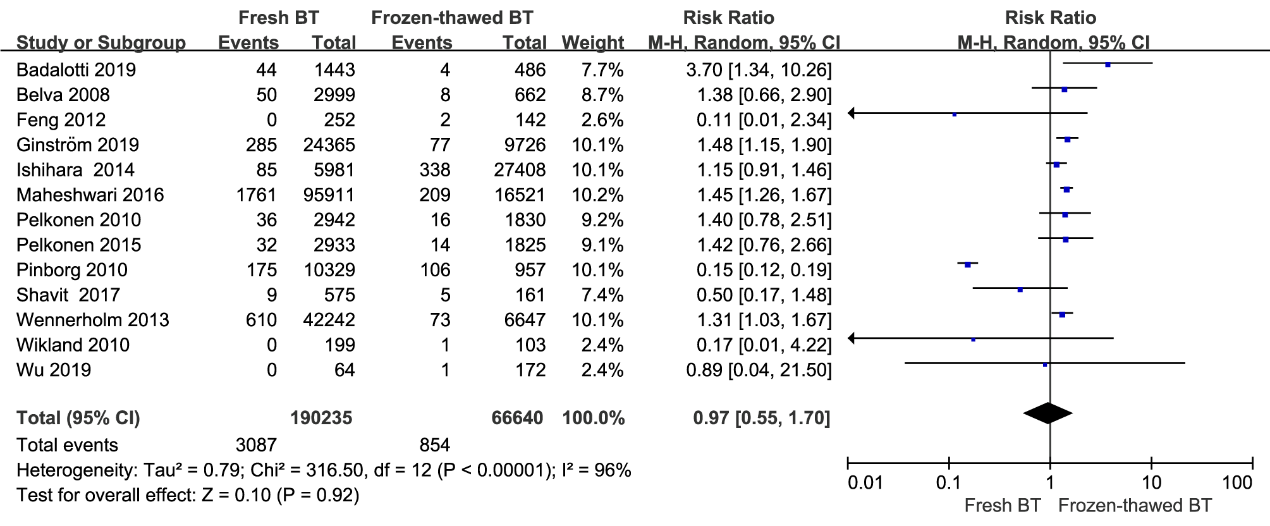


C
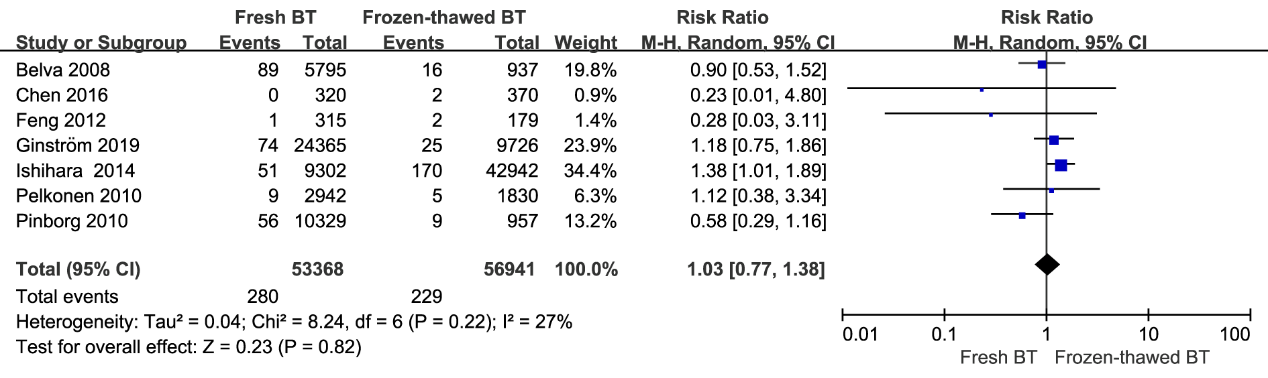


D
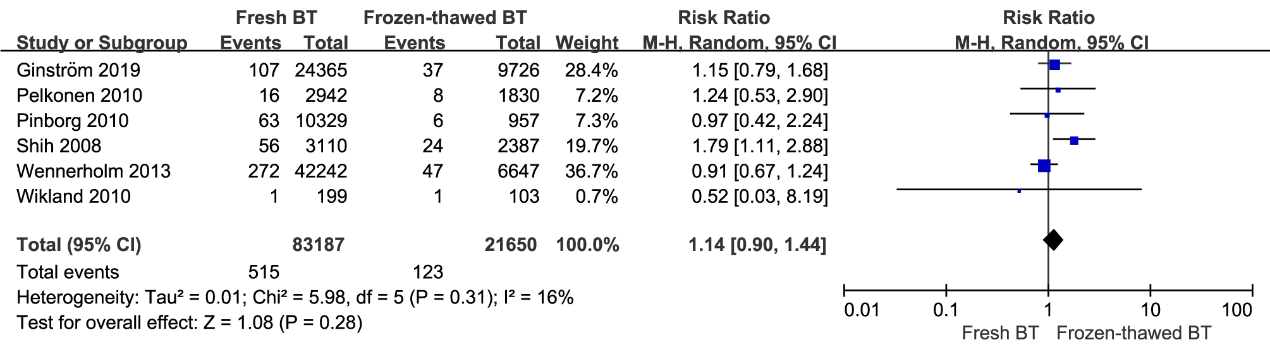


**Appendix 6**: Forest plot of comparison for (a) very high birth weight > 4500g,
(b) very low birth weight < 1500g, (c) stillbirth, (d) perinatal mortality and (e) neonatal mortality

A
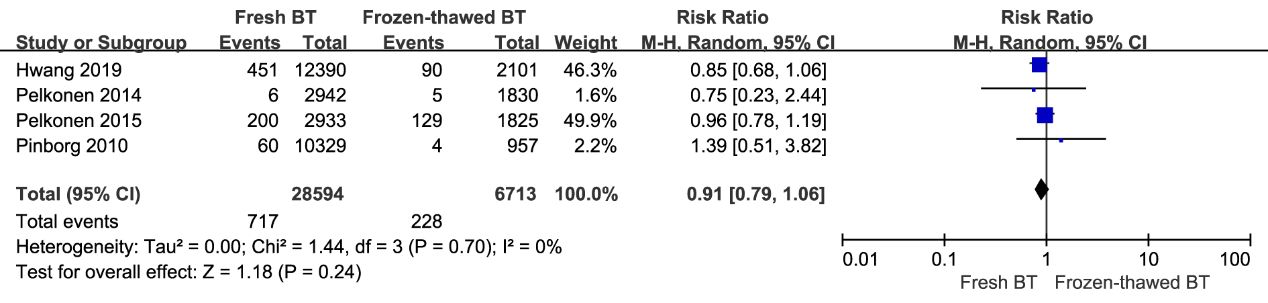


B
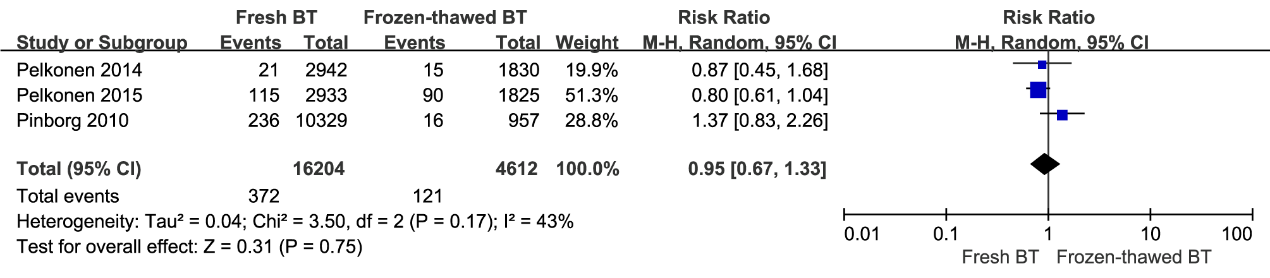


C
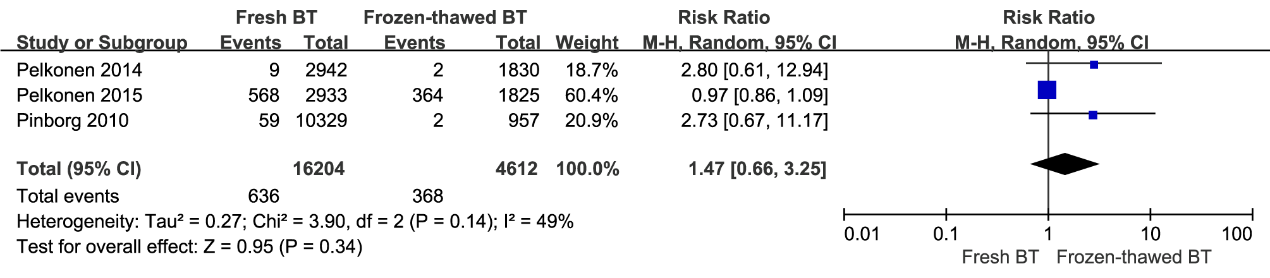


D


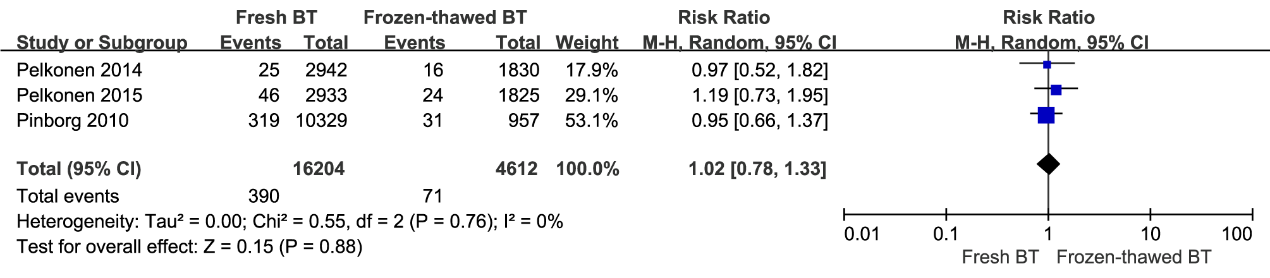


**Appendix 7**: Forest plot of comparison for (a) gastrointestinal system diseases,
(b) genitourinary system diseases, (c) eye, ear, face diseases and (d) musculoskeletal
diseases
