# Supplementary material for: Which is better for mothers and babies: fresh or frozen-thawed blastocyst transfer?
Source: BMC Pregnancy Childbirth. 2020 Sep 23;20:559. doi: 10.1186/s12884-020-03248-5 (PMC7513314; doi:10.1186/s12884-020-03248-5)
Supplement: Supplementary file 5 — Additional file 5: Appendices 8–18. Publication bias. [file 12884_2020_3248_MOESM5_ESM.docx]

Publication bias

A B

**Appendix 8**: (a) Funnel plot and (b) Eggers regression of **IR** (*P*= 0.687)

A B

**Appendix 9**: (a) Funnel plot and (b) Eggers regression of **PR** (*P*= 0.429)

A B

**Appendix 10**: (a) Funnel plot and (b) Eggers regression of **OPR** (*P*= 0.002)

A B

**Appendix 11**: (a) Funnel plot and (b) Eggers regression of **CPR** (*P*= 0.019)

A B

**Appendix 12**: (a) Funnel plot and (b) Eggers regression of **EPR** (*P*= 0.569)

A B ****

**Appendix 13**: (a) Funnel plot and (b) Eggers regression of **PIH &PE** (*P*= 0.755)

A B

**Appendix 14**: (a) Funnel plot and (b) Eggers regression of **GDM** (*P*=0.246)

A B

**Appendix 15**: (a) Funnel plot and (b) Eggers regression of **PTD** (*P*= 0.386)

A B

**Appendix 16**: (a) Funnel plot and (b) Eggers regression of **LGA** (*P*= 0.779)

A B

**Appendix 17**: (a) Funnel plot and (b) Eggers regression of **SGA** (*P*= 0.351)

A B

**Appendix 18**: (a) Funnel plot and (b) Eggers regression of **LBW** (*P*= 0.216)
