# Supplementary material for: Which is better for mothers and babies: fresh or frozen-thawed blastocyst transfer?
Source: BMC Pregnancy Childbirth. 2020 Sep 23;20:559. doi: 10.1186/s12884-020-03248-5 (PMC7513314; doi:10.1186/s12884-020-03248-5)
Supplement: Supplementary file 6 — Additional file 6: Appendix 19–34. Subgroup analysis. [file 12884_2020_3248_MOESM6_ESM.docx]

**Subgroup analysis**

A


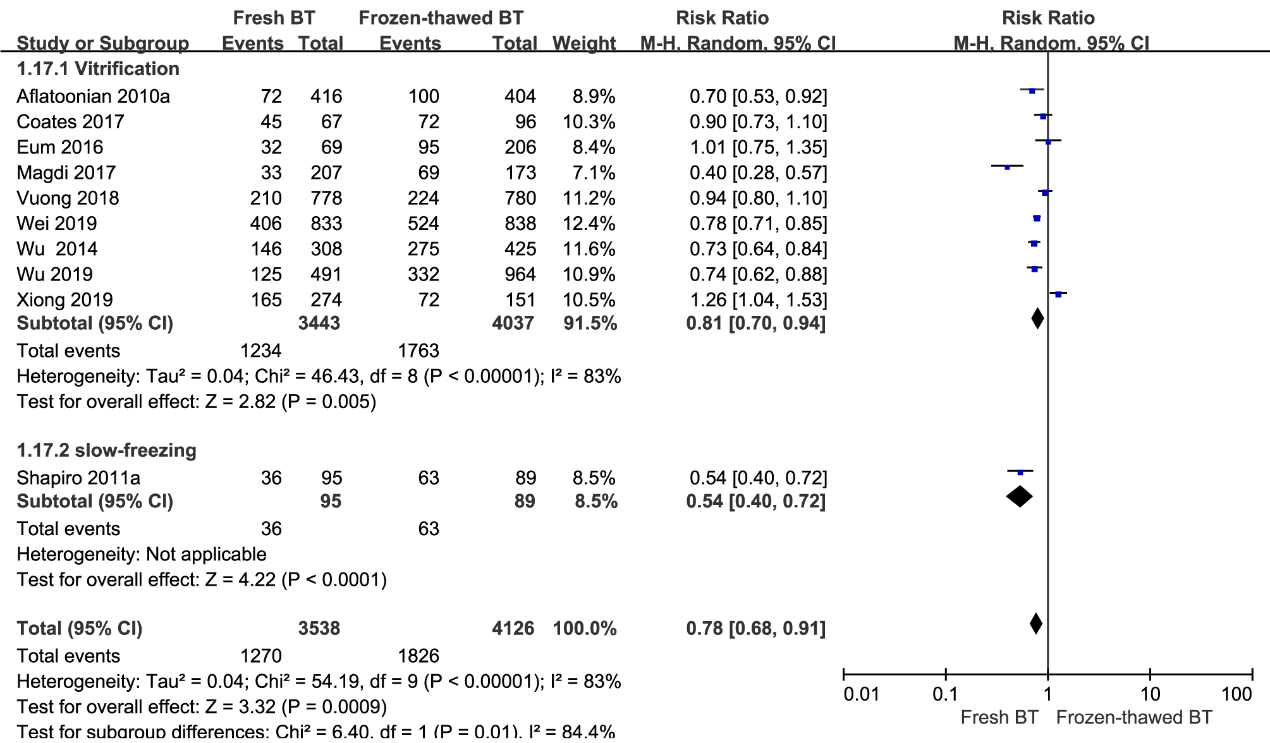


B


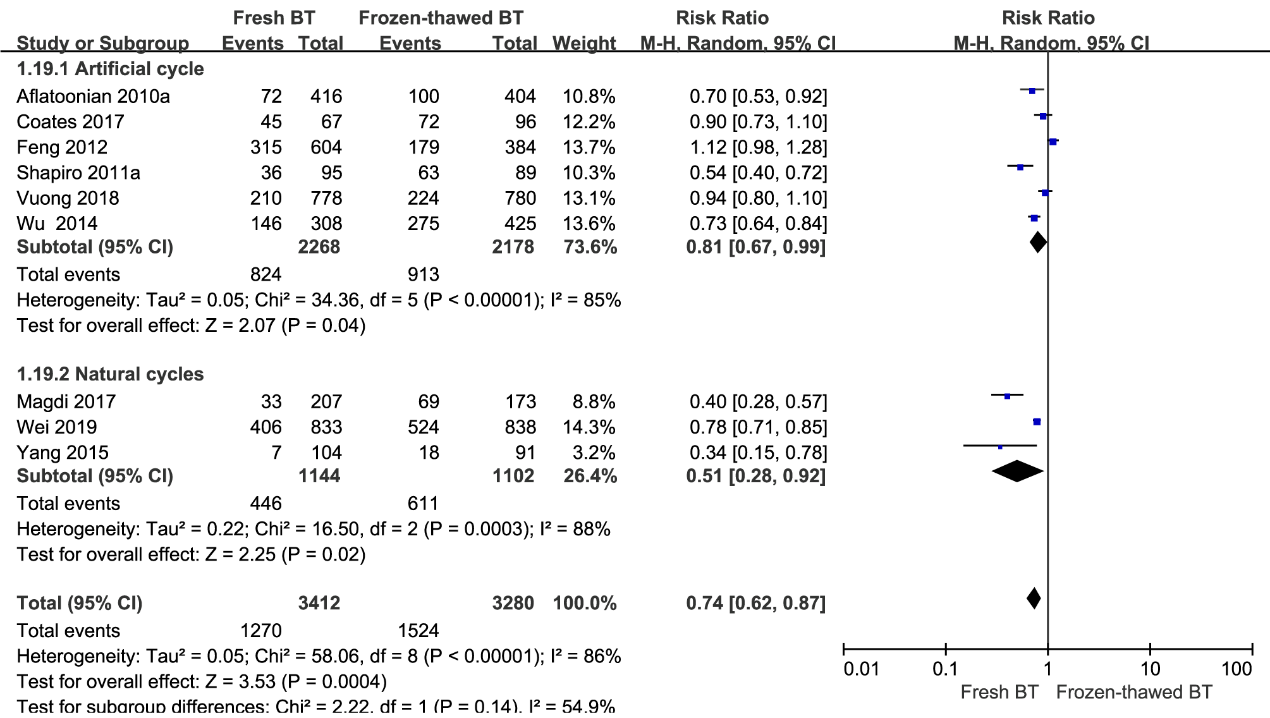


**Appendix 19**: Forest plot of comparison for subgroups of **implantation rate**: (a) the cryopreservation type: vitrification or slow-freezing and (b) endometrial preparation in fresh BT: the artificial cycle or the natural

A


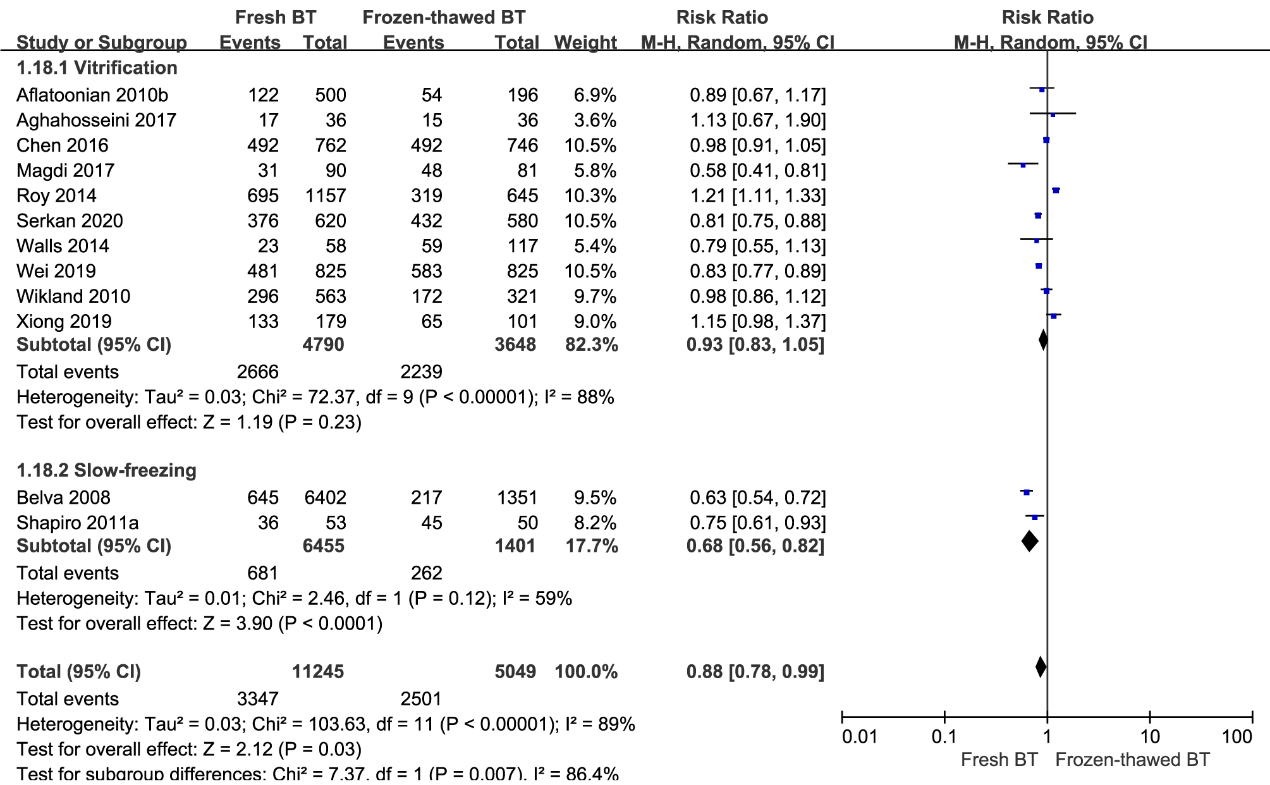


B
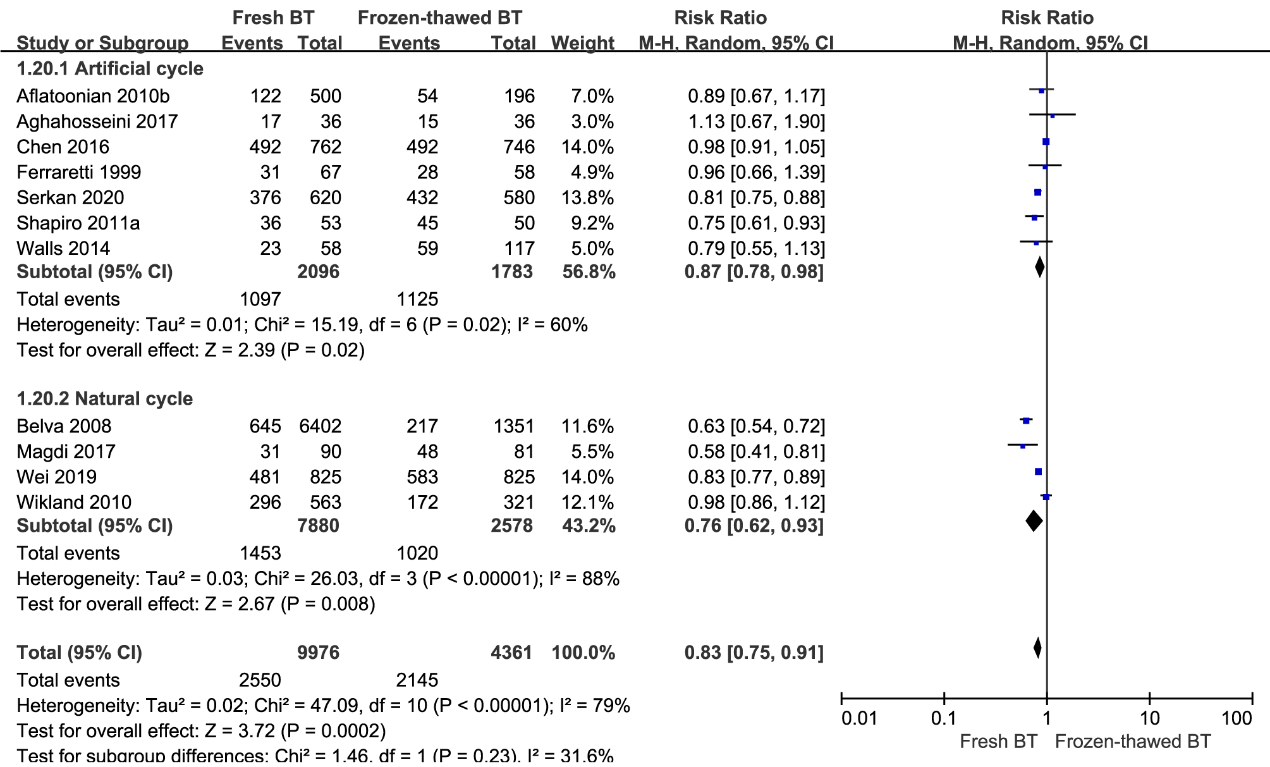


**Appendix 20**: Forest plot of comparison for subgroups of **pregnancy rate**: (a) the cryopreservation type: vitrification or slow-freezing and (b) endometrial preparation in fresh BT: the artificial cycle or the natural cycle

A


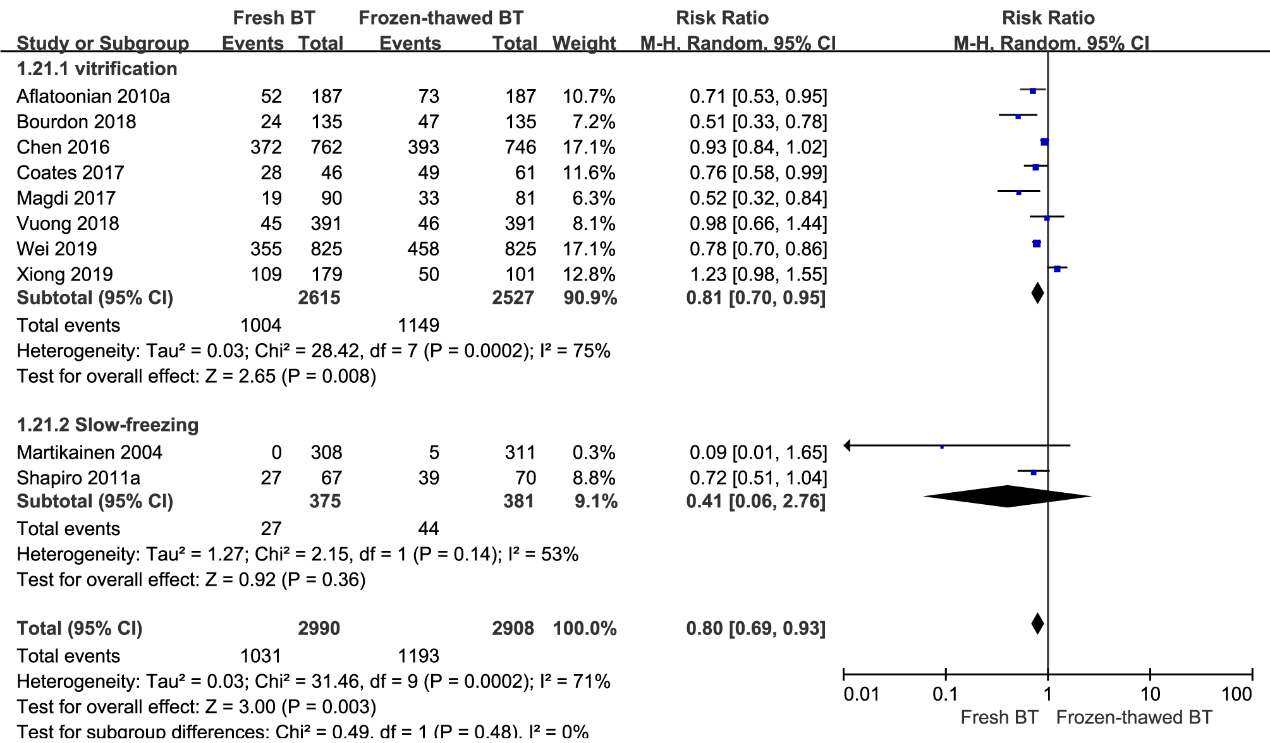


B


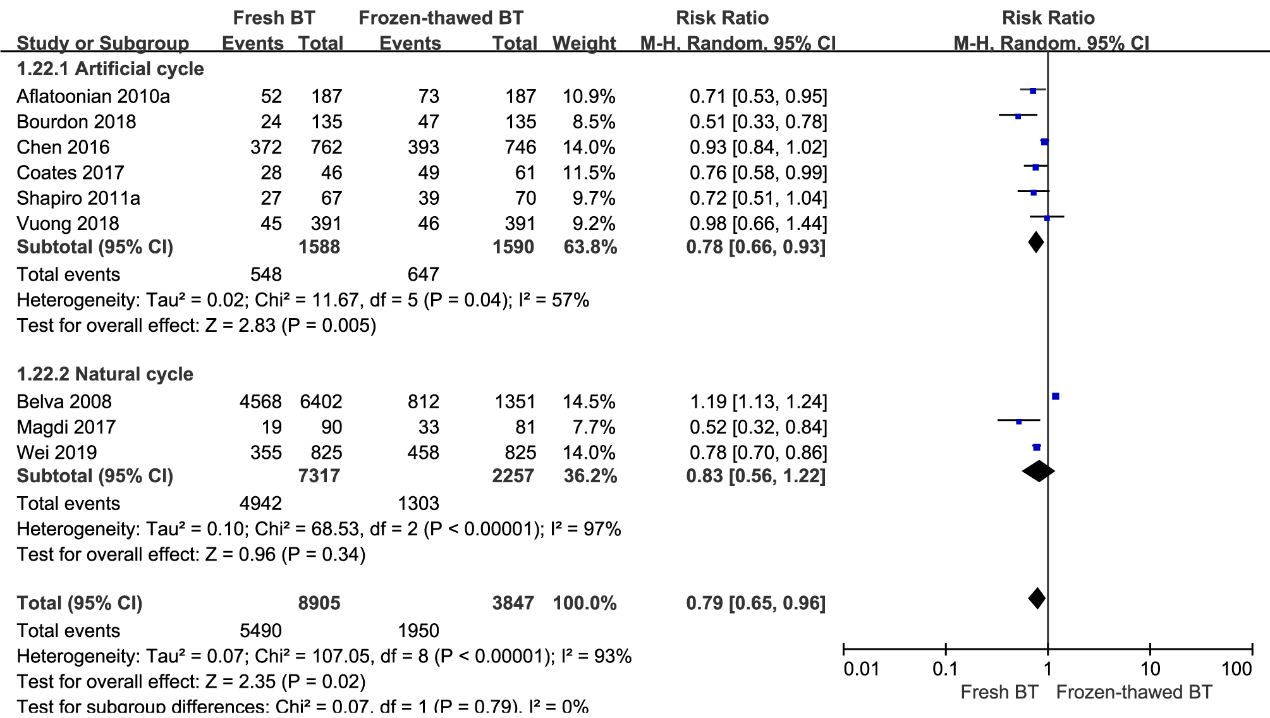


**Appendix 21**: Forest plot of comparison for subgroups of **ongoing pregnancy rate**: (a) the cryopreservation type: vitrification or slow-freezing and (b) endometrial preparation in fresh BT: the artificial cycle or the natural cycle

A


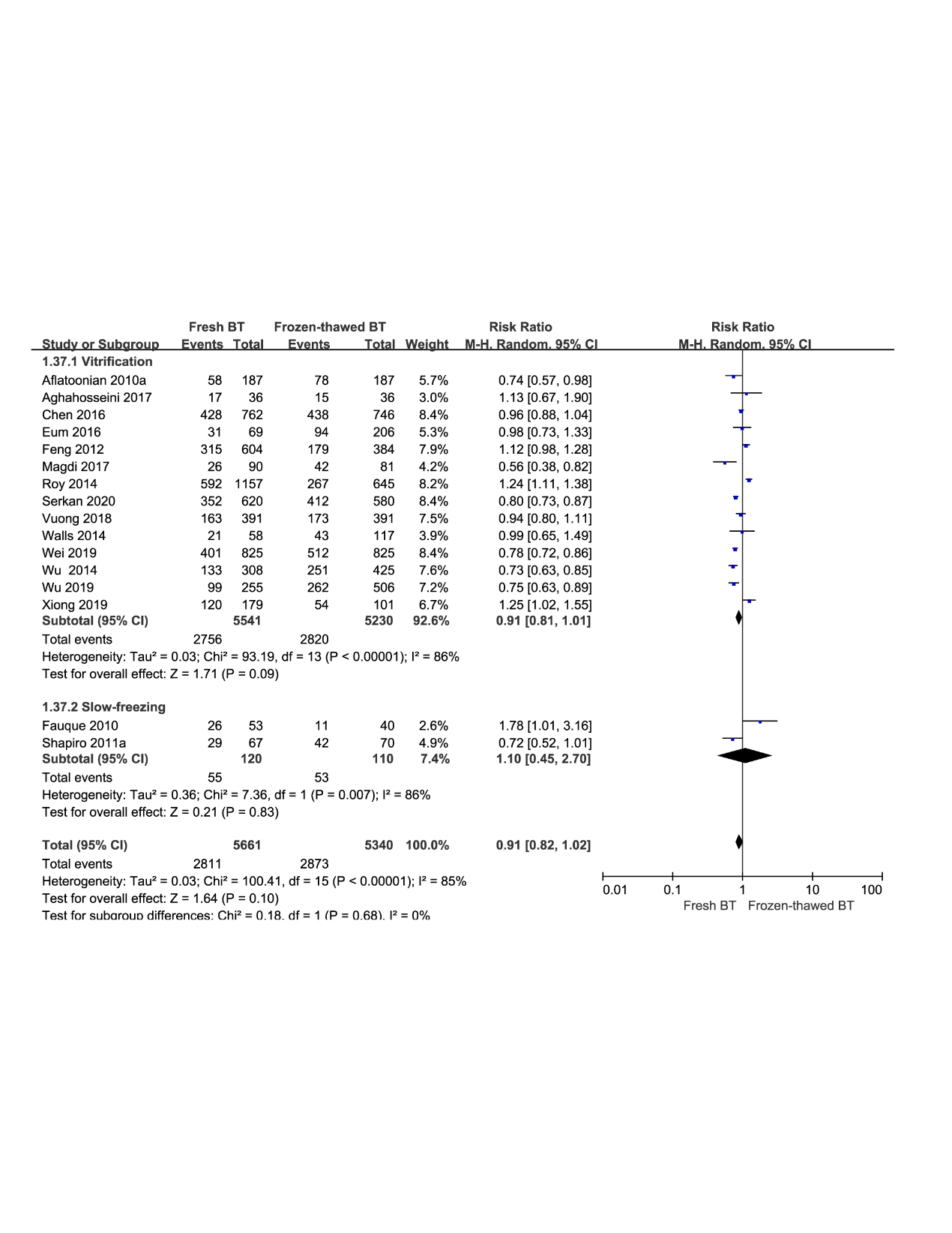


B


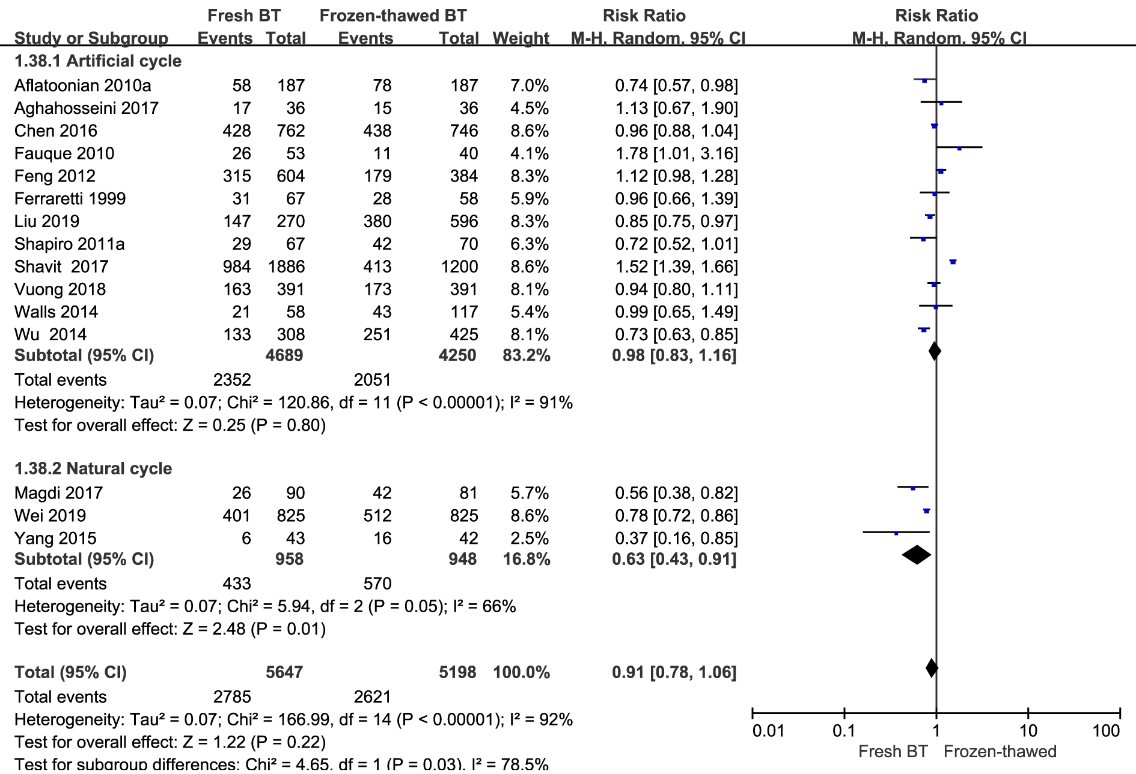


**Appendix 22**: Forest plot of comparison for subgroups of **clinical pregnancy rate:** (a) the cryopreservation type: vitrification or slow-freezing and (b) endometrial preparation in fresh BT: the artificial cycle or the natural cycle

A
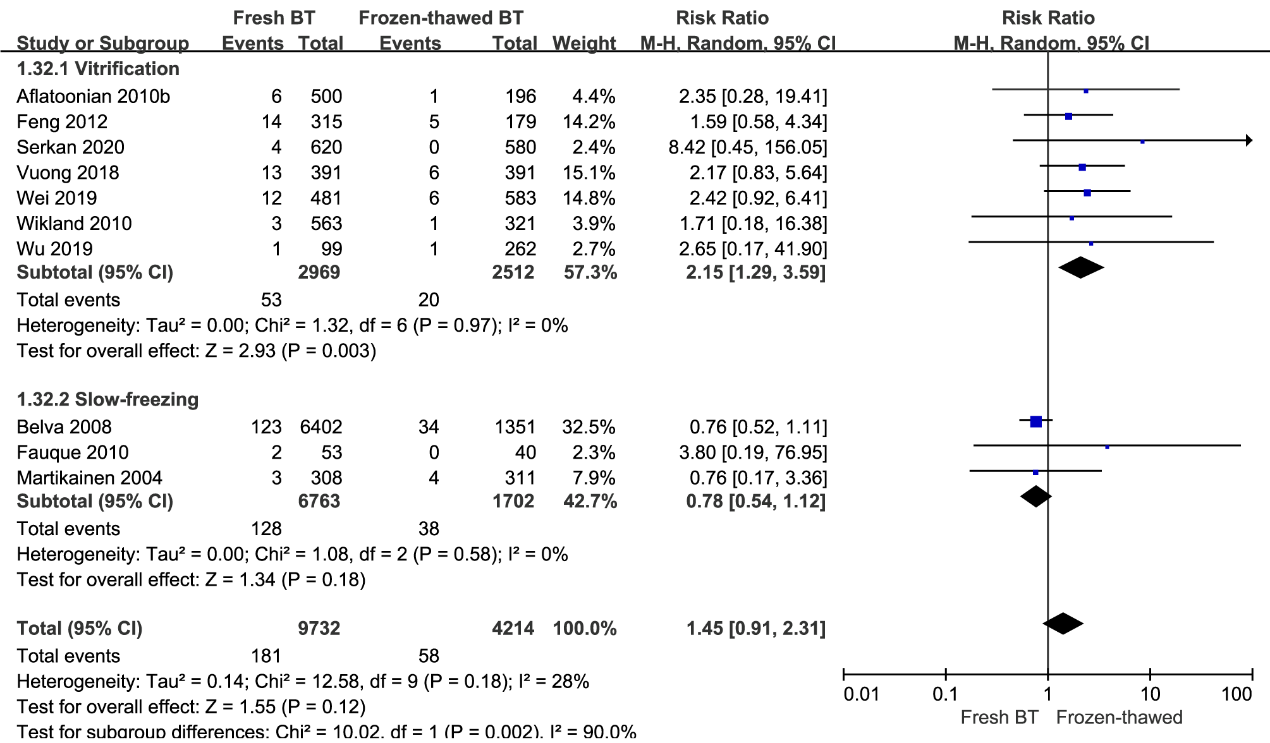


B
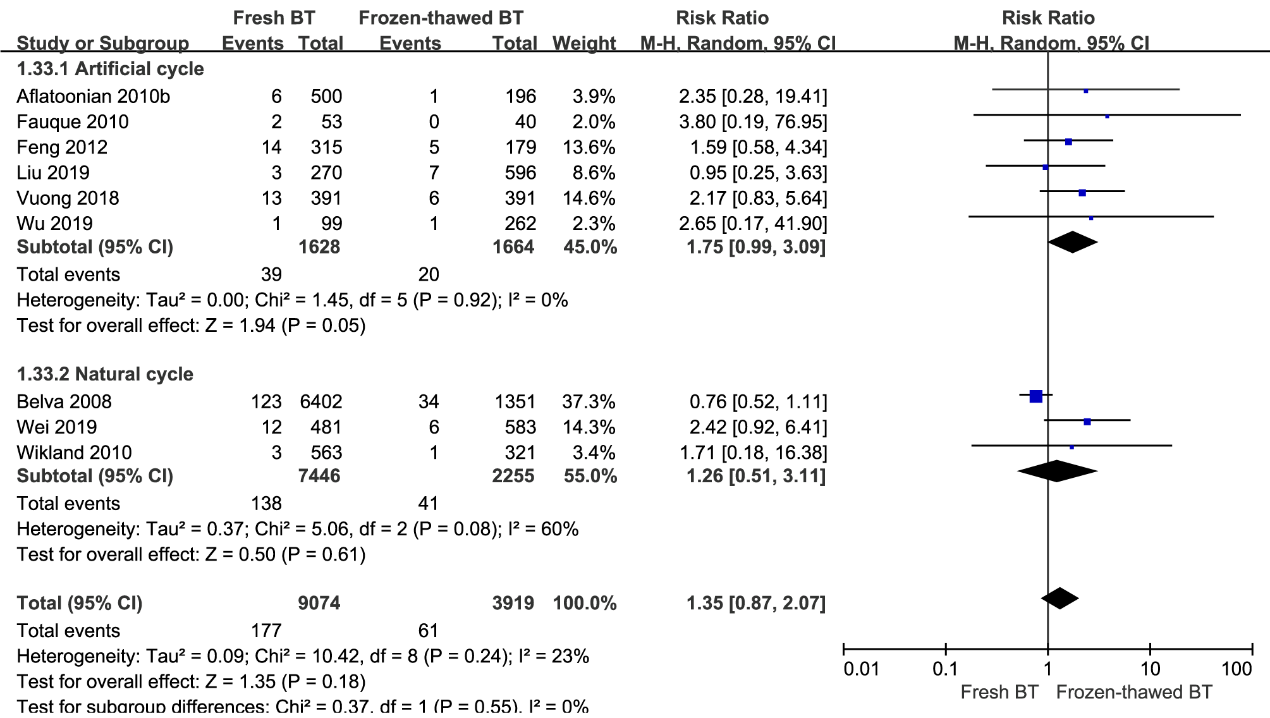


**Appendix 23**: Forest plot of comparison for subgroups of **ectopic pregnancy rate:** (a) the cryopreservation type: vitrification or slow-freezing and (b) endometrial preparation in fresh BT: the artificial cycle or the natural cycle

A


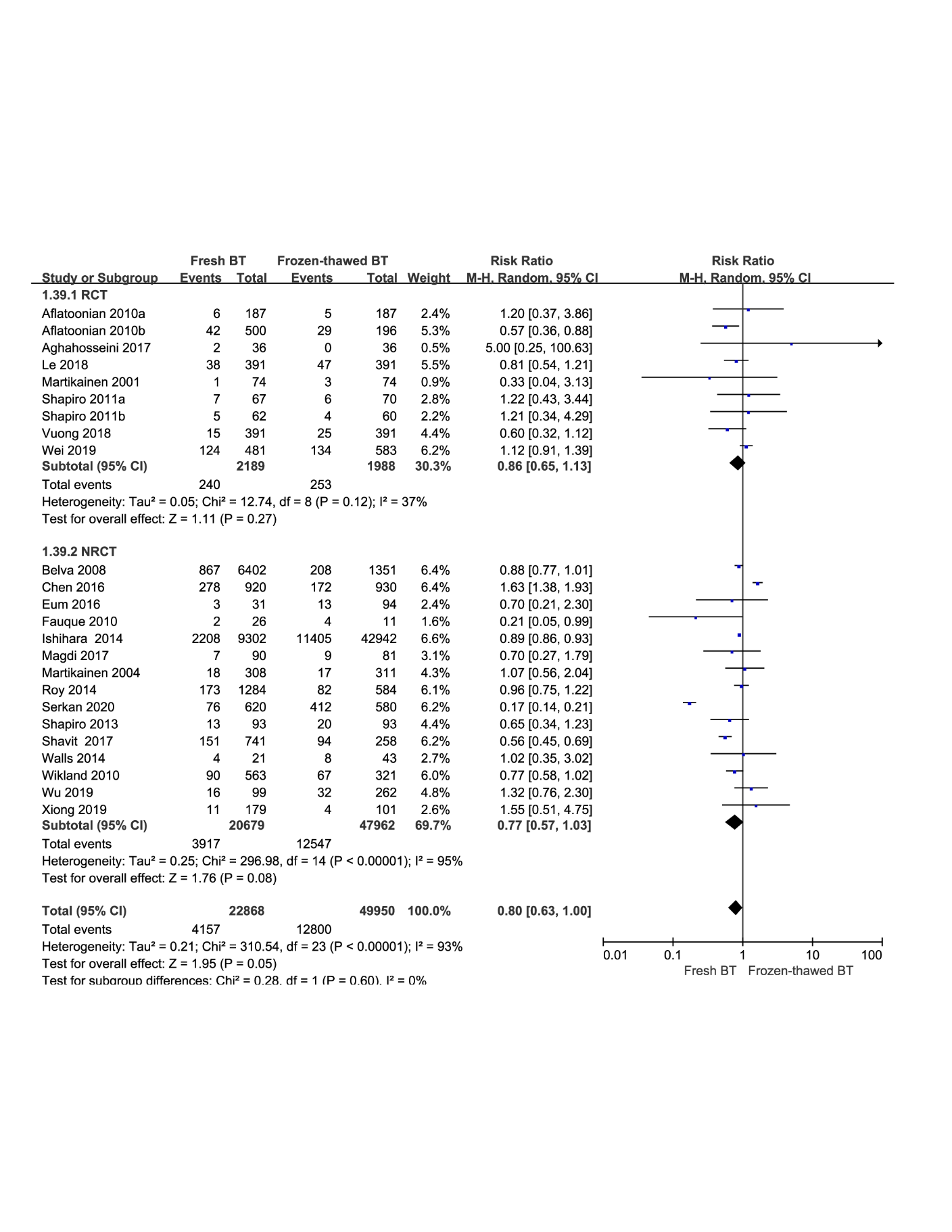


B


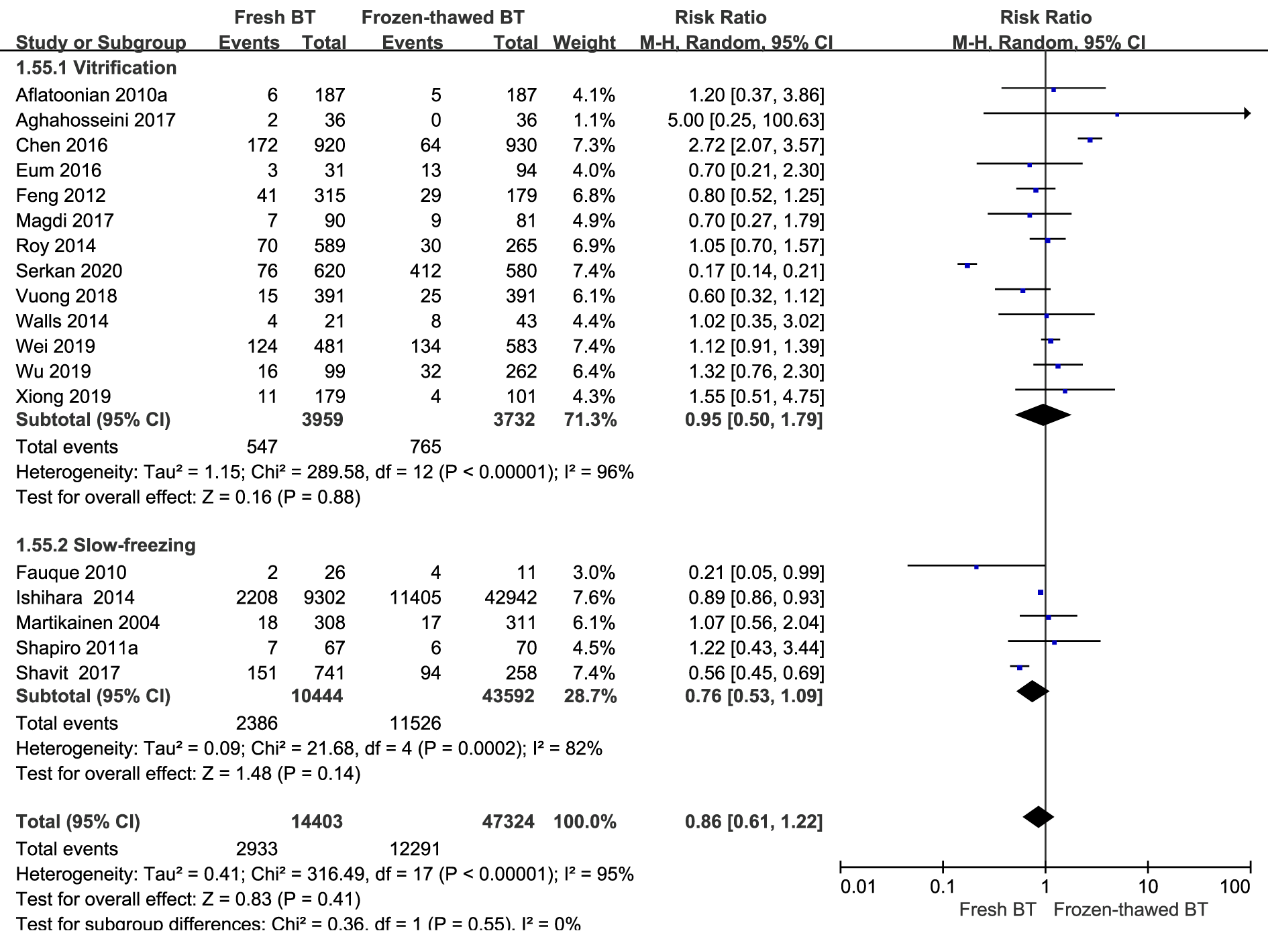


**Appendix 24**: Forest plot of comparison for subgroups of **micarriage rate**: (a) RCT or NRCT and (b) the cryopreservation type: vitrification or slow-freezing

**
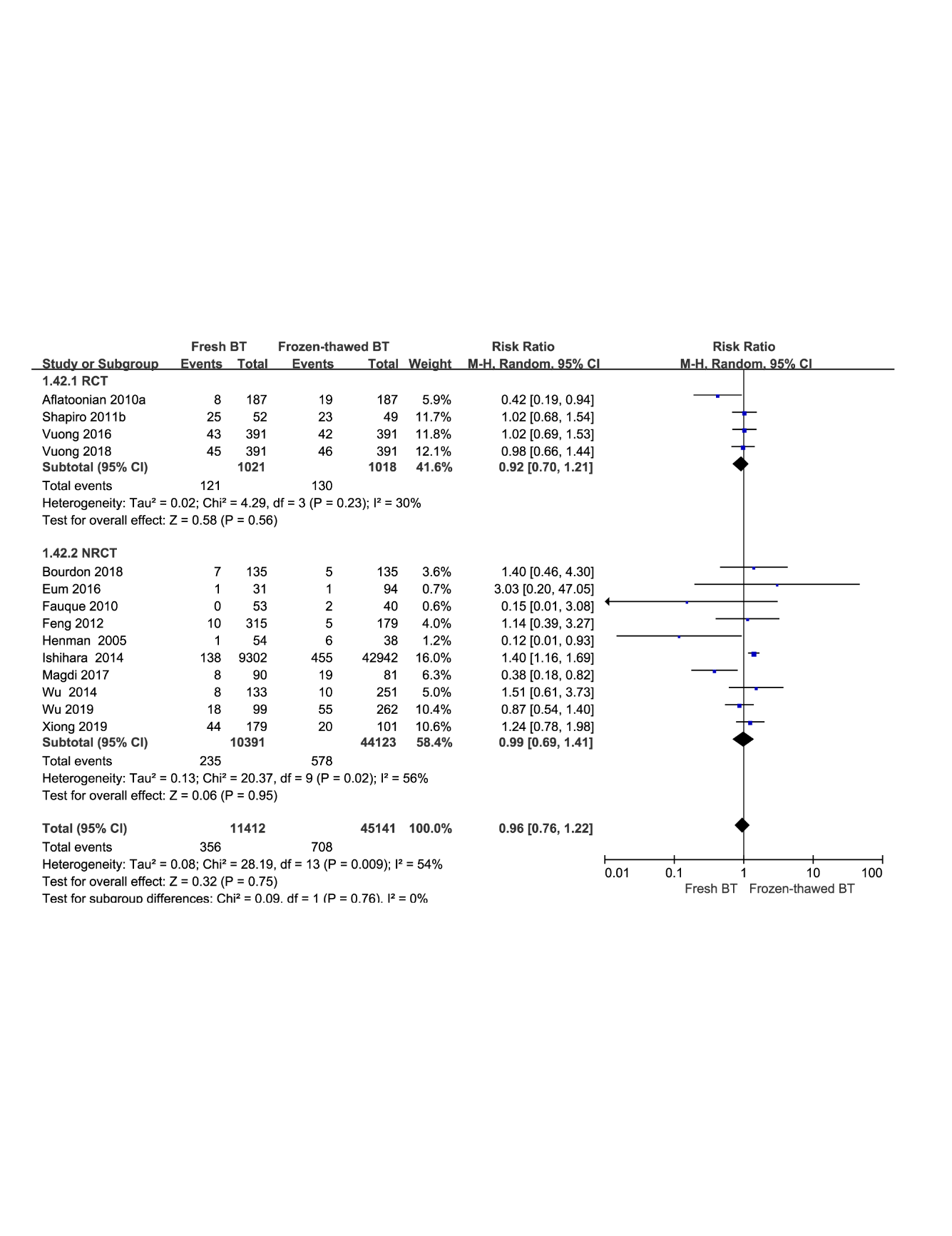
**

**Appendix 25**: Forest plot of comparison for subgroups of **multiple pregnancy rate**: RCT or NRCT

A


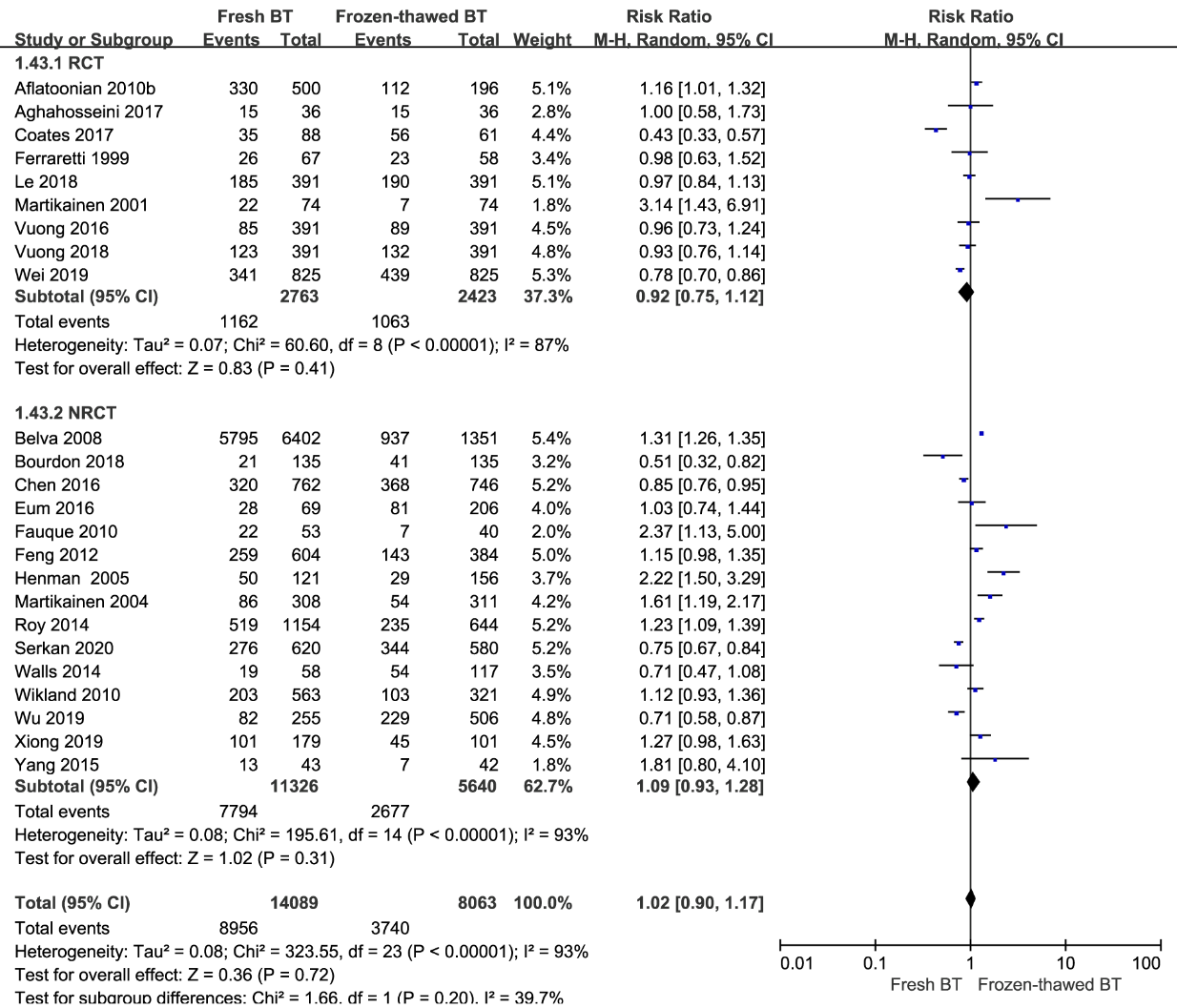


B

**
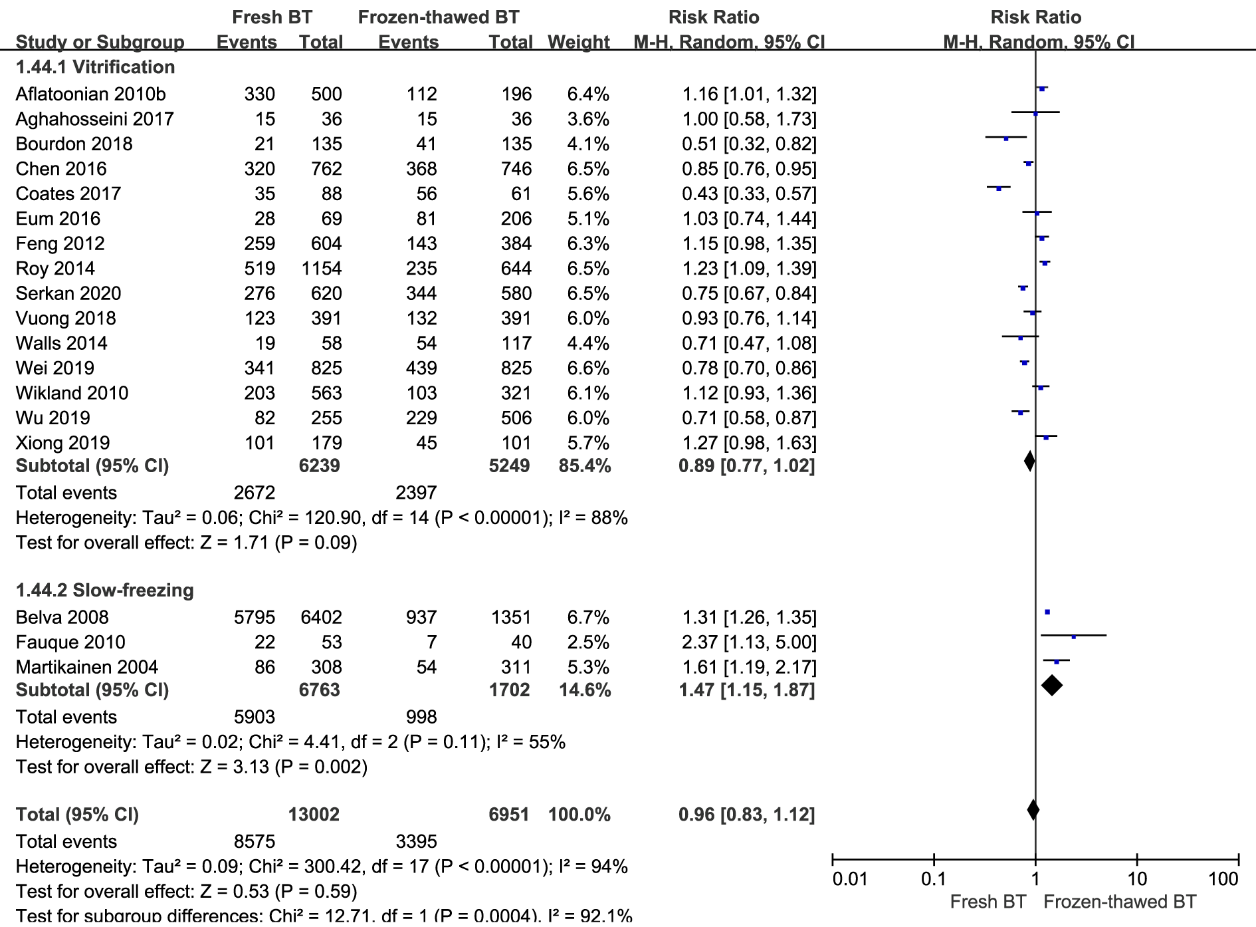
**

C

**
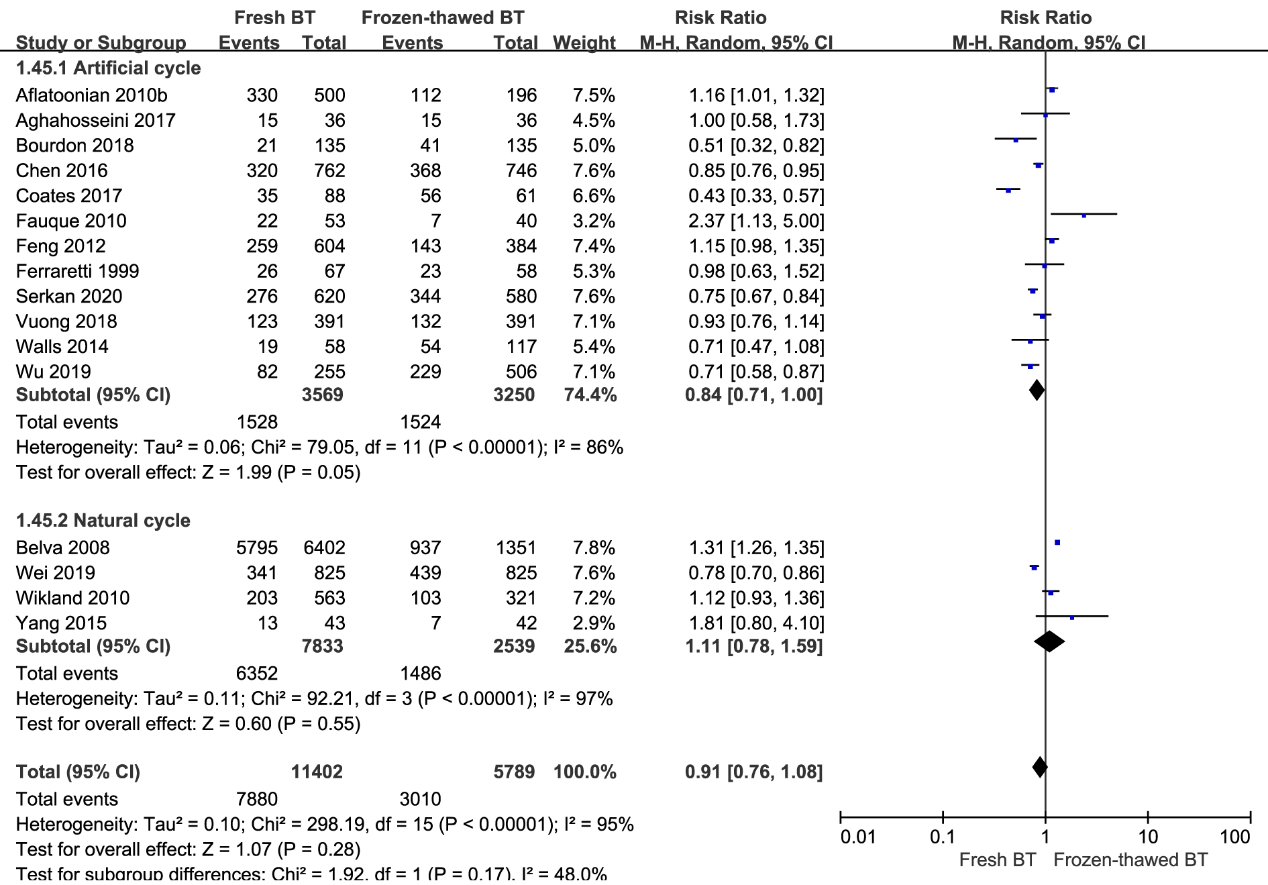
**

**Appendix 26**: Forest plot of comparison for subgroups of **live birth rate**: (a) RCT or NRCT, (b) the cryopreservation type: vitrification or slow-freezing and (c) endometrial preparation in fresh BT: the artificial cycle or the natural cycle

A


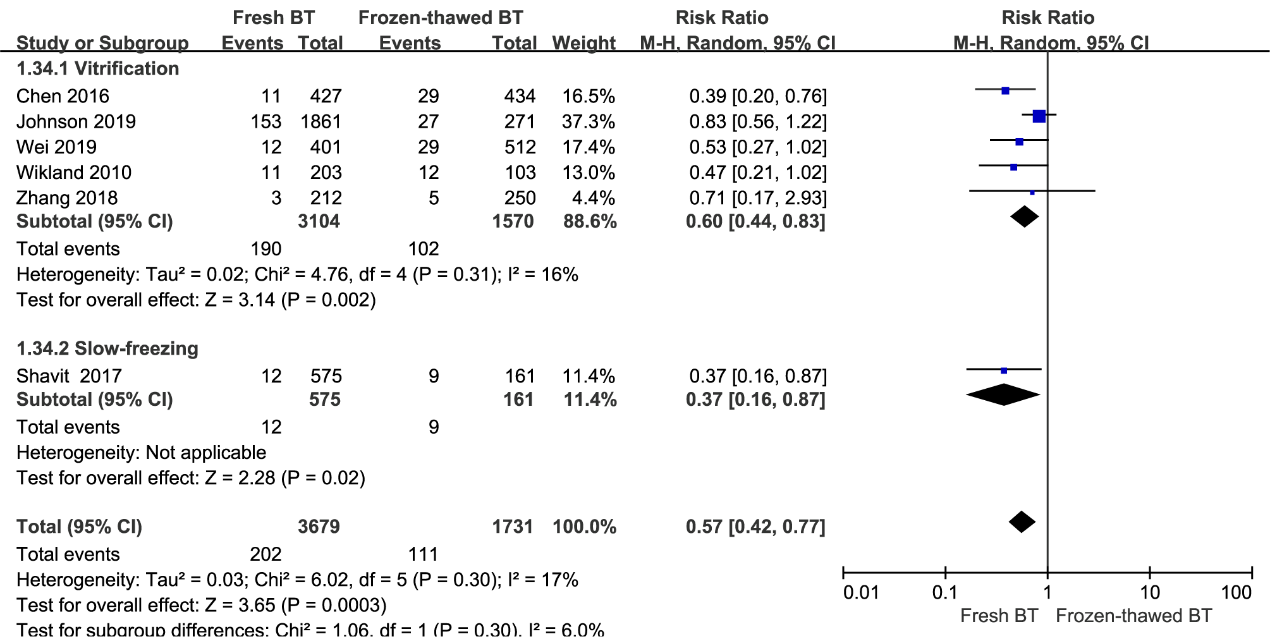


B
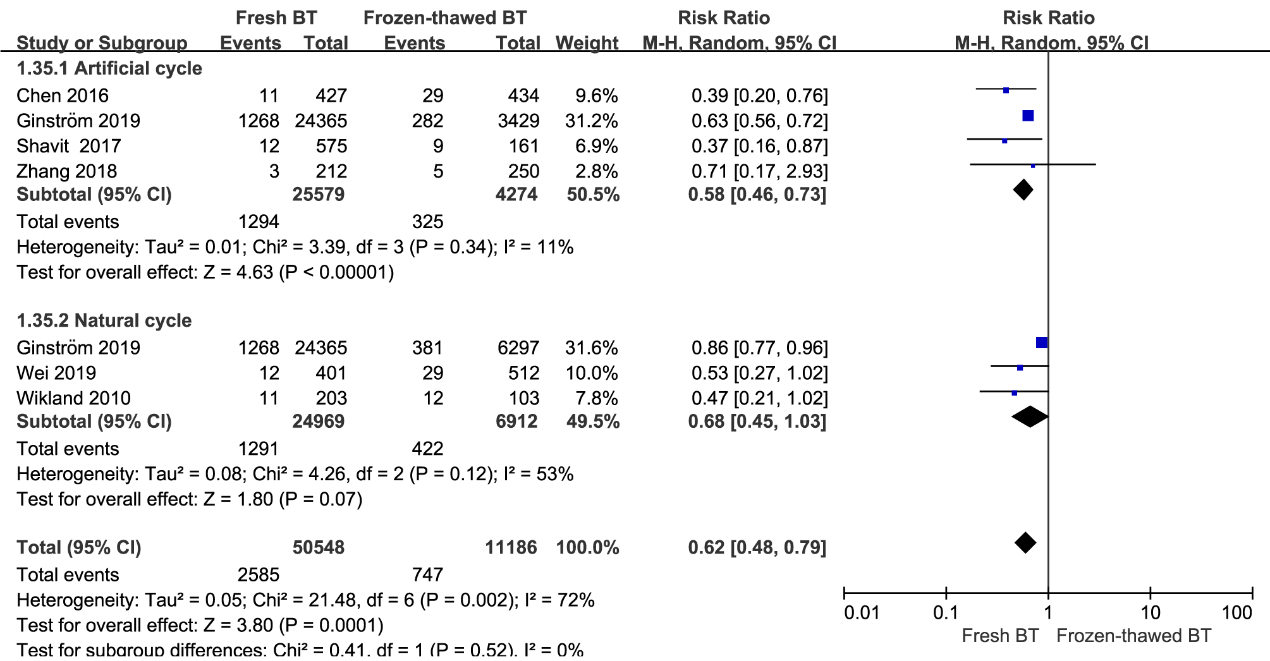


**Appendix 27**: Forest plot of comparison for subgroups of **PIH and pre-eclampsia**: (a) the cryopreservation type: vitrification or slow-freezing and (b) endometrial preparation in fresh BT: the artificial cycle or the natural cycle


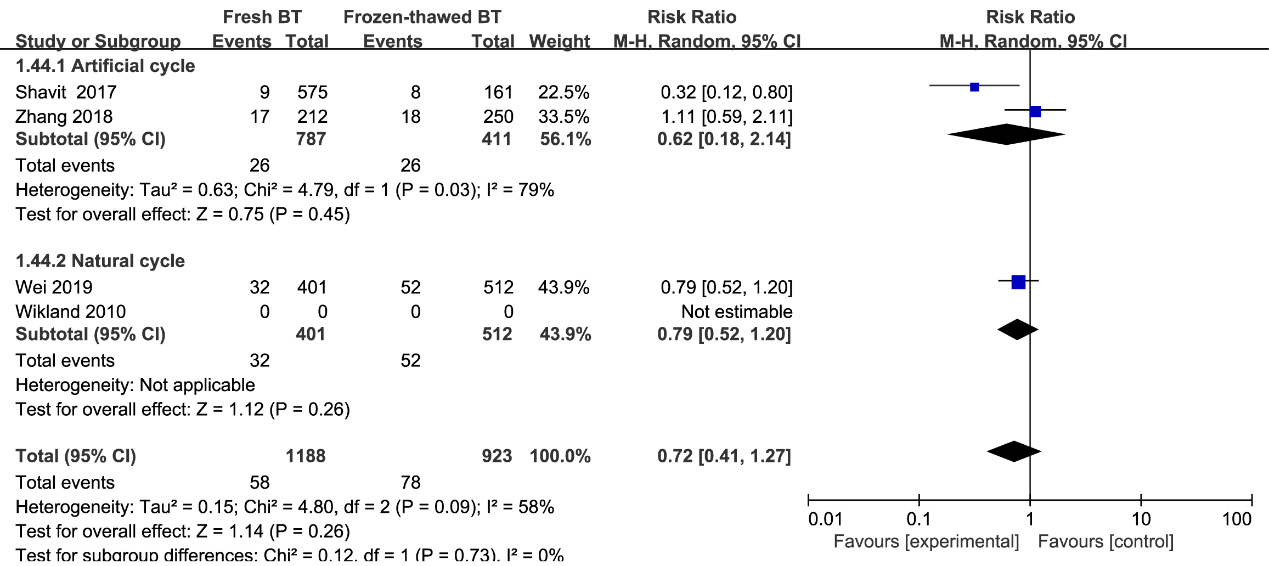


**Appendix 28**: Forest plot of comparison for subgroups of **GDM**: endometrial preparation in fresh BT: the artificial cycle or the natural cycle

A
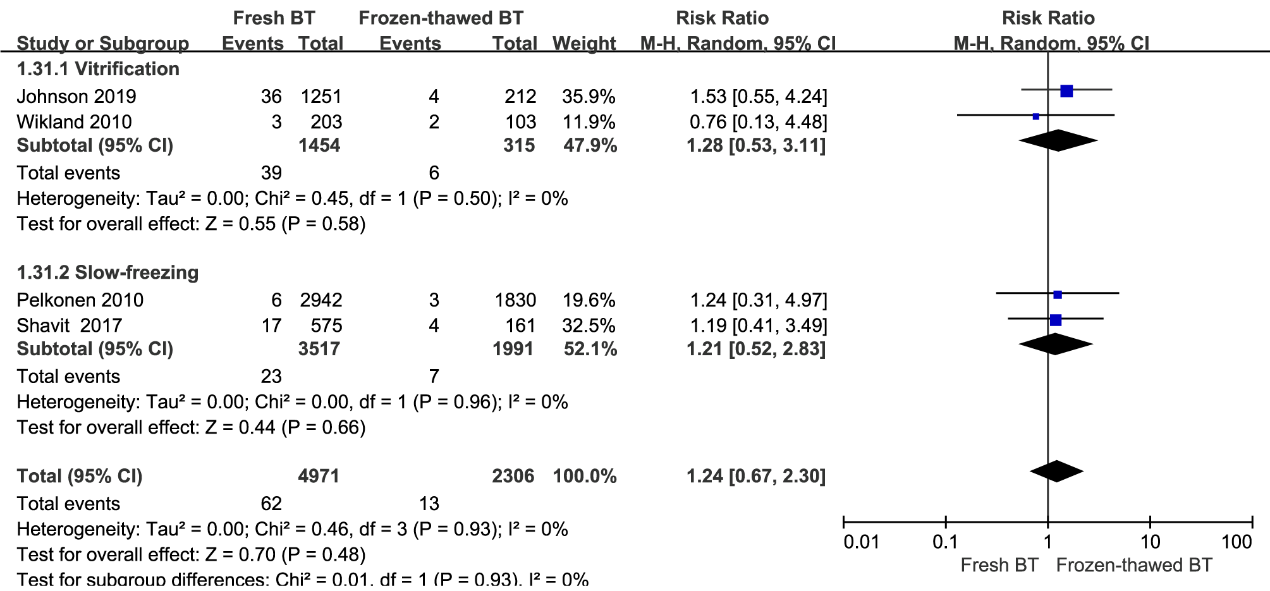


B
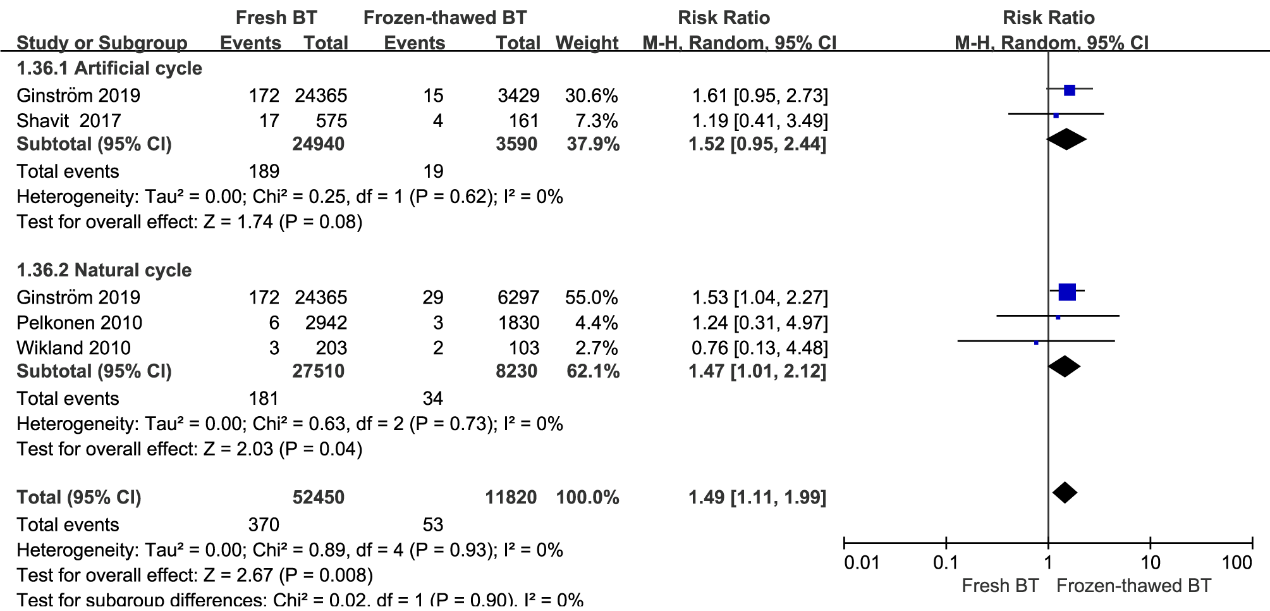


**Appendix 29**: Forest plot of comparison for subgroups of **placental abruption**: (a) the cryopreservation type: vitrification or slow-freezing and (b) endometrial preparation in fresh BT: the artificial cycle or the natural cycle

A


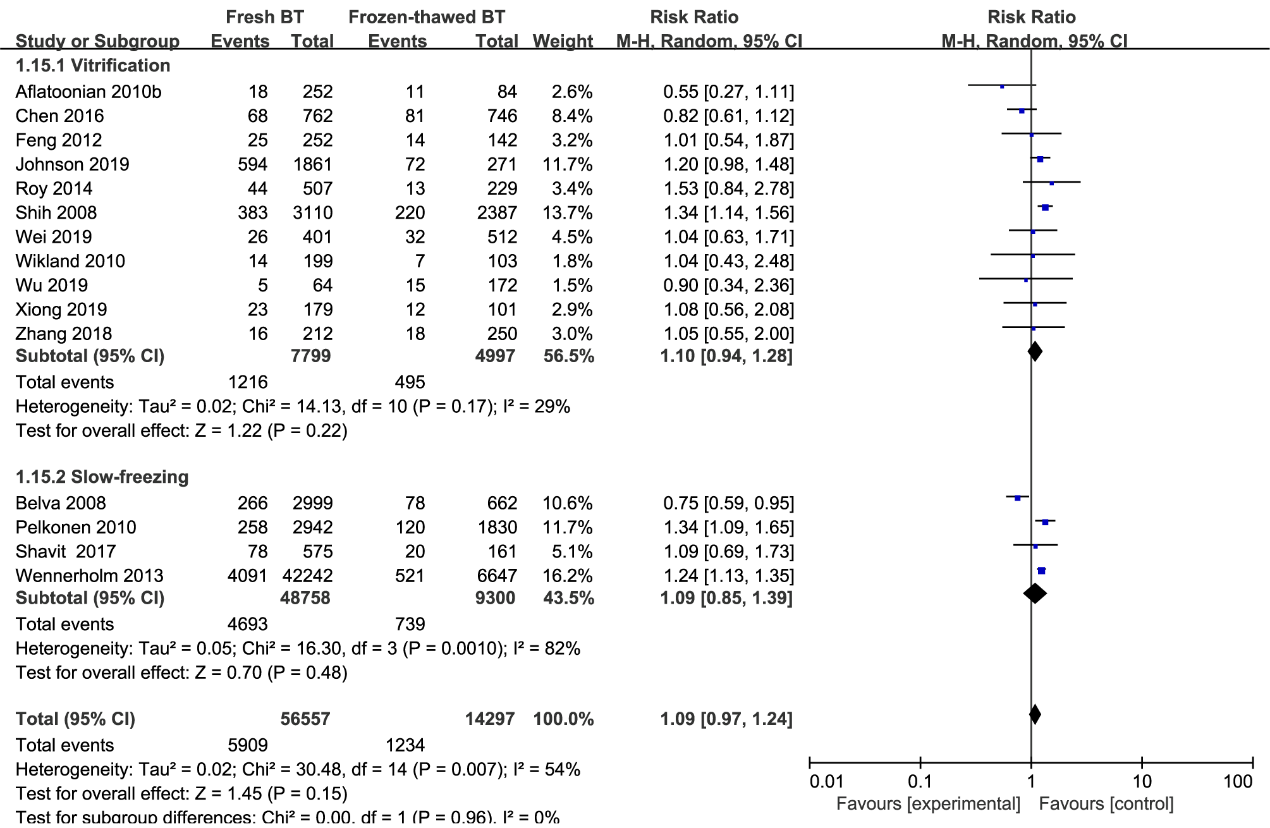


B
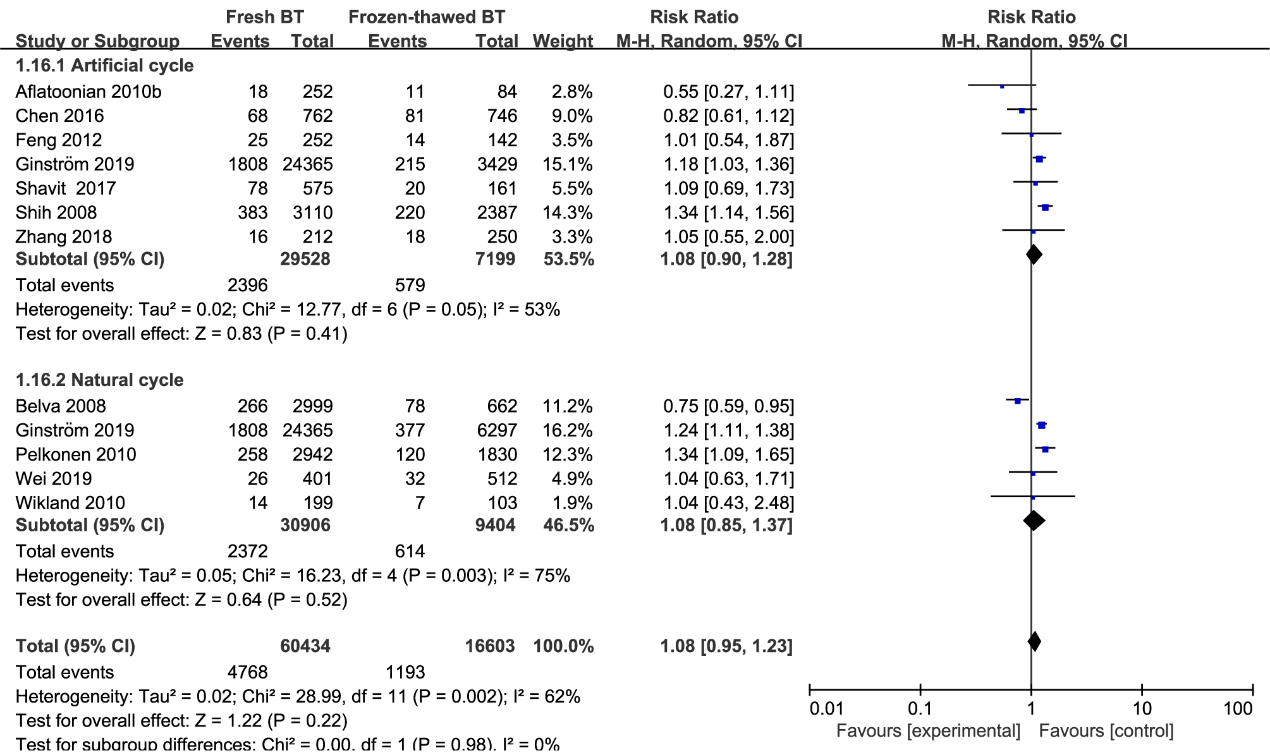


**Appendix 30**: Forest plot of comparison for subgroups of **PTD**: endometrial preparation in fresh BT: (A) the cryopreservation type: vitrification or slow-freezing (B) endometrial preparation in fresh BT: the artificial cycle or the natural cycle

A


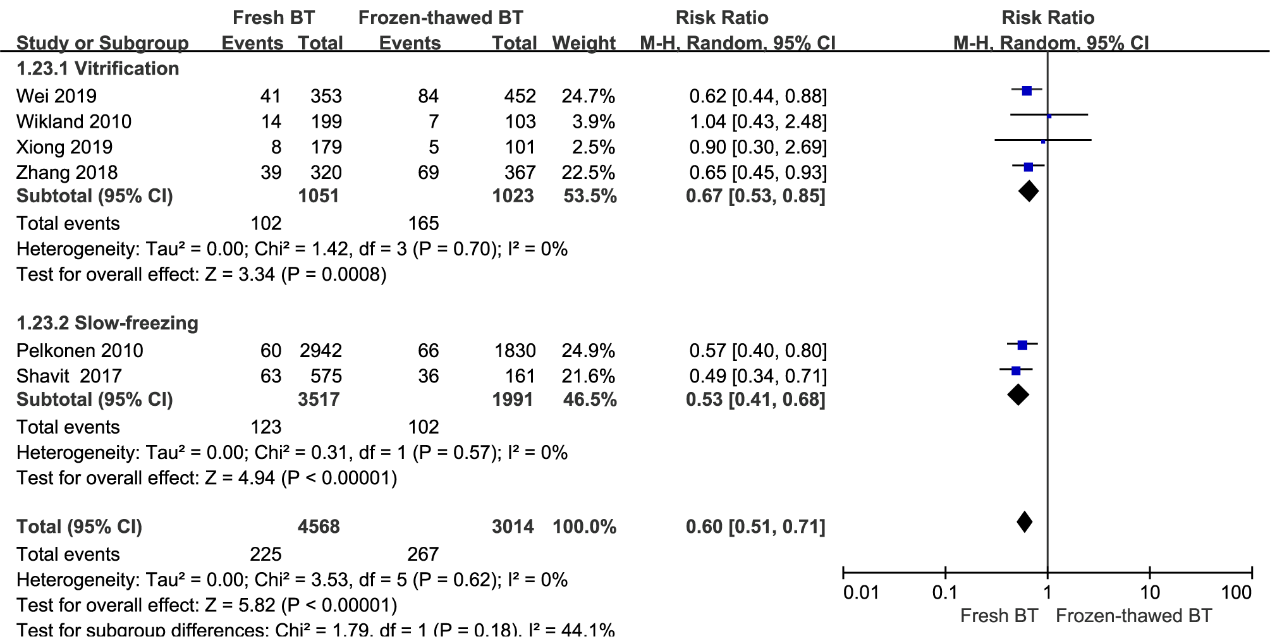


B
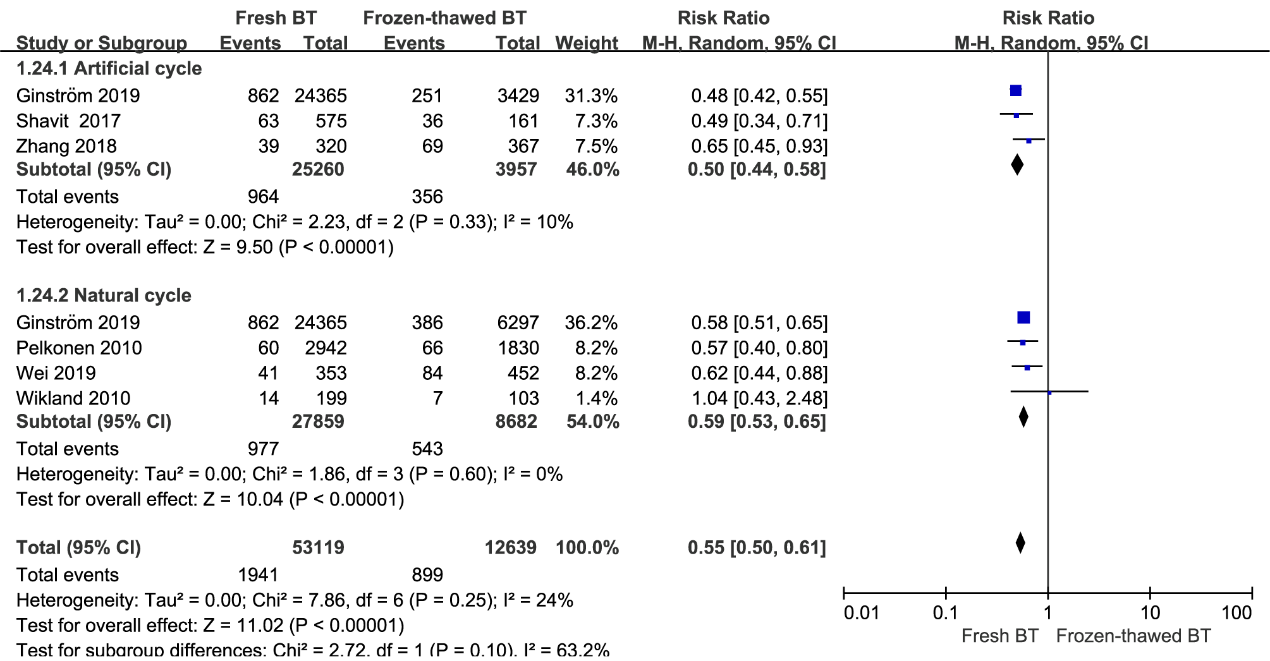


**Appendix 31**: Forest plot of comparison for subgroups of **LGA** (a) the cryopreservation type: vitrification or slow-freezing and (b) endometrial preparation in fresh BT: the artificial cycle or the natural cycle

A


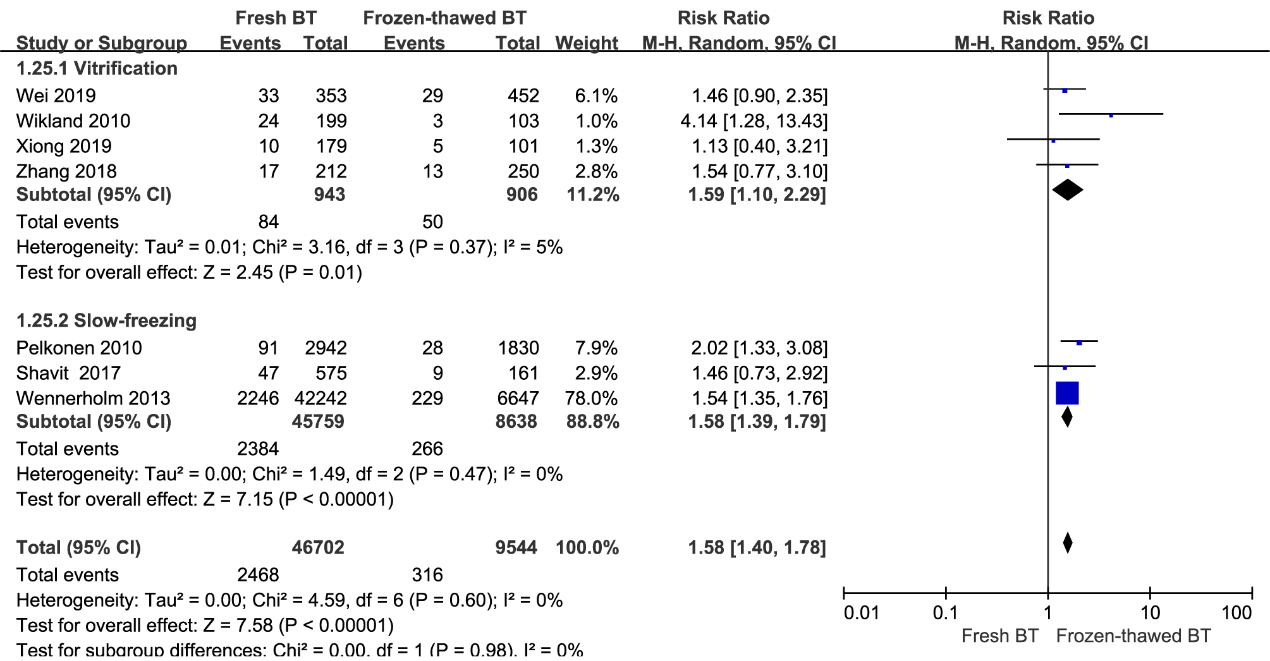
B
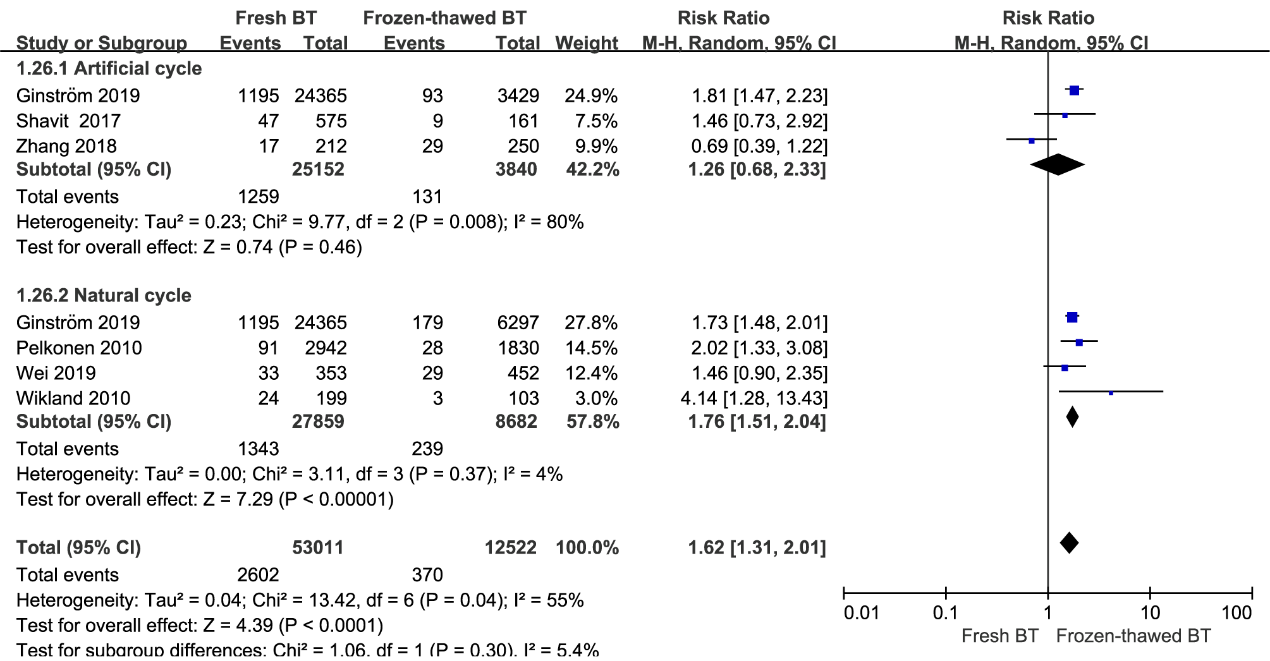


**Appendix 32**: Forest plot of comparison for subgroups of **SGA** (a) the cryopreservation type: vitrification or slow-freezing and (b) endometrial preparation in fresh BT: the artificial cycle or the natural cycle

A
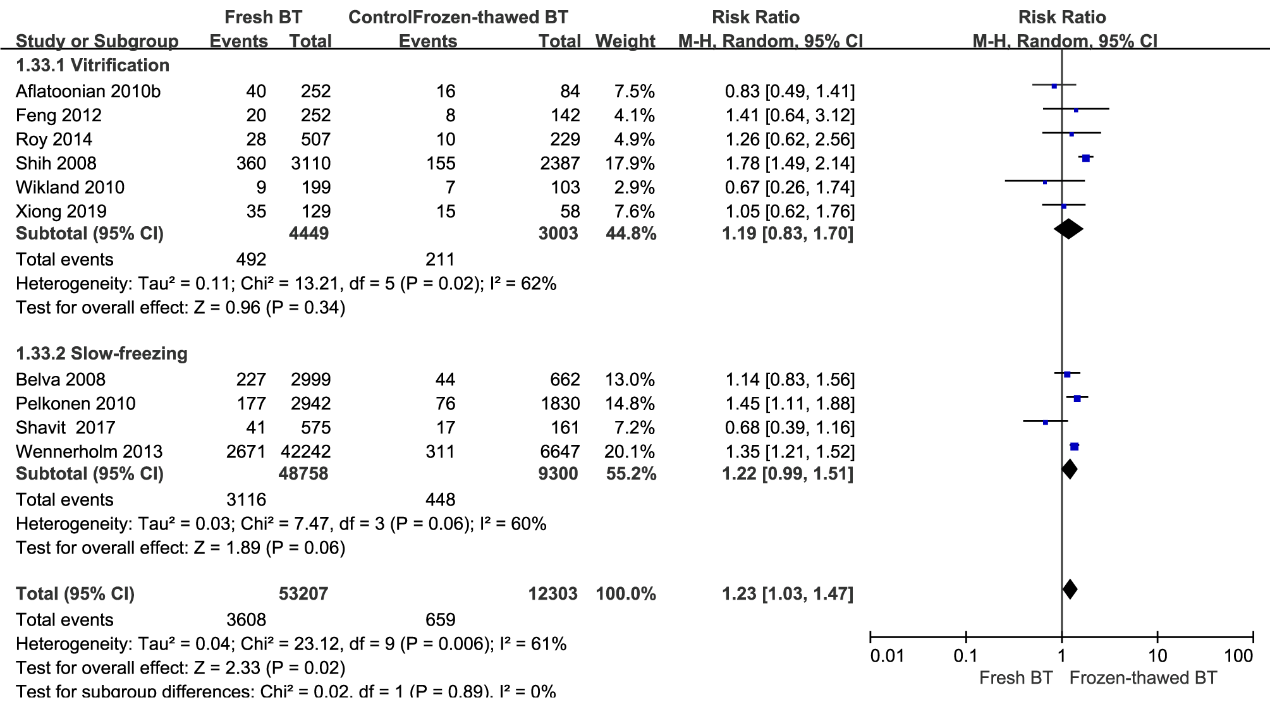


B
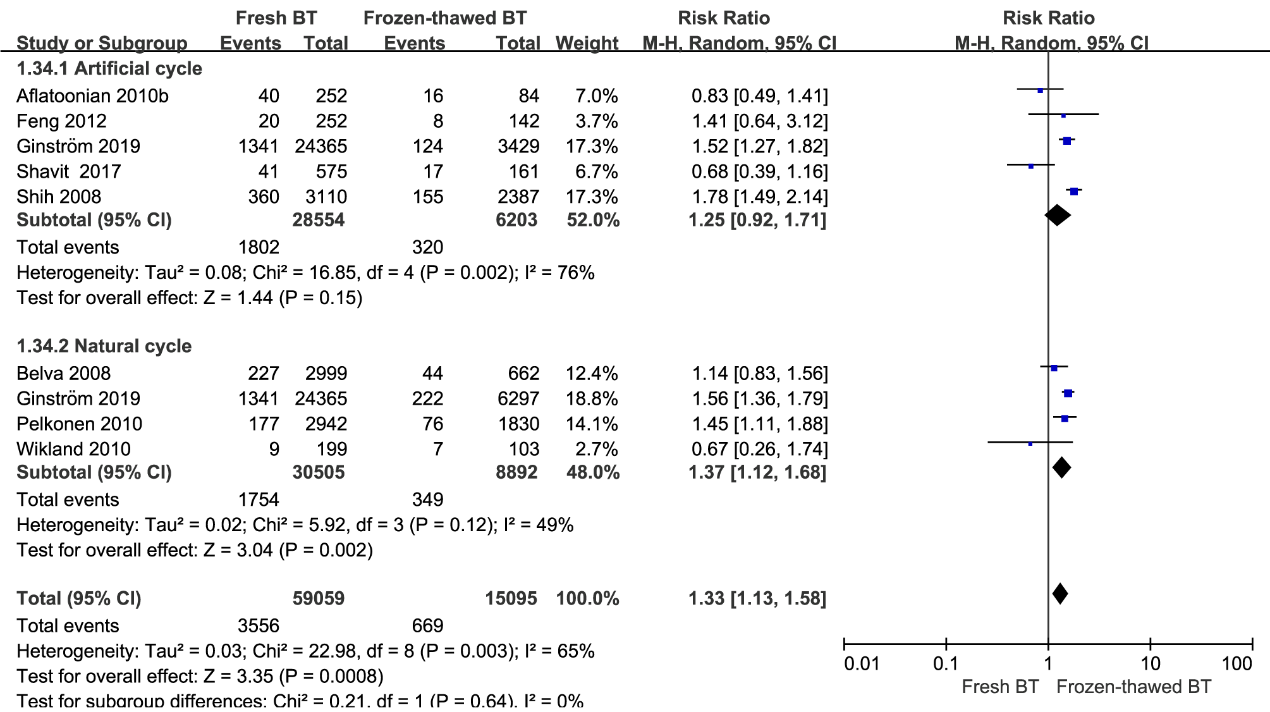


**Appendix 33**: Forest plot of comparison for subgroups of **LBW** (a) the cryopreservation type: vitrification or slow-freezing and (b) endometrial preparation in fresh BT: the artificial cycle or the natural cycle


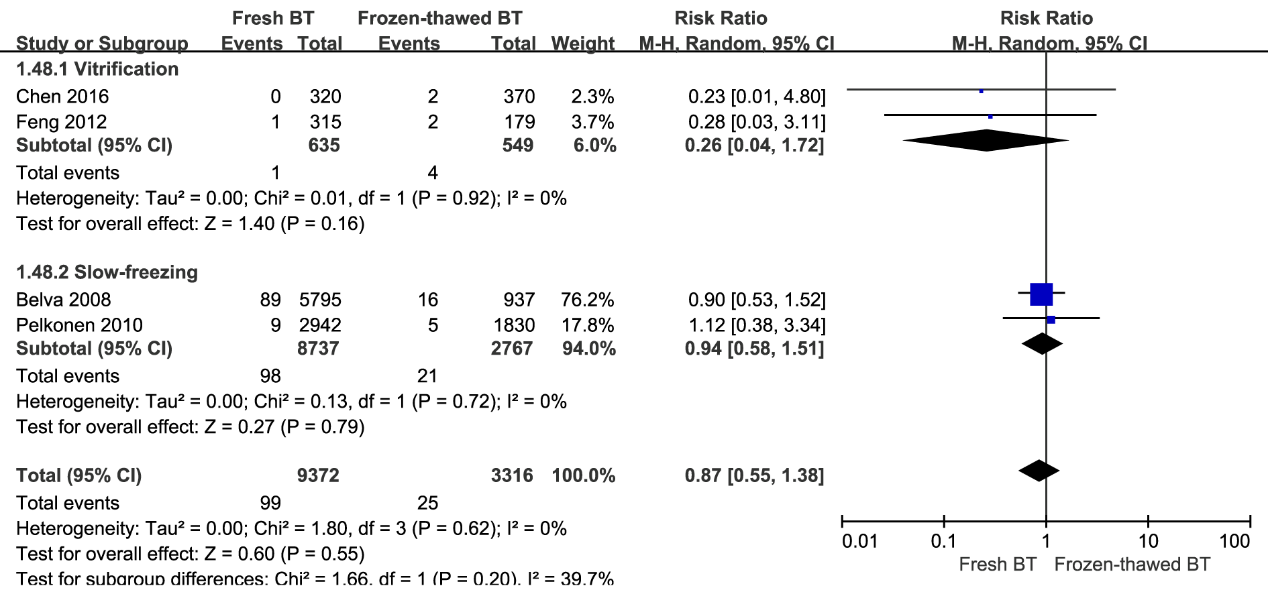


**Appendix 34**: Forest plot of comparison for subgroups of **stillbirth rate**: the cryopreservation type: vitrification or slow-freezing
