# Supplementary material for: Which is better for mothers and babies: fresh or frozen-thawed blastocyst transfer?
Source: BMC Pregnancy Childbirth. 2020 Sep 23;20:559. doi: 10.1186/s12884-020-03248-5 (PMC7513314; doi:10.1186/s12884-020-03248-5)
Supplement: Supplementary file 7 — Additional file 7: Appendix 35. Women with high ovarian response. [file 12884_2020_3248_MOESM7_ESM.docx]

Women with high ovarian response

A
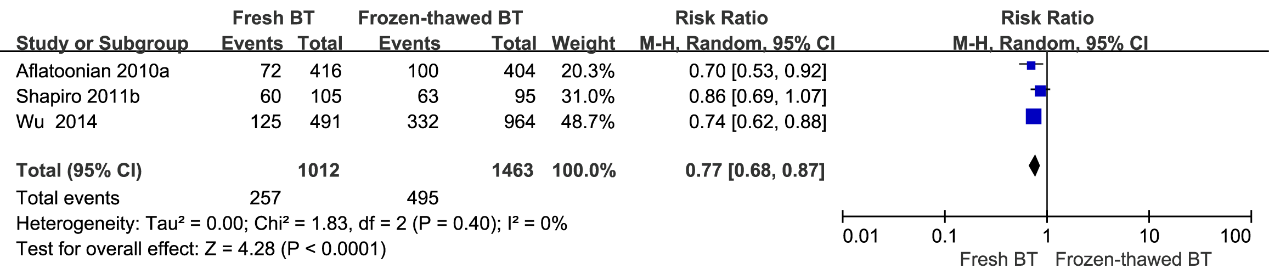


B
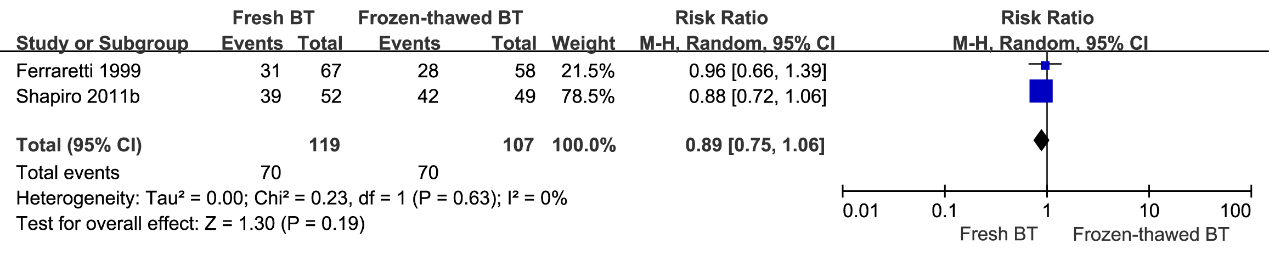


C
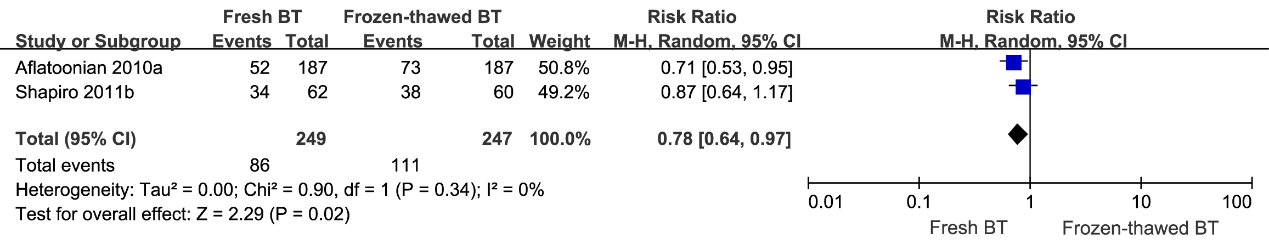


D


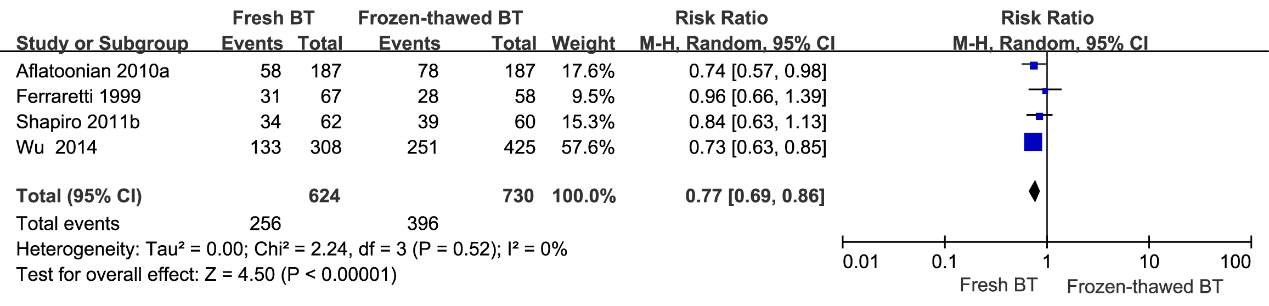


**Appendix 35**: Forest plot of comparison for women with high ovarian response: (a) implantation rate, (b) pregnancy rate, (c) ongoing pregnancy rate and (d) clinical pregnancy rate
