# Supplementary material for: Which is better for mothers and babies: fresh or frozen-thawed blastocyst transfer?
Source: BMC Pregnancy Childbirth. 2020 Sep 23;20:559. doi: 10.1186/s12884-020-03248-5 (PMC7513314; doi:10.1186/s12884-020-03248-5)
Supplement: Supplementary file 8 — Additional file 8: Appendix 36. Single blastocyst transfer. [file 12884_2020_3248_MOESM8_ESM.docx]

Single blastocyst transfer

A
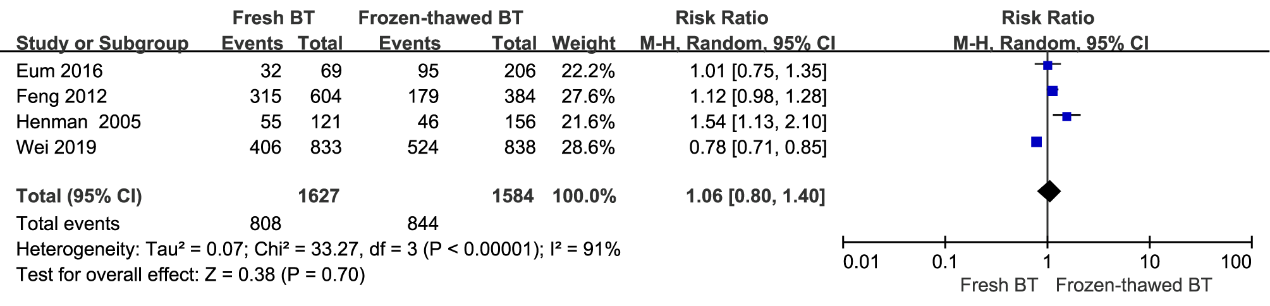


B


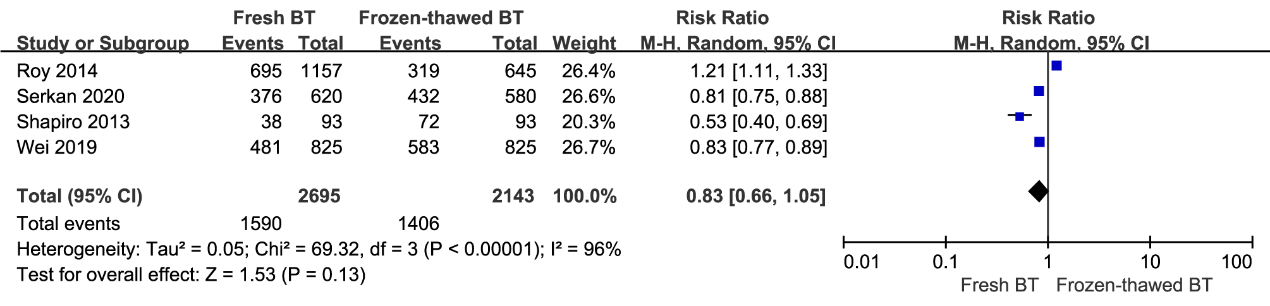


C


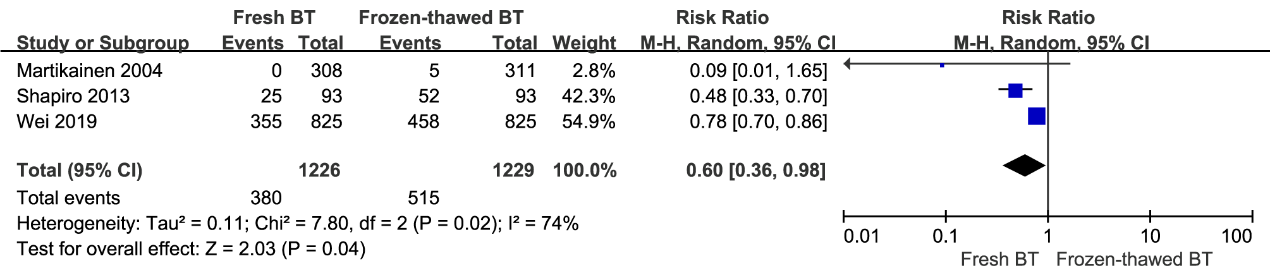


D
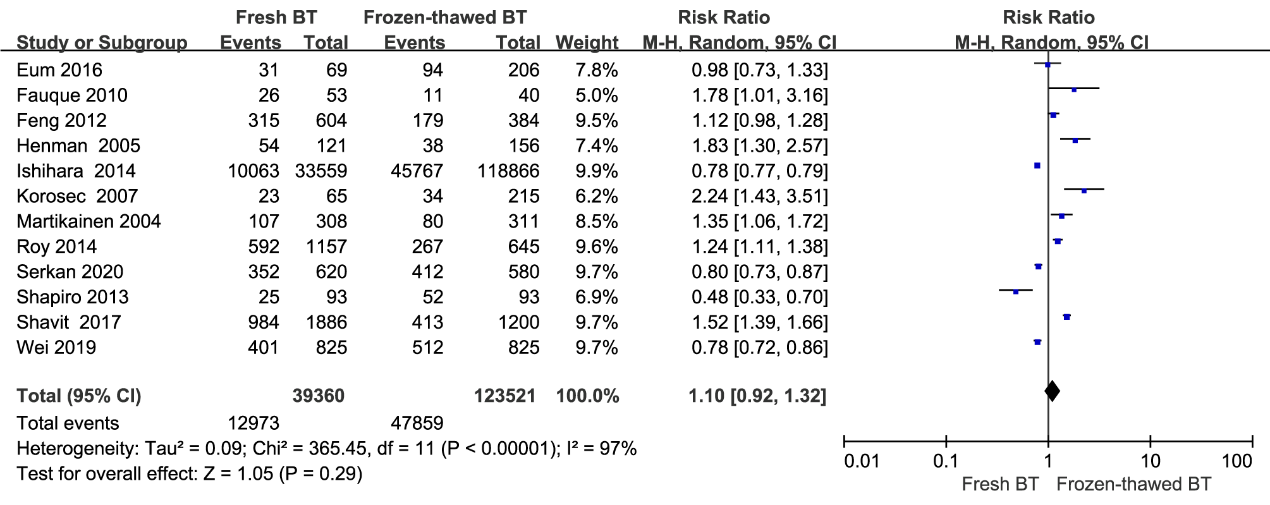


E
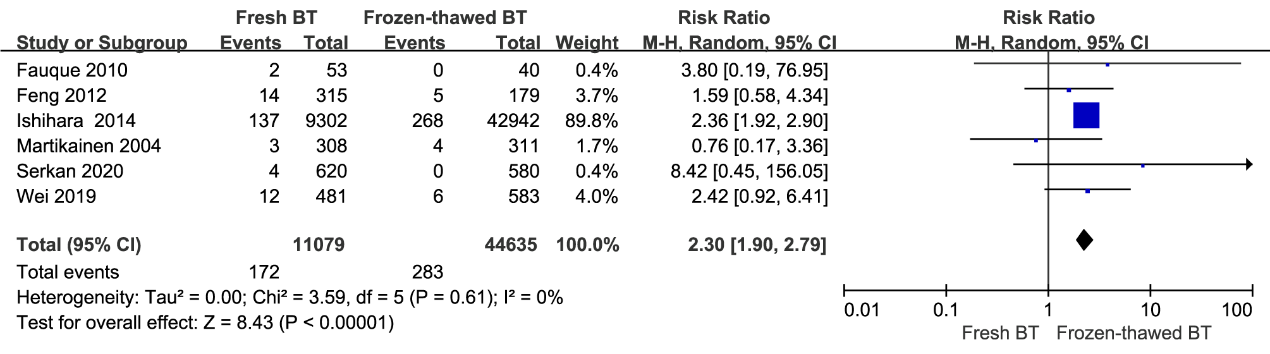


**Appendix 36**: Forest plot of comparison for women undergoing single blastocyst transfer: (a)implantation rate, (b) pregnancy rate (c) ongoing pregnancy rate, (d) clinical pregnancy rate and (e) ectopic pregnancy rate
