# Supplementary material for: Which is better for mothers and babies: fresh or frozen-thawed blastocyst transfer?
Source: BMC Pregnancy Childbirth. 2020 Sep 23;20:559. doi: 10.1186/s12884-020-03248-5 (PMC7513314; doi:10.1186/s12884-020-03248-5)
Supplement: Supplementary file 9 — Additional file 9: Appendix 37–49. Sensitivity analysis. [file 12884_2020_3248_MOESM9_ESM.docx]

**Sensitivity analysis**

**Appendix 37**: The sensitivity analysis of **implantation rate** showed the pooled proportion when the studies omitted step by step

**Appendix 38**: The sensitivity analysis of **pregnancy rate** showed the pooled proportion when the studies omitted step by step

**Appendix 39**: The sensitivity analysis of **ongoing** **pregnancy rate** showed the pooled proportion when the studies omitted step by step

**Appendix 40**: The sensitivity analysis of **ongoing pregnancy rate** showed the pooled proportion when the studies omitted step by step

**Appendix 41**: The sensitivity analysis of **clinical pregnancy rate** showed the pooled proportion when the studies omitted step by step

**Appendix 42**: The sensitivity analysis of **ectopic pregnancy rate** showed the pooled proportion when the studies omitted step by step

**Appendix 43**: The sensitivity analysis of **PIH and pre-eclampsia** showed the pooled proportion when the studies omitted step by step

**Appendix 44**: The sensitivity analysis of **GDM** showed the pooled proportion when the studies omitted step by step

**Appendix 45**: The sensitivity analysis of **placental abruption** showed the pooled proportion when the studies omitted step by step

**Appendix 46**: The sensitivity analysis of **PTD** showed the pooled proportion when the studies omitted step by step

**Appendix 47**: The sensitivity analysis of **LGA** showed the pooled proportion when the studies omitted step by step

**Appendix 48**: The sensitivity analysis of **SGA** showed the pooled proportion when the studies omitted step by step

**Appendix 49**: The sensitivity analysis of **LBW** showed the pooled proportion when the studies omitted step by step
