# Supplementary material for: Which is better for mothers and babies: fresh or frozen-thawed blastocyst transfer?
Source: BMC Pregnancy Childbirth. 2020 Sep 23;20:559. doi: 10.1186/s12884-020-03248-5 (PMC7513314; doi:10.1186/s12884-020-03248-5)
Supplement: Supplementary file 10 — Additional file 10: Appendix 50. Meta regression. [file 12884_2020_3248_MOESM10_ESM.docx]

**Meta regression**

| **Variable** | **Regression coefficient** | **Standard error** | **95% CI** | **P-value** |
| --- | --- | --- | --- | --- |
| Effect of design on IR | 0.098 | 0.205 | -.5455557 .3498034 | 0.642 |
| Effect of vitrification on IR | 0.181 | 0.255 | -.3791355 .7417641 | 0.491 |
| Effect of slow-freezing on IR | 0.103 | 0.193 | -.3215179 .5282638 | 0.603 |
| Effect of artificial cycle on IR | 0.077 | 0.169 | -.2944624 .4489882 | 0.656 |
| Effect of natural cycle on IR | -0.321 | 0.119 | -.5826854 -.0590548 | 0.021* |
| Effect of high ovarian response on IR | 0.095 | 0.245 | -.4397439 .6304597 | 0.705 |
| Effect of single BT on IR | 0.285 | 0.194 | -.1380811 .7079651 | 0.168 |
| Effect of design on PR | 0.035 | 0.131 | -.2453614 .3150892 | 0.793 |
| Effect of vitrification on PR | 0.272 | 0.108 | .0382854 .5047252 | 0.026* |
| Effect of slow-freezing on PR | -0.070 | 0.082 | -.2465247 .1072467 | 0.410 |
| Effect of artificial cycle on PR | 0.054 | 0.123 | -.2123116 .3210487 | 0.667 |
| Effect of natural cycle on PR | -0.097 | 0.079 | -.2671036 .0739461 | 0.243 |
| Effect of high ovarian response on PR | 0.070 | 0.194 | -0.3468869 0.4873525 | 0.723 |
| Effect of single BT on PR | 0.070 | 0.194 | -.3468869 .4873525 | 0.723 |
| Effect of design on OPR | 0.128 | 0.163 | -.2283077 .4837964 | 0.450 |
| Effect of vitrification on OPR | 0.013 | 0.200 | -.4280464 .4540507 | 0.949 |
| Effect of slow-freezing on OPR | -0.136 | 0.171 | -0.5136667 0.2412496 | 0.444 |
| Effect of artificial cycle on OPR | -0.072 | 0.170 | -.4450408 .3012847 | 0.680 |
| Effect of natural cycle on OPR | 0.028 | 0.112 | -0.2187521 0.2747091 | 0.808 |
| Effect of high ovarian response on OPR | 0.023 | 0.227 | -0.471442 0.5169542 | 0.922 |
| Effect of single BT on OPR | 0.286 | 0.196 | -0.141296 0.7140697 | 0.170 |
| Effect of design on CPR | 0.091 | 0.169 | -.256064 .438732 | 0.593 |
| Effect of vitrification on CPR | -0.156 | 0.188 | -.5437273 .2320516 | 0.415 |
| Effect of slow-freezing on CPR | -0.008 | 0.160 | -.3395344 .3229604 | 0.959 |
| Effect of artificial cycle on CPR | 0.199 | 0.158 | -.126865 .5254955 | 0.219 |
| Effect of natural cycle on CPR | -0.271 | 0.122 | -.5219504 -.0191557 | **0.036*** |
| Effect of high ovarian response on CPR | 0.216 | 0.199 | -.1932989 .6261762 | 0.287 |
| Effect of single BT on CPR | 0.243 | 0.137 | -.039447 .5257213 | 0.089 |
| Effect of design on EPR | -0.287 | 0.366 | -1.07796 .5037838 | 0.447 |
| Effect of vitrification on EPR | 0.499 | 0.282 | -.1155396 1.112917 | 0.102 |
| Effect of slow-freezing on EPR | -0.535 | 0.105 | -.764852 -.3051933 | **0.000*** |
| Effect of artificial cycle on EPR | 0.200 | 0.361 | -.5866087 .9864735 | 0.590 |
| Effect of natural cycle on EPR | -0.318 | 0.171 | -.6895512 .0539842 | 0.087 |
| Effect of design on PIH & PE | 0.047 | 0.399 | -.8160714 .909752 | 0.908 |
| Effect of vitrification on PIH & PE | 0.020 | 0.335 | -0.7102452 0.7495938 | 0.954 |
| Effect of slow-freezing on PIH & PE | -0.414 | 0.267 | -.9953633 .166601 | 0.146 |
| Effect of artificial cycle on PIH & PE | -0.431 | 0.334 | -1.157477 .2961528 | 0.221 |
| Effect of natural cycle on PIH & PE | -0.250 | 0.161 | -.6011767 .101634 | 0.147 |
| Effect of artificial cycle on GDM | -0.152 | 0.290 | -.8211912 .516406 | 0.614 |
| Effect of natural cycle on GDM | -0.113 | 0.109 | -.3642453 .1387947 | 0.332 |
| Effect of vitrification on PTD | -0.110 | 0.133 | -.3878826 .1676791 | 0.418 |
| Effect of slow-freezing on PTD | -0.107 | 0.075 | -.2632011 .0495072 | 0.169 |
| Effect of artificial cycle on PTD | -0.259 | 0.146 | -.5637841 .0462999 | 0.092 |
| Effect of natural cycle on PTD | -0.004 | 0.072 | -.1542419 .1458372 | 0.954 |
| Effect of vitrification on LGA | 0.165 | 0.150 | -.1745262 .5036088 | 0.301 |
| Effect of slow-freezing on LGA | -0.079 | 0.074 | -.2472144 .0886676 | 0.313 |
| Effect of artificial cycle on LGA | -0.096 | 0.161 | -.4598763 .267705 | 0.565 |
| Effect of natural cycle on LGA | 0.001 | 0.070 | -.157127 .1600663 | 0.984 |
| Effect of design on SGA | 0.141 | 0.213 | -.3011509 .5838423 | 0.515 |
| Effect of vitrification on SGA | -0.194 | 0.123 | -.4498829 .0619001 | 0.130 |
| Effect of slow-freezing on SGA | -0.122 | 0.067 | -.2618287 .0185485 | 0.086 |
| Effect of artificial cycle on SGA | -0.154 | 0.139 | -.4423523 .1345766 | 0.280 |
| Effect of natural cycle on SGA | -0.154 | 0.075 | -.3092233 .0006806 | 0.051 |
| Effect of vitrification on LBW | -0.092 | 0.191 | -.4945729 .309633 | 0.634 |
| Effect of slow-freezing on LBW | -0.122 | 0.097 | -.325388 .0820344 | 0.225 |
| Effect of artificial cycle on LBW | -0.130 | 0.194 | -.54006 .2805243 | 0.514 |
| Effect of natural cycle on LBW | -0.122 | 0.093 | -.317427 .07357 | 0.206 |
